# Discovery of spectabilide A, a new cytotoxic cyclic lipodepsipeptide from *Trichothecium spectabile* comb. nov. (*Ascomycota*, *Sordariomycetes*) revealed by OSMAC-guided metabolomics

**DOI:** 10.3897/imafungus.17.192814

**Published:** 2026-06-24

**Authors:** Gioele Pecin, Victor Gonzalez-Menendez, Jesus Martin, Frederick Annang, Thomas Andrew Mackenzie, Fernando Reyes, Olga Genilloud

**Affiliations:** 1 Fundación MEDINA, PTS Health Sciences Technology Park, Granada, Spain Fundación MEDINA Granada Spain

**Keywords:** Cyclic lipodepsipeptides, cytotoxicity, fungal epiphyte, metabolomics, mycotoxins, natural products, OSMAC, *Stanjemonium spectabile, Trichothecium spectabile*

## Abstract

The biosynthetic potential of the fungal epiphyte *Stanjemonium spectabile*, isolated from the endemic plant *Bupleurum gibraltaricum* (Granada, Spain), was investigated combining OSMAC (One Strain, Many Compounds) and metabolomic approaches. A total of 78 cultivation conditions were evaluated using different media formulations based on substrate utilization profiles obtained from filamentous fungi nutritional array plates. Chemical dereplication and MS/MS-based molecular networking revealed the production of a broad array of mycotoxins and bioactive compounds, including a novel cyclic lipodepsipeptide, namely spectabilide A. Phylogenetic studies, combined with a chemotaxonomic analysis of the annotated compounds, also supports that *S. spectabile* resides in the *Trichothecium* s.str. clade.

The isolated compound spectabilide A, composed of three amino acids and a hydroxylated aliphatic side chain, displayed potent cytotoxic activity against breast adenocarcinoma cancer cell lines. These findings highlight the potential of underexplored fungal taxa and cultivation strategies for the discovery of novel bioactive scaffolds and provide new insights into the chemical ecology and taxonomy of *S. spectabile*, resulting in the new combination *Trichothecium spectabile***comb. nov**.

## Introduction

Plant-associated fungi have proved to be a relevant source for the production of bioactive molecules, including antibiotics, phytohormones and pesticides, many of them generated in response to the interactions with their host (Hashem et al. 2023). Beauvericin (Al Samra et al. 2025), phaeofungin (Singh et al. 2012), jesterone (Li and Strobel 2001) and phomoxanthone (Böhler et al. 2018) are just a few examples of compounds isolated from plant-associated fungi that show a strong cytotoxic or antimicrobial activity. Bioprospections of endemic and rare xerophilic plants have been carried out on the arid zones of Andalusia (Spain), revealing that most of the fungi associated with the plants in this area are yet to be discovered (Bills et al. 2012; González-Menéndez et al. 2018). The presence of many endemic plants in these areas or poorly represented elsewhere suggests the possibility of discovering new plant associated fungi, as potential producers of new secondary metabolites (SMs). Despite their metabolic biosynthetic potential, it is well established that most natural products’ biosynthetic pathways remain inactive in standard laboratory conditions, requiring novel methods to activate this cryptic metabolism (Scherlach and Hertweck 2009). One of the most successful culture-based strategies to promote a fungus chemical diversity is certainly the “one strain - many compounds (OSMAC)” approach (Bode et al. 2002). The strategy consists of systematically changing the cultivation parameters, thereby forcing the studied microorganism to adapt, eventually expressing genes that are activated only under precise environmental conditions. The OSMAC approach has proven to be effectively activating silent genes in several fungal species, including plant-associated fungi (Paranagama et al. 2007; Scherlach and Hertweck 2009; Hewage et al. 2014; Farinella et al. 2021). Along with the OSMAC approach, other strategies to stimulate the production of SMs in filamentous fungi have been studied in the last decade such as, the addition of adsorptive polymeric resins and epigenetic modifiers to the cultivation medium. Adding small amounts of adsorptive polymeric resins, such as XAD-16 and HP-20, to the fermentation media can, in fact, improve the production of certain metabolites by capturing them, preventing both their degradation and toxic effects for the cell and preventing any negative regulatory effect by the final product (Marshall et al. 1990; Phillips et al. 2013; González-Menéndez et al. 2014). On the other hand, it has been demonstrated that the perturbation of the normal state of chromatin in filamentous fungi can interfere with its regulatory functions, thus sometimes leading to the activation of cryptic regions of the chromosome (Brosch et al. 2008). Epigenetic modifiers like the DNA methyltransferase (DNMT) inhibitor hydralazine hydrochloride or the histone deacetylase (HDAC) inhibitor suberoylanilide hydroxamic acid (SAHA) have been successfully used to modulate the chromatic dynamics and stimulate the expression of silent genes (Scherlach and Hertweck 2009; Cichewicz 2010; González-Menéndez et al. 2016). Finally, the dereplication of known molecules is a key process in natural product discovery, especially important to avoid the re-discovery issue. Chemical dereplication of known molecules in the extracts is easily achieved with the current, relatively low cost and sensitive analytical techniques, using in-house databases of UV, HR-MS and MS/MS spectral data. In addition to in-house databases, the advent of open-access databases and networking tools like GNPS has allowed to analyse whole datasets against global libraries. (Greco et al. 2019; Kildgaard et al. 2014; Wang et al. 2016)

During our bioprospecting campaign to collect plant-associated fungi in the arid areas of Andalusia, a fungal strain initially identified as *Stanjemonium spectabile* was isolated as an epiphyte of *Bupleurum gibraltaricum*, an endemic plant in southern Spain and Morocco. The fact that the fungus was unusually isolated as epiphyte, if compared to other soil borne *Stanjemonium* species, was what pushed us to investigate the metabolic potential of this isolate and its holotype strain CBS 340.70T following an OSMAC approach. In this work we propose the reclassification of *S. spectabile* CF-278320 and its ex-type strain CBS 340.70 into the genus *Trichothecium* based on phylogenetic and chemotaxonomic analyses, and report the discovery of a new cyclic lipodepsipeptide with biological activity against various cancer cell lines.

## Methods

### Strain isolation and identification

The epiphyte fungal strain CF-278320 was directly isolated from conidiophores developed on dead leaves of the endemic plant *Bupleurum gibraltaricum* collected in Embalse de Canales, Granada, Spain in July 2011 by incubation in a moist chamber. The isolate was cultured on YM agar (malt extract 10 g, yeast extract 2 g, agar 20 g, 1000 mL distilled H_2_O) for 14 days at 22 °C, to study the macroscopic and microscopic characteristics. Strain designated with unique ID (CF-278320), was preserved as frozen conidia and mycelia in 10% glycerol at −80 °C and maintained in Fundación MEDINA’s fungal culture collection. DNA extraction, PCR amplification and DNA sequencing were performed as previously described (Gonzalez-Menendez et al. 2017). DNA sequence of the complete ITS_1_-5.8S-ITS_2_-28S region or independent ITS and partial 28S rDNA sequences were compared with sequences at GenBank®, the NITE Biological Resource Center (http://www.nbrc.nite.go.jp) and CBS strain database (http://www.westerdijkinstitute.nl) by using the BLAST® application.

### Phylogenetic analysis

To determine the phylogenetic position of our isolate, sequences from previous work on the *Acremonium* phylogenetic overview and revision of *Gliomastix*, *Sarocladium*, and *Trichothecium* (Summerbell et al. 2011) and other available related sequence were downloaded from GenBank (https://www.ncbi.nlm.nih.gov/genbank/) to generate the phylogenetic trees (Suppl. material 1: tables S3, S5). Sequence alignments of the four individual loci (ITS, LSU, rpb2, tef-1α) were generated with Bioedit v. 7.7.1 (Hall 1999) and were then manually edited in MEGA v. 12.0.11 (Kumar et al. 2016). Both Maximum Likelihood (ML) and Bayesian analysis (BA) were used for phylogenetic inferences of individual sequence alignments, followed by the concatenated alignments. Species and genus affinities of *Stanjemonium spectabile* were inferred from a Bayesian analysis using the Markov Chain Monte Carlo (MCMC) approach with MrBayes 3.0163. To improve mixing of the chains, four incrementally heated simultaneous Monte Carlo Markov chains were run over 2 × 10^6^ generations. Hierarchical likelihood ratio tests with the MrModeltest® 2.2 software (Nylander 2004) were used to calculate the Akaike Information Criterion (AIC) of the nucleotide substitution models. The model selected by AIC for the alignment was GTR + I + G that is based on six classes of substitution types, a portion of invariant alignment positions and mean substitution rates, varied across the remaining positions according to a gamma distribution. The MCMC processes were followed by a Dirichlet process prior (DPP) to obtain the substitution rates and nucleotide frequencies, and a unification of the rate parameter for the gamma distribution. The MCMC analysis was performed using a sampling frequency parameter of 100 and the first 1.000 trees were discarded before the majority rule consensus tree was calculated. In addition, Maximum Likelihood method (ML) and ultrafast bootstrap support values for phylogenetic trees were assessed calculating 1000 replicates with IQ-TREE software (Nguyen et al. 2014). For the single locus ITS phylogenetic tree, all parameters were estimated with this software selecting TIM2e+G4 nucleotide substitution model, assuming a shape parameter of the Invar + Gamma distributed substitution rates (gamma shape alpha = 0.4605) to accommodate rate variations among sites and an estimation of nucleotide frequencies as A = 0.25, C = 0.25, G = 0.25 and T = 0.25. For the multi-loci ITS, LSU, tef-1α and rpb2 phylogenetic tree, the parameters were estimated with IQ-TREE software selecting GTR+FO nucleotide substitution model, assuming a shape parameter of the Invar + Gamma distributed substitution rates (gamma shape alpha = 0.2729) to accommodate rate variations among sites and an estimation of nucleotide frequencies as A = 0.24, C = 0.27, G = 0.26 and T = 0.22.

### Nutritional requirements assay using Biolog FF MicroPlates

The FF MicroPlates™ (Biolog, Hayward, CA) were used to study the nutritional requirements of the strains. FF MicroPlates consists of a panel of 96 wells containing different carbon and nitrogen sources, including monosaccharides, poly- and oligosaccharides, and nitrogen-containing compounds (amino acids, amines, etc.). The redox dye Iodonitrotetrazolium violet (INT) present in the wells is used to colorimetrically measure the increase in metabolic activity due to the consumption of carbon sources. Through an irreversible reaction, the INT dye is converted to formazan, which has an absorbance maximum at 490 nm. In this way, an increase in metabolic activity in the presence of a given substrate can be measured by the increase in absorbance (Papaspyridi et al. 2010). By additionally measuring the absorbance at 750 nm, length at which the mycelium spectrum of absorbance is higher, the corrected values of metabolic activity can be obtained by subtracting the measurements at 490 nm from those at 750 nm (Kubicek et al. 2003).

To prepare the FF MicroPlates cultures were inoculated on 90 mm petri dishes with the nutrient-deficient media SNA (Paredes 2024) and CMA for 14–21 days to stimulate sporulation. Highly sporulating cultures were harvested by scraping the surface of the plate with sterile plastic loops and the addition of Tween 80 saline solution (Tween 80 2.5% v/v, NaCl 0.5% w/v). Spores were filtered, centrifuged, counted in Neubauer chamber and resuspended in carboxymethyl cellulose (CMC) 0.5% v/v to obtain a 10^6^ spores/mL suspension, subsequently used to inoculate 100 µL per well. FF MicroPlates were incubated in the dark at 25 °C and 70% RH for 96 hours, recording the absorbance values at 24, 48, 72, and 96 hours. Absorbance was measured in an ENVISION™ Multilabel Reader spectrofluorometer (PerkinElmer, Waltham, MA, USA).

### Fungal fermentations

Fresh cultures of the *S. spectabile* strains were obtained by collecting pure cultures from -80 °C glycerol stocks and growing them on 55 mm petri dishes with 10 mL YM (yeast extract Difco^TM^ 1 g, malt extract Difco^TM^ 10 g, agar 20 g, and 1000 mL deionized H_2_O) at 22 °C for 14–20 days. All production media used for the OSMAC approach, and the solid-state fermentation time course were inoculated from a culture in SMYA seed medium grown for 7 days at 22 °C in an orbital shaker (200 rpm; 1.5 cm throw) and prepared as previously described (Georgousaki et al. 2020).

Eight of the media selected for the OSMAC study were already described in literature: BRFT, CYS80, DEX-SOY, M104T, MV8, Wheat-1, WS80, and YES (Suppl. material 1: table SS1). The remaining seven media were designed specifically for this experiment: DEGSY, FGY-2, FOF, SM, SXSY, XYFUGA, YEC (Suppl. material 1: table SS1). All OSMAC fermentations were prepared in 40 mL EPA vials with 10 mL of each medium, inoculated with 0.3 mL of seed culture and incubated at 22 °C, 70% RH, only shaking the submerged cultures at 220 rpm. The epigenetic modifier SAHA was added at a concentration of 100 µM, and polymeric resin XAD-16 at a concentration of 3% v/v and prepared as previously described by González-Menéndez et al. in 2014. The solid-state fermentations based on rice (BRFT) and wheat (Wheat-1) were inoculated using 1 mL of SMYA inoculum and incubated in static conditions for at 22 °C, 70% RH.

The fermentation conditions for the purification of the novel compound were performed using the best producing solid-state fermentation medium BRFT. Cultures from SMYA seed medium were inoculated in Erlenmeyer flasks containing BRFT, testing two different formats: 50 mL medium in 250 mL flasks inoculated with 2 mL seed culture, and 100 mL medium in 500 mL flasks inoculated with 4.5 mL seed culture. The cultures were incubated under static conditions for 7, 10, 14 ,17, 21, 25 or 28 days, at 22 °C and 70% RH.

### Extracts harvesting and processing

EPA vial format fermentations were harvested and extracted by adding 10 mL of acetone to the 10 mL whole broth, breaking the mycelium of solid-state fermentation media like BRFT and Wheat-1 with a spatula, and shaking at 200 rpm for 3 h. The vials were centrifuged, and 12 mL of supernatant decanted into clean tubes and mixed with 0.64 mL of DMSO. Samples were then evaporated under a nitrogen stream to a volume of 3.2 mL (20% DMSO v/v) and final concentration of 2×WBE (Whole Broth Equivalent). Vials with added XAD-16 resin were extracted and evaporated a second time with 100% acetone, finally concentrating the extracted volume in the same tubes of the first extraction. Solid-state BRFT fermentations used to evaluate the best production conditions were processed by adding 1:1 v/v of acetone to the flasks, breaking the mycelia with a spatula and shaking at 200 rpm for 3 h. The fermentations were then centrifuged, and an aliquot of 20 mL was decanted into clean EPA vials to be processed as previously described.

#### Chemical dereplication

Extracts were analysed by LC/MS and searched against Fundación MEDINA’s proprietary database using both, LC-LRMS data searching for retention time, UV spectrum, MS(+) and MS(-), or LC-HRMS data using retention time, accurate mass, and fragmentation patterns, to dereplicate known compounds (Martín et al. 2014; González-Menéndez et al. 2016; Pérez-Victoria et al. 2016). The molecular formula (MF) of the components was assigned using SmartFormula and SmartFormula3D softwares from Bruker’s Data Analysis application. Secondary metabolites whose predicted molecular formulae were not identified within the internal database were searched against the Dictionary of Natural Products (DNP, v25.1) and The Natural Products Atlas (Poynton et al. 2024) databases.

#### Metabolic analysis (MS/MS data, MN)

LC-MS/MS data were converted to mzXML format using MSConvert software (Chambers et al. 2012). With the converted data, a molecular network (MN) was created on the GNPS1 website (http://gnps.ucsd.edu). The data was filtered by removing all MS/MS fragment ions within +/- 17 Da of the precursor m/z. MS/MS spectra were window filtered by choosing only the top 6 fragment ions in the +/- 50 Da window throughout the spectrum. The precursor ion mass tolerance was set to 0.1 Da and a MS/MS fragment ion tolerance of 0.02 Da. A network was then created where edges were filtered to have a cosine score above 0.7 and more than 6 matched peaks. Further, edges between two nodes were kept in the network if, and only if, each of the nodes appeared in each other’s respective top 10 most similar nodes. Finally, the maximum size of a molecular family was set to 100, and the lowest scoring edges were removed from molecular families until the molecular family size was below this threshold. The spectra in the network were then searched against GNPS’ spectral libraries. The library spectra were filtered in the same manner as the input data. All matches kept between network spectra and library spectra were required to have a score above 0.7 and at least 6 matched peaks (Wang et al. 2016). The results of the MN can be found at https://gnps.ucsd.edu/ProteoSAFe/status.jsp?task=ede7a1d3c8244b0abc741acf8630c23c. To visualize the cluster maps of the results, the data were then exported to Cytoscape® (version 3.10.3) (Shannon et al. 2003).

#### Purification of spectabilide A

A 1.5 L culture of *S. spectabile* in BRFT medium was extracted by addition of an equal volume of 1.5 L of Milli-Q water. The mixture was stirred to create a homogeneous aqueous suspension. This was then extracted by shaking with 1.5 L acetone in a Kuhner shaker at 200 rpm, 24 °C, for 2 h. The extract was filtered, and the acetone evaporated under a heated nitrogen stream to obtain a concentrated crude. This crude was extracted two times with 1.5 L of ethyl acetate and the organic phases were combined and dried. This extract was re-dissolved in methanol and mixed with double amount of C-18 reversed-phase silica gel. The mixture was evaporated to dryness in a rotary evaporator, after which the residue was loaded onto a C-18 reversed-phase column that was eluted at 18 mL/min flow rate with a linear gradient of 5–100% CH_3_CN/H_2_O (total run time was 54 min). 55 fractions (20 mL each) were collected and subjected to LC/MS analysis to confirm the presence of spectabilide A. The fraction containing the compound was subsequently subjected to repeated semi-preparative reversed-phase HPLC (linear gradient 5–100% CH_3_CN/H_2_O in 45 min, using an Agilent Zorbax SB-C18 column (9.4 × 250 mm, 5 µm) at 3.6 mL/min flow rate with UV detection at 210 and 280 nm).

**Spectabilide A**. White, amorphous solid; (+)-ESI-TOF MS *m/z* 553.3606 [M + H]+ (calcd. for C_28_H_49_N_4_O_7_^+^, 553.3596); ^1^H and ^13^C NMR data in methanol-*d*_4_, see Table 1.

**Table 1.** NMR spectroscopic data (methanol-*d*_4_, 500 MHz for ^1^H, 125 MHz for ^13^C) of Fig. 6.

	Position	δ ^1^H, m, J (Hz)	δ ^13^C, Mult	HMBC (H to C)
L -Homoglutamine	1	4.24, dd (8.5, 5.4)	55.3, CH	C2, C3, CO, CO β-Ala
	2a	1.86, m	31.9, CH_2_	C1
	2b	1.75, m		C1, C3
	3a	1.75, m	23.4, CH_2_	C2, C4
	3b	1.67, m		C2, C4, C5
	4	2.25, brd t (7.0)	35.71, CH_2_	C2, C3, C5
	5		178.3, C	
	CO		173.4, C	
β-Alanine	1a	2.51, ddd (16.6, 4.8, 2.3)	34.9, CH_2_	C2, CO
	1b	2.43, ddd (16.6, 10.8, 3.0)		
	2a	3.79, m	36.0, CH_2_	C1, CO, CO DHA
	2b	3.27, ddd (13.4, 10.8, 2.3)		C1, CO, CO DHA
	CO		175.2, C	
Dehydroalanine	1		139.0, C	
	2a	5.75, s	115.0, CH_2_	CO
	2b	5.28, s		C1, CO
	CO		166.1, C	
DHDMTDA	1		177.0, C	
	2	2.66, qd (7.0, 4.6)	47.7, CH	C1, C3, C4, C15
	3	3.77, m	69.0, CH	C-1, C4, C5
	4a	1.99, ddd (14.2, 10.8, 1.7)	33.2, CH_2_	C5
	4b	1.52, ddd (14.2, 10.7, 1.9)		C3
	5	5.14, ddd (10.8, 4.9, 1.8)	76.8, CH	CO Homo-Gln, C3, C4, C6, C7, C16
	6	1.75, m	38.2, CH	
	7a	1.40, m	33.6, CH_2_	
	7b	1.14, m		C5
	8a	1.40, m	28.3, CH_2_	
	8b	1.28, m		
	9	1.24-1.34*	30.9, CH_2_	
	10	1.24-1.34*	31.1, CH_2_	
	11	1.24-1.34*	30.6, CH_2_	
	12	1.24-1.34*	33.2, CH_2_	
	13	1.24-1.34*	23.9, CH_2_	
	14	0.90, t (6.6)	14.6, CH_3_	C12, C13
	15	1.10, d (7.0)	11.4, CH_3_	C1, C2, C3
	16	0.89, d (6.8)	15.5, CH_3_	C5, C6, C7

*Overlapping signals.

#### Marfey’s analysis of the novel compound

A sample of 200 μg of pure compound was dissolved in 0.4 mL of 6N HCl and heated at 110 °C for 16 h. The hydrolysate was then evaporated to dryness under a N_2_ stream, and the residue dissolved in 100 μL of water. Then, a 100-μL aliquot of 1% (w/v) solution of D-FDVA (N-(2,4-dinitro-5-fluorophenyl)-D-valinamide) in acetone was added to the aqueous solution of the compound hydrolysate and to a 50-μL aliquot of a 50-mM solution of each aminoadipic acid enantiomer (DL mixture or L) and, after the addition of 20 μL of 1 M NaHCO_3_ solution, each mixture was incubated at 40 °C, 800 rpm for 60 min. After the incubation, the reactions were quenched by the addition of 10 μL of 1N HCl, and the crude mixtures were diluted with 700 μL of acetonitrile to be analysed by LC/MS on an Agilent 1100 single quadrupole. Separations were carried out on a Waters XBridge C18 column (4.6 × 150 mm, 5 μm) maintained at 40 °C. A mixture of two solvents, A (10% acetronitrile, 90% water) and B (90% acetronitrile, 10% water), both containing 1.3 mM trifluoroacetic acid and 1.3 mM ammonium formate, was used as the mobile phase under a linear gradient elution mode (25−55% B in 30 min, 55−100% B in 1 min, isocratic 100% B for 4 min) at a flow rate of 1 mL/min.

#### Bioactivity characterization

Bioactivity of the purified compound was evaluated against five different human cancer cell lines: human hepatocellular carcinoma HepG2 (ATCC HB-8065), breast adenocarcinoma MCF-7 (ATCC HTB-22), pancreas carcinoma MIA PaCa-2 (ATCC CRL-1420), skin melanoma A2058 (ATCC CLR-11147), and lung carcinoma A549 (ATCC CCL-185). The activity was evaluated by MTT reduction colorimetric assays (Mosmann 1983) in a high-throughput 96-well-plate format, testing the isolated compound in triplicate, in 10-point dose-response curves with a starting concentration of 50 µM and following ½ serial dilutions for 72 hours, as previously described by de Pedro et al. (2012). ED_50_ values were determined for each cell line using Genedata Screener® (Cautain et al. 2015).

### General experimental procedures

1D and 2D NMR spectra were recorded on a Bruker Avance III spectrometer (500 and 125 MHz for ^1^H and ^13^C NMR, respectively) equipped with a 1.7 mm TCI MicroCryoProbe (Bruker Biospin, Fällanden, Switzerland). Chemical shifts were reported in ppm using the signals of the residual solvents as internal reference (*δ*_H_ 3.31 and *δ*_C_ 49.1 for methanol-*d*_4_).

LC-UV-MS measurements were carried out using an Agilent 1100 LC-MS instrument equipped with a single quadrupole detector. Chromatographic separation was achieved on a Zorbax SB-C8 column (2.1 × 30 mm, 5 μm particle size) operated at 40 °C with a mobile-phase flow rate of 300 μL/min. The mobile phase consisted of solvent A (water/acetonitrile, 90:10, v/v) and solvent B (water/acetonitrile, 10:90, v/v), both containing 1.3 mM trifluoroacetic acid (TFA) and ammonium formate. A linear gradient was applied from 10% to 100% solvent B over 6 min, followed by a 2 min hold at 100% B, and re-equilibration to initial conditions over 2 min. UV detection was performed using diode-array scanning across 100–900 nm with 4 nm resolution and a scan interval of 0.25 s. Ionization of the eluent was achieved via electrospray using the standard Agilent source, operated with a drying gas flow of 11 L/min at 325 °C, a nebulizer pressure of 40 psig, and a capillary voltage of 3.5 kV. Mass spectra were acquired in full-scan mode over an *m/z* range of 150–1500, with a scan cycle time of 0.77 s, in both positive and negative ionization modes. High-resolution ESI and tandem MS analyses were conducted on a Bruker maXis QTOF mass spectrometer interfaced with the same HPLC system, operating in positive ESI mode at a capillary voltage of 4 kV, drying gas temperature of 200 °C with a flow rate of 11 L/min, and a nebulizer pressure of 2.8 bar. External mass calibration was performed prior to analysis using TFA-Na cluster ions, with pre-run calibration achieved by direct infusion of the same standard. Analytical-grade acetone was employed for extraction procedures, while all solvents used during compound isolation were of HPLC quality.

## Results

### Strain isolation and identification

**Figure 1.**
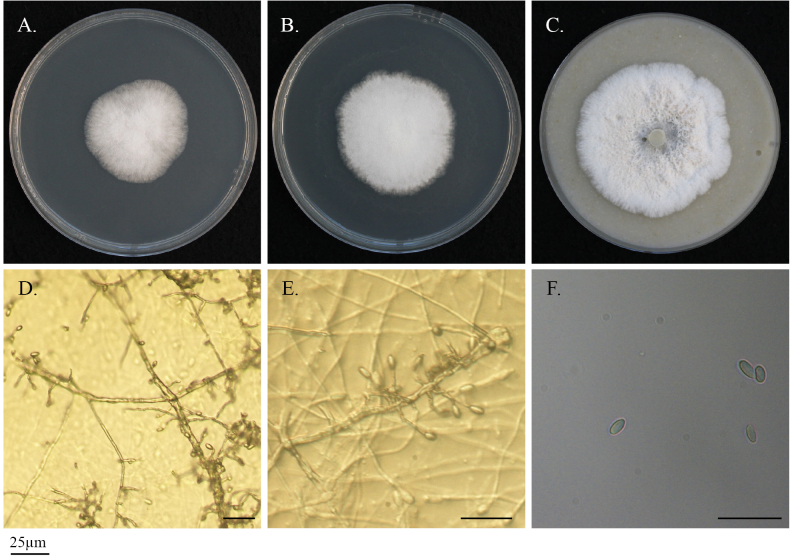
**A–C**S. spectabile CF-278320 colonies on YME, PDA and OA plates, respectively, at 12 days of cultivation; **D, E**S. spectabile CF-278320 conidiophores observed on YME medium at 21 days of cultivation; **F** conidia from CMA observed at 14 days of cultivation. Scalebar is 25 µm (D-F).

The strain CF-278320 was isolated as an epiphyte from conidiophores developed on dead leaves of the endemic plant *B. gibraltaricum* collected from arid zones of Andalucía (Embalse de Canales, Granada, Spain) during a sample collection campaign. The strain was initially cultivated on YME plates at 22 °C, showing floccose-lanose mycelium with white to pale pink colour. Formation of conidia was mainly observed on nutrient-poor media such as cornmeal agar (CMA) and synthetic nutrient agar (SNA) after approximately 14 days of cultivation. Its macroscopical characteristics, in terms of colony and conidia formation, were comparable to those previously described for *S. spectabile*, (Gams 1973; Gams et al. 1998) (Fig. 1). The identification was further confirmed with the analysis of its ITS_1_-5.8S-ITS_2_ and 28S sequences (GenBank® accession number: PX069749.1), which revealed a 100% similarity with *S. spectabile* CBS 340.70T.

**Figure 2.**
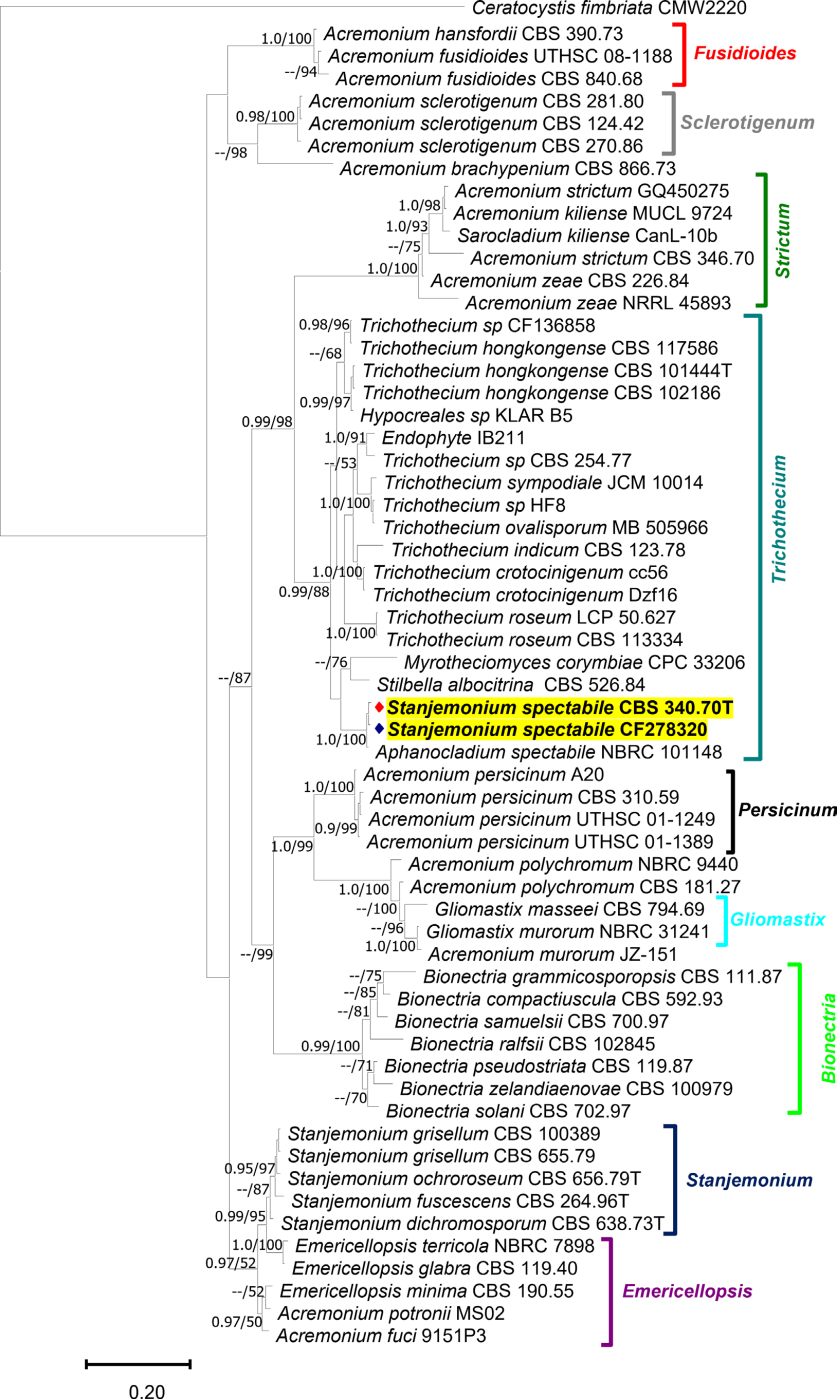
ITS Consensus tree based on Maximum Likelihood (ML) and Bayesian analyses. Probability values/ML bootstrap values are indicated respectively on the branches. Values < 0.90 are designated by “--”.

The isolation of the strain from a plant source was, however, in contrast with the origin described for all other *Stanjemonium* strains from CBS collection (https://wi.knaw.nl), which were obtained from soil sources, except for *Stanjemonium dichromosporum*, isolated from rhizosphere, and *S. spectabile* CBS 340.70T, isolated from air samples. As previously reported by Hou et al. (2023), CBS 340.70T forms a fully supported clade with species of *Trichothecium* and clearly separates from the *Stanjemonium* clade. We therefore decided to conduct a phylogenetic analysis of CF-278320 and the holotype CBS 340.70T (Westerdijk Institute collection), including several species belonging to *Trichothecium*, *Stanjemonium*, and nearby clades in the phylogenetic trees, defined based on ITS, LSU, *rpb2* and *tef1* sequences available on NCBI. The phylogenetic tree derived by a comprehensive single-locus analysis of ITS sequences from several species is shown in Fig. 2, whilst Suppl. material 1: fig. S22 shows the phylogenetic tree derived from a multi-locus analysis (ITS, LSU, tef1 and rpb2) of various closely related species that had available sequences on NCBI.

The analyses carried out with Maximum Likelihood and Bayesian models resulted in the same tree topology. In accordance with the study of Hou et al. (2023), the consensus ITS phylogenetic tree revealed that the two strains of *S. spectabile*, along with the putative conspecific *Aphanocladium spectabile* NBRC 101148, cluster within the *Trichothecium* clade, while the other *Stanjemonium* spp. remain separated and close to the *Emerecillopsis* clade (Fig. 2). The same results were obtained for *S. spectabile* CF-278320 from the consensus phylogenetic tree derived from the multi-locus analysis (Suppl. material 1: fig. S22).

### Expanding Stanjemonium spectabile chemical diversity by OSMAC approach

The lack of genomic and metabolomic information reported in literature regarding *S. spectabile* and the results suggesting its inclusion in a different phylogenetic group far from the clade of other *Stanjemonium* strains, determined the detailed study of the biosynthetic potential of the two strains CF-278320 and CBS 340.70T following an OSMAC approach. The design and selection of the best culture media required to first investigate the nutritional requirements of the strains using FF MicroPlates. The array of substrates contained in the 96 well plates is utilized differently by each fungal species and defines the utilization preferences of each carbon and nitrogen source. The substrate utilization profiles of both strains CF-278320 and CBS 340.70T are reported in Suppl. material 1: figs S2–S5 (Supplementary material). Notably, the profiles of the two strains differ one to the other, in terms of both preferred substrates and metabolic activity (measured with a colorimetric assay). We selected carbon sources and nitrogen-containing substrates based on two criteria: the inclusion of substrates that are rapidly utilized and utilized secondarily in later stages of growth by the fungus, and the immediate commercial availability of the substrates. The ten selected carbon sources included sucrose, fructose, xylose, maltose, dextrin, glucose, D-sorbitol, D-cellobiose, N-acetyl-D-glucosamine, and glycerol. Five nitrogen-including substrates included L-proline, succinic acid, L-glutamic acid, L-aspartic acid, and L-serine. We selected eight media from literature that included some of the previously chosen carbon sources and nitrogen-containing substrates (BRFT, CYS80, DEX-SOY, M104T, MV8, Wheat-1, WS80, and YES). In addition, seven new media were specifically designed for the experiment: DEGSY, FGY-2, FOF, SM, SXSY, XYFUGA, YEC. Some of the media included complex substrates like oat (FOF), soy (DEGSY, Dex-Soy, MV8), wheat (Wheat-1, WS80), corn flour (CYS80), brown rice (BRFT), and V8 juice (MV8), while other media’s main carbon sources were simple saccharides (FGY-2, M104T, SM, XYFUGA). Five media (CYS-80, FOF, MV8, SXSY, WS80) were tested in duplicate, adding in one condition the epigenetic modifier SAHA. Similarly, another six media (DEX-SOY, FGY-2, SM, XYFUGA, YEC and YES) were tested in duplicate with the addition of the adsorptive polymeric resin XAD-16 to one of the conditions (Suppl. material 1: table SS1). Finally, for each of the 26 formulations mentioned above, three incubation times, 7, 14, and 21 days, were included to expand the timeframe of different metabolites production. The OSMAC-based media study globally encompassed a total of 78 fermentation conditions for each strain (CF-278320 and CBS 340.70T) (Suppl. material 1: fig. S6).

### Chemical analysis of the metabolic diversity

The chemical profiles of the extracts coming from each fermentation condition were analysed by low resolution UV-LC-LRMS and, at this stage, no molecules matching our internal database were found. The UV-LC-LRMS profiles were then used to select a total of 20 conditions to be analysed with LC-HRMS and MS/MS fragmentation (Suppl. material 1: table S2). The selection was based on the abundance and intensity of the peaks present on the single chromatograms, which indicate a higher chemical diversity in the extracts, and including at least one condition for each medium. Since the culture media containing polymeric resin XAD-16 did not present significant changes in the LC-LRMS spectra when compared with the respective control media, these conditions were not considered for further analyses.

LC-HRMS and MS/MS fragmentation analyses allowed to assign accurate molecular formulas (MFs) to the components that did not match our internal database. The annotation of unmatched compounds was performed by searching their predicted MFs against the Dictionary of Natural Products (DNP) and The Natural Products Atlas (Poynton et al. 2024) databases. Among the selected conditions, 29 putative compounds that corresponded to relevant peaks in the UV traces were annotated, and for 25 of them a putative name was given based on the predicted MFs and the database dereplication (Suppl. material 1: table S2). Most of the identified putative compounds were mycotoxins, such as trichodermol (C_15_H_22_O_3_), trichoverrol A (C_23_H_32_O_7_), verrol (C_21_H_30_O_6_), myrothecine C (C_29_H_36_O_11_), roridin A (C_29_H_40_O_9_), verrucarin J (C_27_H_32_O_8_), myrotoxin (C_29_H_34_O_11_) and verrucarin L-acetate (C_29_H_34_O_10_) Other detected putative compounds, including 8-α-acetoxyverrol (C_23_H_32_O_8_), antibiotic TAN 1746 (C_28_H_40_N_4_O_6_), emerixanthone E (C_27_H_30_O_9_), and cerebroside B (C_41_H_77_NO_9_) are reported in literature to have antifungal or antibaterial activity (Masouka et al. 2000; Meng et al. 2011; Fredimoses et al. 2018; Yuan et al. 2020). The analysis of chemical profiles also unveiled the presence of five compounds lacking putative annotations, four of which were only present in traces. However, the fifth unknown compound, a feature with a predicted molecular formula C_28_H_48_N_4_O_7_, showed relevant chromatographic UV_210nm_ and MS peaks in 7 of the 20 fermentation conditions. Out of the 29 annotated putative compounds, eight were only produced in a single media: 8-α-acetoxyverrol, penicianstinoid E, diheteropeptin and unk. comp. 1 were unique to M104T; FCRR toxin and kadhenrischinin E were unique to YEC; unk. comp. 1 and deepoxy-chlamidocin were only found in XYFUGA. Another six putative compounds were found to be present in only two media each: trichodermol, unk. comp. 3, myrothecine B, emerixanthone E, cotylenin F/G, and cerebroside B. Myrothecine E/C, unknown compound 5 and verrucarin L-acetate were the 3 most widely distributed compounds, found in 9, 13 and 20 media respectively.

**Figure 3.**
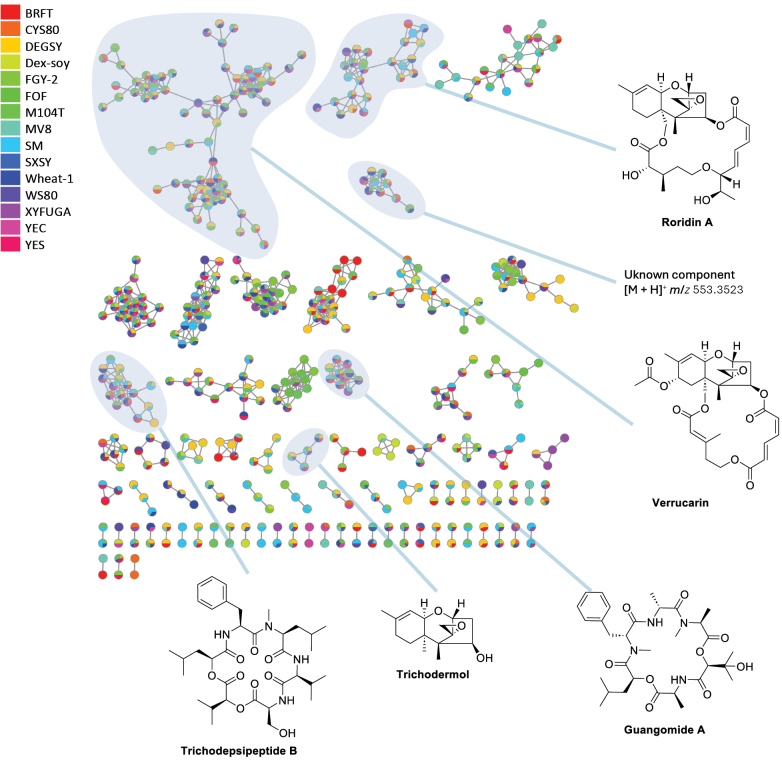
FBMN generated from the MS/MS data of the selected OSMAC extracts. Each node of the MN represents the m/z of a parent ion. The different media are represented as color-coded charts for each node. On the sides, identified clusters of the putative mycotoxins and compounds annotated.

MS/MS data were also used to generate a featured-based molecular network (FBMN) using GNPS, to better visualize and interpret the dataset, as reported in Fig. 3.The MN revealed the presence of several clusters, including those of the previously annotated toxins, and that of the unknown putative compound with MF C_28_H_48_N_4_O_7_.

**Figure 4.**
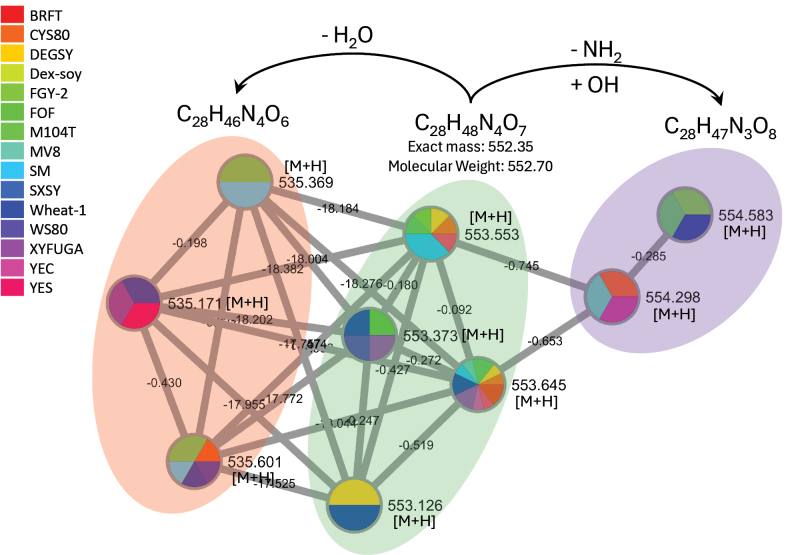
Cluster of the annotated unknown compound with molecular formula C_28_H_48_N_4_O_7_ and accurate mass of 552.35.

Moreover, the FBMN analysis revealed the presence of two putative analogs of the unknown metabolite, with tentative molecular formulae of C_28_H_46_N_4_O_6_ and C_28_H_47_N_3_O_8_, produced in traces. These could correspond to a loss of H_2_O in the first case, and the substitution of an -NH_2_ group with an -OH group in the second (Fig. 4).

**Figure 5.**
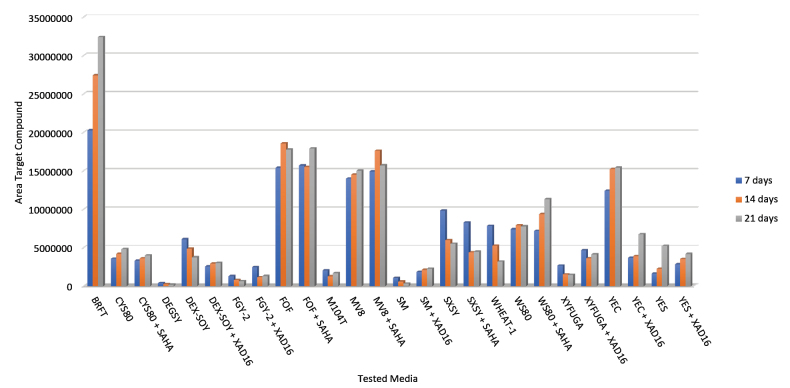
Relative production of compound C_28_H_48_N_4_O_7_ for S. spectabile strain CF-278320 on the different media, represented as area under the peak of the target compound (EIC 553.360 ± 0.005)

Changes in the production of the unknown compound C_28_H_48_N_4_O_7_ in all conditions were determined by comparing the areas under the extracted MS chromatograms for peak EIC 553.360 ± 0.005 to their corresponding control media. The production titers of the compound for strain CF-278320 were highest in the solid-state medium BRFT, followed by FOF, MV8 and YEC media (Fig. 5). Production titers in the CBS 340.70T holotype strain were instead generally lower, compared to strain CF-278320 (Suppl. material 1: fig. S9). The addition of SAHA in some media slightly increased the production of the compound, such as in MV8 and WS80, while the addition of XAD-16 resin increased its production in FGY-2, SM, XYFUGA and YES media, but strongly decreased it in YEC medium (Fig. 5). In view of the significant amounts of this compound produced in several culture media, we focused our efforts on scaling-up the cultures and performing its isolation and structural characterization.

### Definition of the best cultivation conditions for the production of compound C_28_H_48_N_4_O_7_

As mentioned above, the highest production titers of the unknown compound with molecular formula of C_28_H_48_N_4_O_7_ were obtained on the solid-state fermentation medium BRFT. This medium was selected to study the production of the compound over time with a time-course experiment with both strains of *S. spectabile*, following the production at 7 different incubation days (7, 10, 14, 17, 21, 25 and 28), and testing two larger scale formats (250 and 500 mL Erlenmeyer flasks containing 50 and 100 mL of solid medium respectively) (Suppl. material 1: fig. S10). As for the OSMAC-based experiment, the production of C_28_H_48_N_4_O_7_ was measured with UHPLC-UV by comparing the areas under the peak to their corresponding control areas at 210 nm. Again, the relative production of the compound was considerably higher for strain CF-278320 than for CBS 340.70T. The highest production titers for strain CF-278320 are reached between 14 and 21 days of fermentation with the 500 mL flask format, being the maximum value of 5 mg/L obtained at 17 days (Suppl. material 1: fig. S11).

### Taxonomy

#### Trichothecium spectabile

Taxon classification

Animalia

SordariomycetesMyrotheciomycetaceae

(W. Gams) Pecin & González-Menéndez comb. nov.

D4E81AD9-E67F-57DF-945A-8CE798AE13B5

863453

Fig. 1

##### Basionym.

*Stanjemonium spectabile* W. Gams, O’Donnell, Schroers & Christensen, *Canad. J. Bot*. 76(9): 1581 (1999) [1998].

##### Synonyms.

*Aphanocladium spectabile* W. Gams, *Persoonia* 7(2): 162 (1973).

##### Typus.

• France, Marseille, from air sample, Mar 1970, isolated by Charpin, No. 2, deposited by J. Nicot (**holotype** CBS H-6687, ex-type CBS 340.70).

##### Additional material examined.

• Spain, Embalse de Canales, Granada, epiphyte of *Bupleurum gibraltaricum* dead stem, Jul 2011, *González-Menéndez* (CF-278320).

##### Description and illustration.

W. Gams, Persoonia 7: 162. 1973 and W. Gams, O’Donnell, Schroers & Christensen, *Canad. J. Bot*. 76(9): 1581 (1999) [1998].

##### Notes.

We propose to transfer *Stanjemonium spectabile* to *Trichothecium* based on the phylogenetic analysis of the ex-type strain CBS 340.70, since this strain reside in the *Trichothecium* s.str. clade (Hou et al. 2023). Our phylogenetic analysis also confirms that the holotype and our isolate CF-278330 are members of *Trichothecium* clade (Fig. 2). In addition, our metabolomic analysis shows that the ex-type strain of the species CBS 340.70 and our isolate CF-278320, produces trichodepsipeptide B and guangomide B, characteristic chemotypes only described for members of *Trichothecium* genus (Springer et al. 1984; Sy-Cordero et al. 2011)

### Spectabilide A purification and structure elucidation

Crude extracts from the time course experiment in solid-state fermentation BRFT were processed to isolate the novel compound, designated as spectabilide A. The presence of spectabilide A in fraction 17 derived from a reversed-phase flash chromatography using C-18 Silica gel (Suppl. material 1: fig. S12) was confirmed by HPLC-HRMS and the fraction subsequently purified by repeated reversed-phase semipreparative HPLC eluting at a retention time of 22 min, yielding 3.5 mg of spectabilide A as an amorphous pale-white solid.

**Figure 6.**
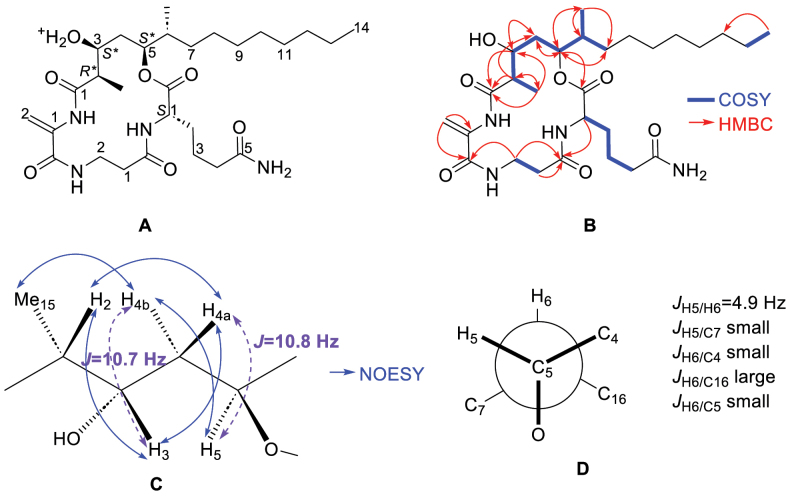
**A** Structure of spectabilide A; **B** Key COSY (bold blue) and HMBC (H to C, red) correlations observed in the structure of spectabilide A. **C** coupling constants and NOESY correlations observed around the C2-C6 fragment of DHDMTDA in spectabilide A. **D** J-based configurational analysis around the C5/C6 bond.

Its (+)-ESI-TOF spectrum displayed a protonated adduct [M + H]^+^ at *m*/*z* 553.3523 compatible with a molecular formula of C_28_H_48_N_4_O_7_ (Suppl. material 1: fig. S8). Analysis of its 1D and 2D NMR spectra (Suppl. material 1: figs S14–20) revealed signals consistent with the presence of the aminoacids L-homoglutamine (HomoGln), and β-alanine (β-Ala) (Table 1, Fig. 6). Additionally, signals corresponding to an olefinic methylene (*δ*_H_ 5.75 and 5.29), which showed HMBC correlations with carbons at *δ*_C_ 139.0 and 166.1, supported the presence of a dehydroalanine (DHA) moiety as an additional structural element of spectabilide A (Suppl. material 1: fig. S16). Furthermore, a 3,5-dihydroxy-2,4-dimethyltetradecanoic acid (DHDMTDA) unit was also identified based on COSY and HMBC correlations (Fig. 6b). Key HMBC correlations between H-1 of HomoGln and the carbonyl group of β-Ala, as well as between H-2 of β-Ala and the carbonyl of DHA established the sequence Homo Gln- β-Ala-DHA (Fig. 6b). An additional HMBC correlation between H-5 of the DHDMTDA moiety and the carbonyl of HomoGln indicated the presence of an ester bridge linking these two structural units, consistent with the downfield chemical shift of H-5 (*δ*_H_ 5.14). Although no supporting long-range HMBC correlations were observed, a second ring closure between the NH of DHA and the carbonyl group of DHDMTDA was proposed to comply with the molecular formula derived from HRMS data. On the same basis, HomoGln instead of Homoglutamic acid was proposed as a structural element of spectabilide A. An L configuration was determined for L-HomoGln through Marfey’s analysis (Suppl. material 1: fig. S21). Regarding the stereochemistry of the DHDMTDA moiety, a relative configuration of 2*R*,3*S*,5*S* was tentatively proposed based on coupling constant analysis and NOESY correlations (Fig. 6c), Thus, large coupling constants of 10.8 and 10.7 Hz for the pairs H-5/H4a and H-3/H-4b, respectively, indicated an antiperiplanar arrangement in both cases. NOESY correlations between H-2, H-3 and H-4a located these three hydrogens on the same face of the molecule (Suppl. material 1: fig. S17). Additional NOESY correlations between H-4b and H.5 and Me-15 located these groups on the other side of the molecule, completing the stereochemical assignment of this part of the molecule. Finally, *J*-based configurational analysis was applied to determine the relative configuration around the C-5/C-6 bond (Matsumori et al. 1999). The magnitudes of ^2^*J*_C,H_ and ^3^*J*_C,H_ couplings were qualitatively classified as small or large based on the intensity of the corresponding HMBC cross-peak (Hansen 1991; Pérez-Bonilla et al. 2025). The pattern of values obtained (Fig. 6D) was in agreement with an *anti* configuration and, consequently, a 6*R* relative configuration.

### Cytotoxicity bioassay

The activity of spectabilide A was evaluated against a panel of five different human cancer cell lines by means of the MTT reduction assay in a dose response curve. (Suppl. material 1: fig. S13). Spectabilide A showed potent activity against all cell lines tested, with reported ED_50_ values of 0.28 µM (95% Confidence Interval (CI) 0.26–0.30 µM) for HepG2 (hepatocellular carcinoma), 0.11 µM (95% CI 0.11–0.11 µM) for MCF-7 (breast adenocarcinoma), 0.23 µM (95% CI 0.23–0.24 µM) for MIA PaCa-2 (pancreas carcinoma), 0.13 µM (95% CI 0.13–0.13 µM) for A2058 (skin melanoma), and 0.16 µM (95% CI 0.15–0.16 µM) for A549 (lung carcinoma).

## Discussion

*Stanjemonium spectabile* was initially classified as *Aphanocladium spectabile* because of its solitarily formed conidia (Gams 1973) and later reclassified based on morphological reasons, single phialoconidia on non-persistent ampulliform phialides, and 18S rDNA sequence analyses as *Stanjemonium* (Gams et al. 1998). The revision of *Trichothecium* by Summerbell et al. (2011) highlighted that almost three different asexual stages are described in *Trichothecium* species, including phialoconidia. A more recent study on *Acremonium*-like fungi, fully supported by a phylogenetic tree based on a concatenated alignment of LSU, ITS and *rpb2* sequences, located *S. spectabile* 340.70T within the *Trichothecium* clade (Hou et al. 2023). In line with these results, our phylogenetic analyses also confirmed that the ex-type strain of *Stanjemonium spectabile* (CBS 340.70) is accommodated in *Trichothecium* s.str. clade (Fig. 2). In addition, *Myrotheciomyces corymbiae* (CPC 33206, Crous et al. 2018), was recently transferred to the genus *Trichothecium*, proposing as a new combination based on phylogenetic analysis (Chen et al. 2025), also confirmed in our phylogenetic analyses (Fig. 2). In concordance with this taxonomic revision, we propose to synonymize *Stanjemonium spectabile* with *Trichothecium*, as the latter is the more ancient generic epithet, thereby transferring *S. spectabile* to the genus *Trichothecium* and treat it as *Trichothecium spectabile*.

Several studies have previously validated the use of an OSMAC approach to expand the chemical space of a strain, highlighting the impact that fermentation parameters have on the expression of biosynthetic genes (Bode et al. 2002; Bills et al. 2008). In this work, the study of the nutritional requirements of the strain through the use of FFBiolog microplates allowed us to explore the preferences of the strains in terms of carbon and nitrogen sources, generating the necessary information to tailor a panel of fermentation media designed for the OSMAC-based media study. It is known that efficient and consistent dereplication methods, combined with the use of powerful tools and broad databases such as GNPS, the DNP, and NPAtlas, facilitate the identification of known and unknown molecules (Wang et al. 2016; Pérez-Victoria 2024). Based on this, we investigated the chemical diversity generated by the OSMAC-based experiment.

For instance, the submerged liquid conditions containing high amount of sugars such YES (sucrose), YEC (cellobiose), XFUGA (xylose and fructose), WS80 (xylose and fructose) and M104T(sorbitol and glucose) triggered the production of several annotated putative compounds like trichothecenes and other mycotoxins, where solid state fermentation conditions containing rice or wheat grains (BRFT and Wheat-1) stimulated the development of aerial mycelia while inhibiting the production of most toxins (Suppl. material 1: table S2). It is known that media formulations containing a considerable amount of carbon and nitrogen sources support fungal growth at the expense of sporulation and, as a result, reduce the production of mycotoxins (Calvo et al. 2002; Brodhagen and Keller 2006; Tribelhorn et al. 2023). In this study we also observed that the addition of SAHA can inhibit the production of secondary metabolites, as seen for several components and toxins on the CYS80 and SXSY media, or enhance it, as shown by the production of verrucarin J and trichodepsipeptide B on the FOF medium. It has been reported that the perturbation of the normal state of chromatin in filamentous fungi can interfere with its regulatory functions, thus sometimes leading to the activation of cryptic regions of the chromosome (Brosch et al. 2008). Epigenetic modifiers like the DNMT inhibitor hydralazine hydrochloride or the HDAC inhibitor SAHA have been successfully used to modulate the chromatin dynamics and stimulate the expression of silent genes (Scherlach and Hertweck 2009; Cichewicz 2010; González-Menéndez et al. 2016).

As reported in the results, the use of culture media tailored to the nutritional preferences of the strain expanded its chemical space, leading to the identification of several unique compounds not detected in other cultivation conditions (Suppl. material 1: table S2); for example, FCRR toxin and kadhenrischinin E are only produced when sucrose is replaced by cellobiose as a primary carbon source in YEC medium compared to YES medium. The production of unique compounds in culture media containing cellobiose has already been observed in previous studies as in the case of the antibiotic TKR2999, only produced in CMK medium (Crespo et al. 2020), emphasising the importance of knowing beforehand the substrate preferences of the strain under study.

Chemical dereplication allowed us to identify putative compounds that were previously found by Krohn et al. (2003) in the fermentation extracts of *A. spectabile NBRC 101148*, such as trichodermol, 8-α-acetoxyverrol, verrol, and the ubiquitous chemotypic compound verrucarin L-acetate (Suppl. material 1: fig. S7). We also identified roridin A, trichoverrol A and verrucarins (Suppl. material 1: fig. S7) which, along with 8-α-acetoxyverrol and trichodermol belong to the family of trichothecenes, a group of fungal toxins produced by species of the genera *Fusarium*, *Myrothecium*, *Spicellum*, *Stachybotrys*, *Cephalosporium*, *Trichoderma*, and *Trichothecium* (Richardson et al. 1989; McCormick et al. 2011). Trichodepsipeptide B and guangomide B (Suppl. material 1: fig. S7) are instead characteristic compounds of the *Trichothecium* genus (Springer et al. 1984; Sy-Cordero et al. 2011). The presence of all these chemotaxonomic biomarkers further supports our reclassification proposal and, additionally, other members of the *Stanjemonium* genus, like *Stanjemonium grisellum*, are known to produce peptaibols (Sánchez-Giraldo 2024), a group of polypeptides with antimicrobial activity, typically produced by *Trichoderma* and *Emericellopsis* genera (Molnár et al. 2010; http://www.cryst.bbk.ac.uk/peptaibol), which are completely absent in the examined strains of *T. spectabile* CF-278320 and CBS 340.70T.

Combined OSMAC and metabolomic approaches also allowed us to discover the novel cytotoxic compound spectabilide A and its cluster of analogues. Spectabilide A belongs to the group of cyclic lipodepsipeptides, a large family of peptide-derived compounds that show a wide range of biological activities, such as antimicrobial, immunosuppressive, cytotoxic, and antitumoral. They are broadly distributed and mainly produced by bacteria, marine organisms and filamentous fungi. These compounds are usually biosynthesized by the combination of a non-ribosomal peptide synthetase (NRPS) synthetizing the aminoacidic moiety and a polyketide synthase (PKS) involved in the fatty acid synthesis (Bionda et al. 2013; Sivanathan and Scherkenbeck 2014; Kitagaki et al. 2015; Wang et al. 2018; Evidente 2022). The compound has a simple structure, composed of a hydroxylated fatty acid chain linked to a chain of three amino acids: dehydroalanine, ß-alanine, and homoglutamine. This structure resembles that of fusaristatins, a group of metabolites produced by *Fusarium* spp., which also belongs to the *Hypocreales* order (Shiono et al. 2007). Referring to the reported bioactivity of fusaristatins, we tested the cytotoxic activity of spectabilide A against a panel of five human tumour cell lines. The novel compound displayed potent cytotoxicity, with the strongest activity (ED_50_ = 0.11 µM, 95% CI 0.11–0.11 µM) against the MCF-7 breast adenocarcinoma cell line. For instance, the anti-tumour effects reported in literature for fusaristatins A and B against LU 65 lung cancers cells were much weaker, with IC_50_ values of 23 µM and 7 µM, respectively (Shiono et al. 2007).

## Conclusions

In this study, we proposed the new combination of *Trichothecium spectabile* comb. nov. ((W. Gams) Pecin & Gonzalez-Menéndez) based on phylogenetic and chemotaxonomic evidence. The chemical diversity of *T. spectabile* has been expanded through the combined use of OSMAC and metabolomic approaches, allowing to enrich its chemical space with 29 molecules that are poorly produced or not produced at all under standard cultivation conditions in most of the cases. The extensive analysis we carried out on the fermentation extracts of the strains also revealed the presence of a novel cyclic depsipeptide, spectabilide A, exhibiting a potent cytotoxic activity against a panel of tumoral cell lines, suggesting a potential pharmacological interest for the molecule. The production of spectabilide A by *T. spectabile* highlights the ability of plant associated fungi to produce novel molecules of interest with different biological activities, and of high relevance for the field of drug discovery. Further studies are needed to define the biosynthetic process behind the production of the novel cyclodepsipeptide, as well as to explore the unexpressed biosynthetic potential of *T. spectabile* strains. Nevertheless, this study has highlighted the value of using a combined approach involving metabolomics, phylogeny and chemotaxonomy to uncover both new insights into the reclassification of fungal species and new compounds with interesting biological activities.

## Supplementary Material

XML Treatment for Trichothecium spectabile

## Additional information

### Artificial Intelligence (AI) use

The authors accept full responsibility for the content of the manuscript, including the disclosure of any use of AI.

No AI tools were used in the preparation of this manuscript.

### Adherence to national and international regulations

All the fungal strains used in this study have been legally obtained, respecting the Convention on Biological Diversity (Rio Convention).

### Author ORCIDs

G. Pecin https://orcid.org/0009-0009-5693-0200

V. Gonzalez-Menendez https://orcid.org/0000-0001-9859-6284

J. Martin https://orcid.org/0000-0001-7487-2790

F. Annang https://orcid.org/0000-0002-3014-2750

T.A. Mackenzie https://orcid.org/0009-0000-4297-6966

F. Reyes https://orcid.org/0000-0003-1607-5106

O. Genilloud https://orcid.org/0000-0002-4202-1219

### Data availability

All of the data that support the findings of this study are available in the main text or Supplementary Information.

## Supplementary materials

Supplementary material 1 Additional figures and tables Data type docx https://binary.pensoft.net/file/1694369This dataset is made available under the Open Database License (http://opendatacommons.org/licenses/odbl/1.0/). The Open Database License (ODbL) is a license agreement intended to allow users to freely share, modify, and use this Dataset while maintaining this same freedom for others, provided that the original source and author(s) are credited. Gioele Pecin
