# Supplementary material for: Discovery of spectabilide A, a new cytotoxic cyclic lipodepsipeptide from Trichothecium spectabile comb. nov. (Ascomycota, Sordariomycetes) revealed by OSMAC-guided metabolomics
Source: IMA Fungus. 2026 Jun 24;17:e192814. doi: 10.3897/imafungus.17.192814 (PMC13383978; doi:10.3897/imafungus.17.192814)
Supplement: Supplementary material 1 — Additional figures and tables [file imafungus-17-e192814-s001.docx]

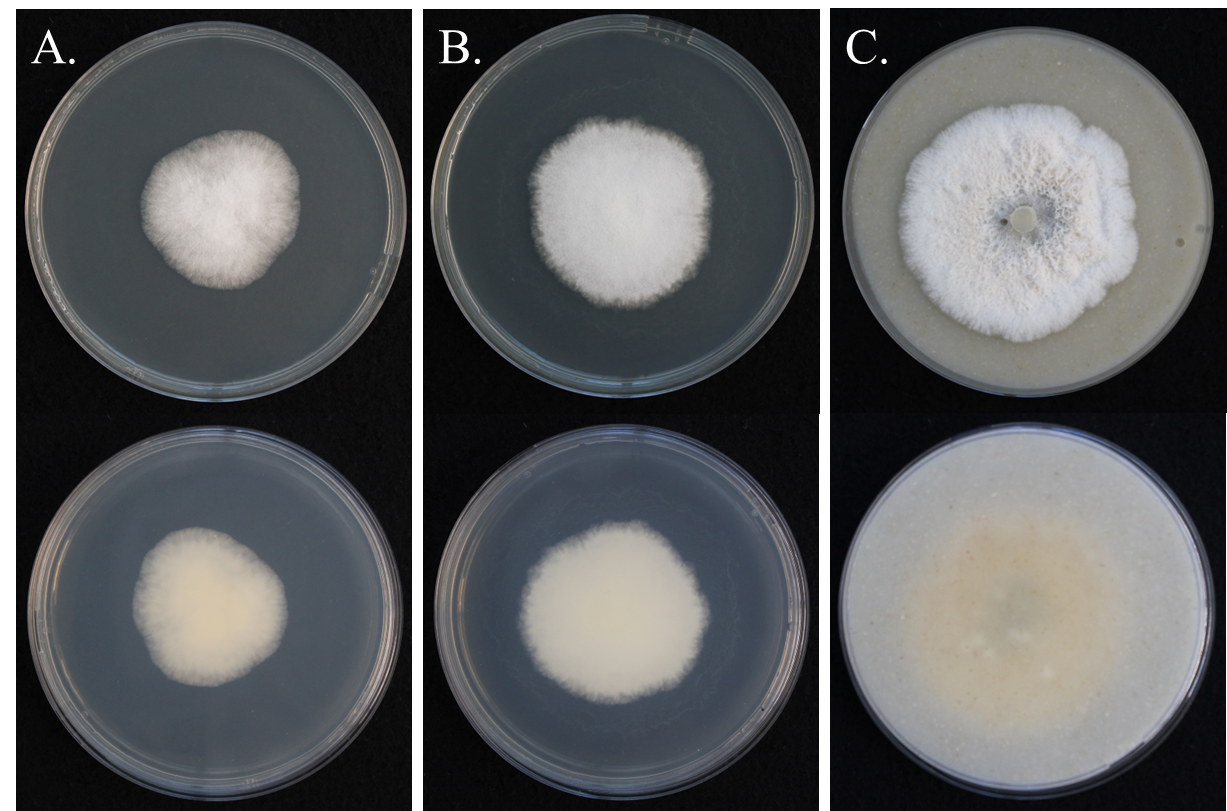


Figure S.1 T. spectabile CF-278320 colonies on YME (**A**), PDA (**B**) and OA (**C**) plates at 12 days cultivation. Colony size: 3.8 cm on YME (**A**); 4.4 cm on PDA (**B**); 5.38 cm on OA (**C**).


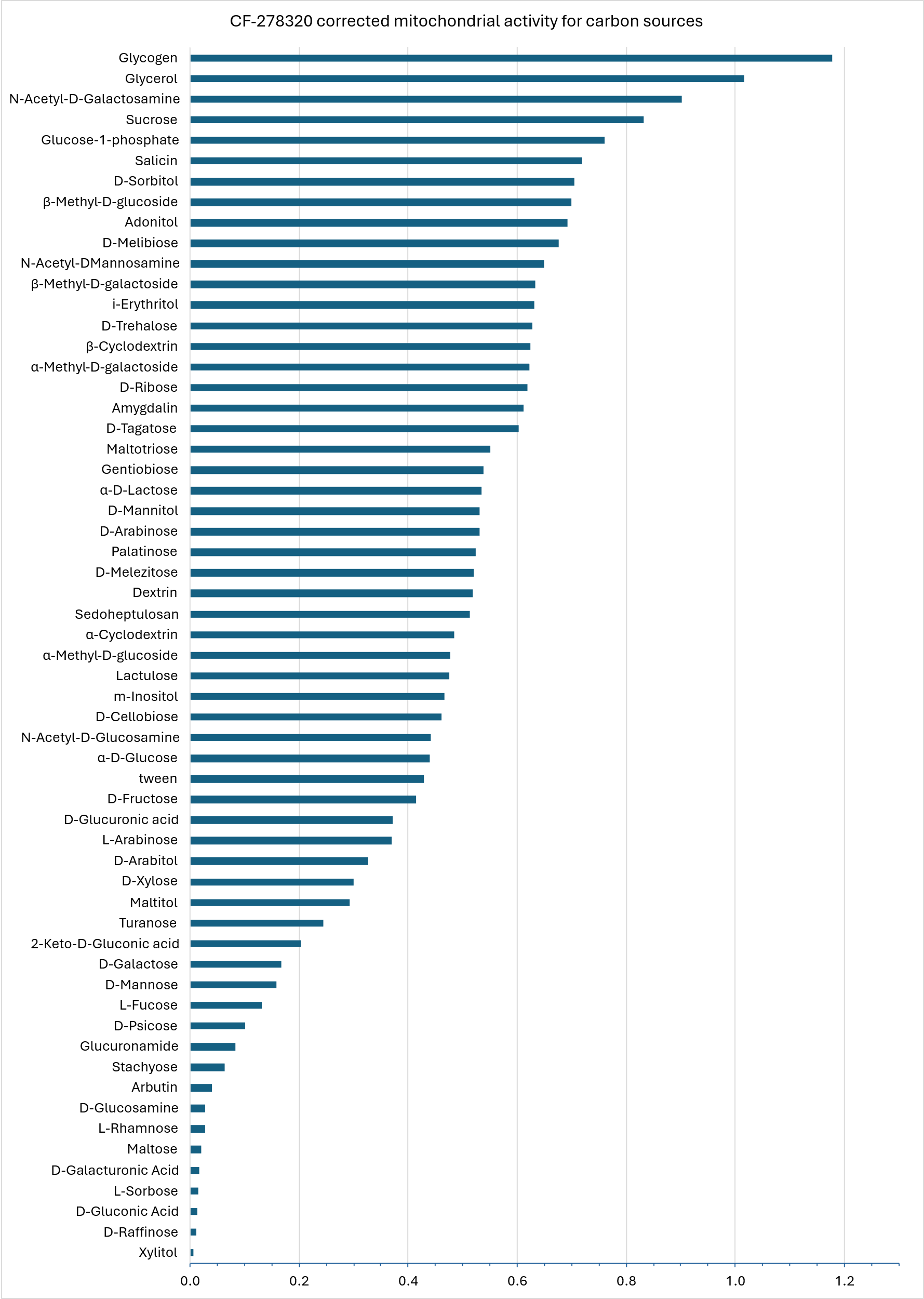


Figure S.2 FFBiolog microplates corrected mitochondrial activities measured for C sources for S. spectabile strain CF-278320.


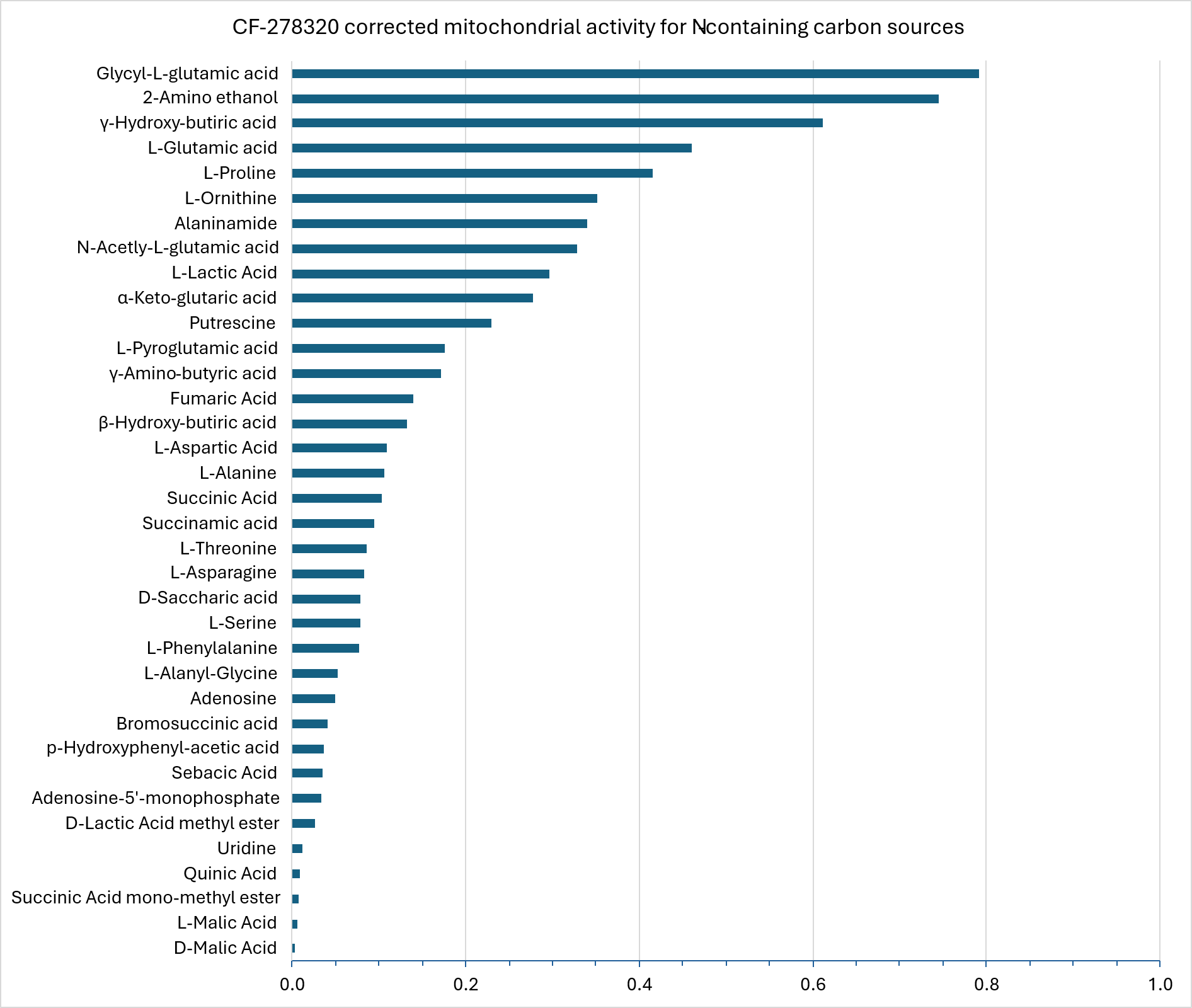


Figure S.3 FFBiolog microplates corrected mitochondrial activities measured for N-containing sources for S. spectabile strain CF-278320.


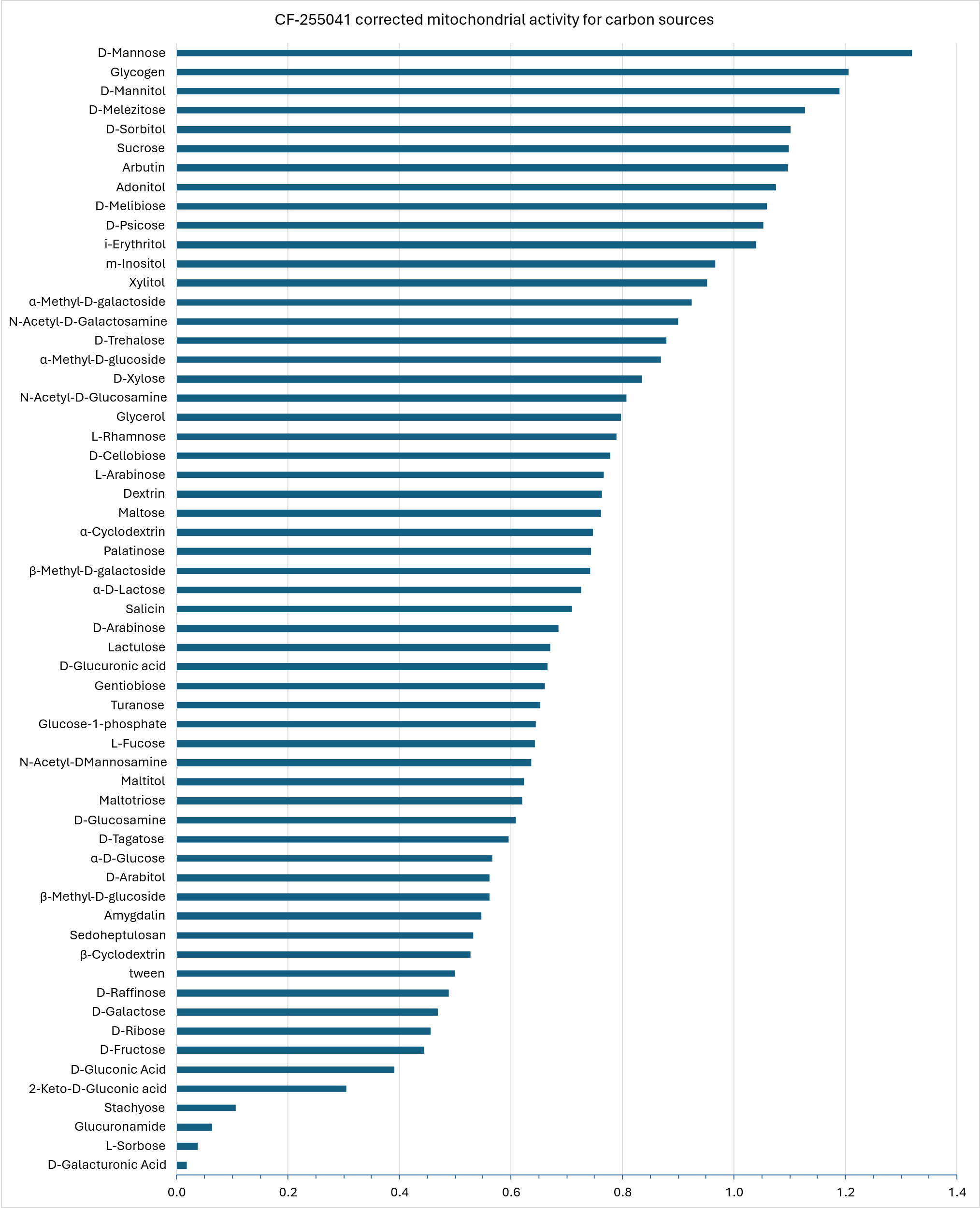


Figure S.4 FFBiolog microplates corrected mitochondrial activities measured for C sources for S. spectabile strain CBS 340.70.


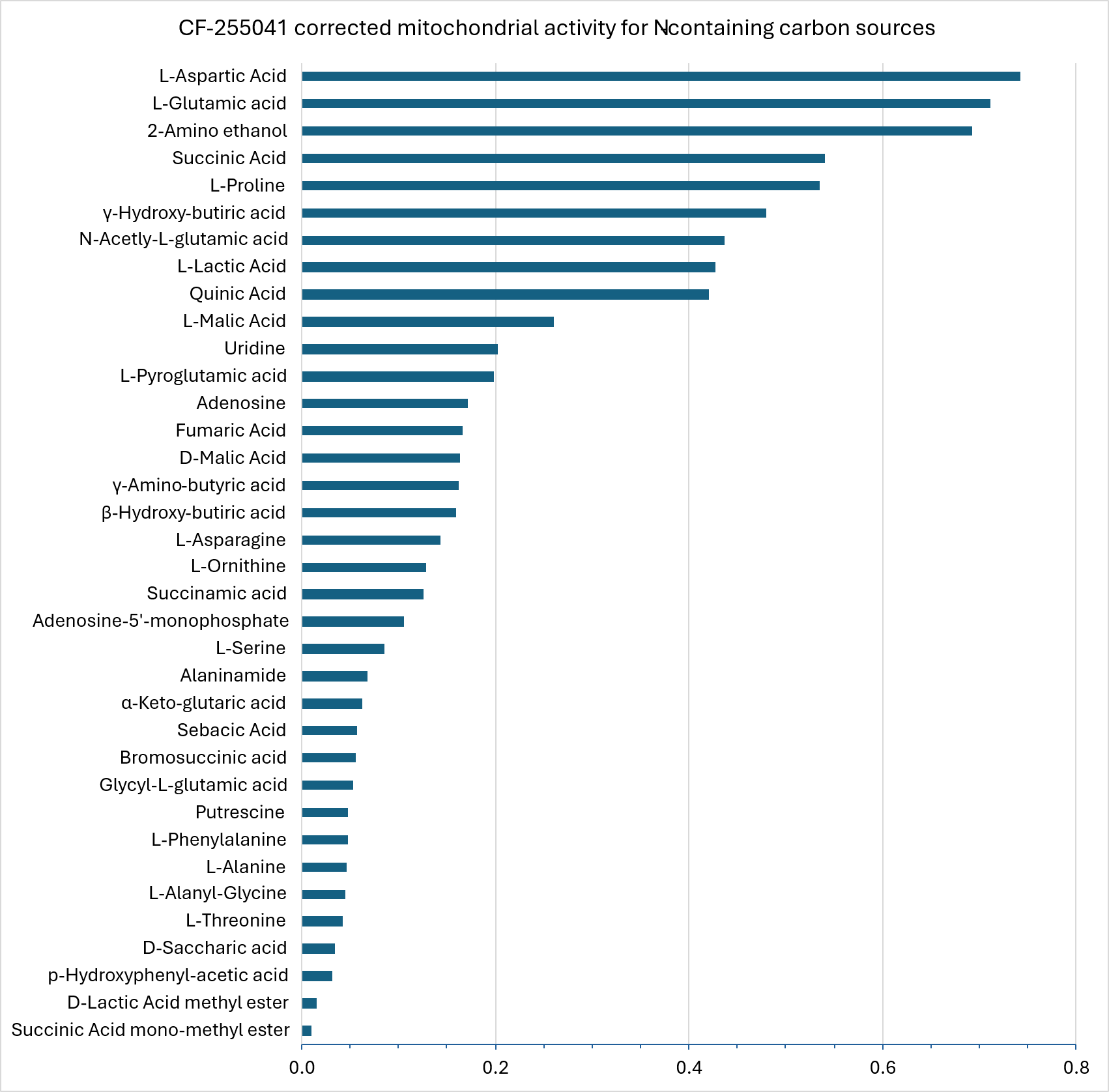


Figure S.5 FFBiolog microplates corrected mitochondrial activities measured for N-containing sources for S. spectabile strain CBS 340.70.

Table S.1 Culture media composition.

| **Medium** | **Components (per liter)** | **Reference** |
| --- | --- | --- |
| BRFT | Brown rice (200g), base liquid (400mL), Bacto™ Yeast Extract (10g), sodium tartrate (5g), KH_2_PO_4_ (5g) | Peláez et al., 2000 |
| CYS80  (+ SAHA) | Sucrose (80g), yellow corn meal (50g), Bacto™ Yeast Extract (1g) | Suay et al., 2000 |
| DEGSY | Dextrin from potato starch (40g), L-glutamic acid (38g), soybean flour (1g), Bacto™ Yeast Extract (5g) | This study |
| Dex-Soy  (+ XAD-16) | Dextrin (40g), glucose (10g), malt extract (5g), ploypeptone (5g), soybean flour (5g), Bacto™ Yeast Extract (2g), KH_2_PO_4_ (1g) | Georgousaki et al., 2019 |
| FGY-2  (+ XAD-16) | Fructose (40g), monosodium glutamate (8g), Bacto™ Yeast Extract (8g), L-glutamic acid (15g), KH_2_PO_4_ (1.5g), MgSO_4_•7H_2_O (0.4g), trace elements (10mL: FeSO_4_•7H_2_O 1g/L, MnSO_4_•H_2_O 1 g/L, ZnSO_4_•7H_2_O 0.4 g/L, CaCl_2_•2H_2_O 0.1 g/L, HBO_3_ 0.056 g/L, CuCl_2_•2H_2_O 0.025 g/L, (NH_4_)_6_Mo_7_O_24_•4H_2_O 0.019 g/L, HCl (12 N) 50 mL/L) | This study |
| FOF  (+ SAHA) | Fructose (75g), oat flour (15g), Bacto™ Yeast Extract (5g), L-glutamic acid (4g), MES (16.2g), adjusted to pH 6.0 | This study |
| M104T | Sorbitol (100g), glucose (40g), glutamic acid (10g), KH_2_PO_4_ (0.5g), MgSO_4_ (0.5g), Bacto™ Yeast Extract (3g), DL-tryptophan (0.8g) | Bacon, 1988 |
| MV8  (+ SAHA) | Maltose (75g), V-8 juice (200mL), soy flour (1g), L-proline (3g), MES (16.2g) | González-Menéndez et al., 2014 |
| SM  (+ XAD-16) | D-sorbitol (100g), glucose (40g), succinic acid (10g), KH_2_PO_4_ (1g), MgSO_4_•7H_2_O (0.3g), Bacto™ Yeast Extract (1g), trace elements (5mL: citric acid 0.5 g/L, Fe(NH_4_)_2_(SO_4_)_2_•5H_2_O 0.1 g/L, ZnSO_4_•7H_2_O 0.5 g/L, MnSO_4_•H_2_O 0.005 g/L, Na_2_MoO_4_•2H_2_O 0.005 g/L, CuSO_4_•5H_2_O 0.025 g/L), adjusted to pH 5.6 | This study |
| SXSY  (+ SAHA) | Soluble potato starch (40g), xylose (40g), L-serine (25g), Bacto™ Yeast Extract (5g) | This study |
| Wheat-1 | Wheat grain (500g), liquid base (220mL), glycerol (2g), Bacto™ Yeast Extract (2g), sodium tartrate (10g), KH_2_PO_4_ (1g), MgSO_4_•7H_2_O (1g), FeSO_4_•7H_2_O (0.05g) | Bills et al., 2008 |
| WS80  (+ SAHA) | Wheat flour (50g), xylose (40g), fructose (40g) | Ondeyka et al., 2007 |
| XYFUGA  (+ XAD-16) | Xylose (40g), fructose (40g), N-acetylglucosamine (5g), L-aspartic acid (2g) | This study |
| YEC  (+ XAD-16) | Bacto™ Yeast Extract (20g), D-cellobiose (150g), MgSO_4_•7H_2_O (0.5g), YES trace elements (1mL) | This study |
| YES  (+ XAD-16) | Bacto™ Yeast Extract (20g), MgSO_4_•7H_2_O (0.5g), sucrose (150g) trace elements (1mL) | González-Menéndez et al., 2014 |


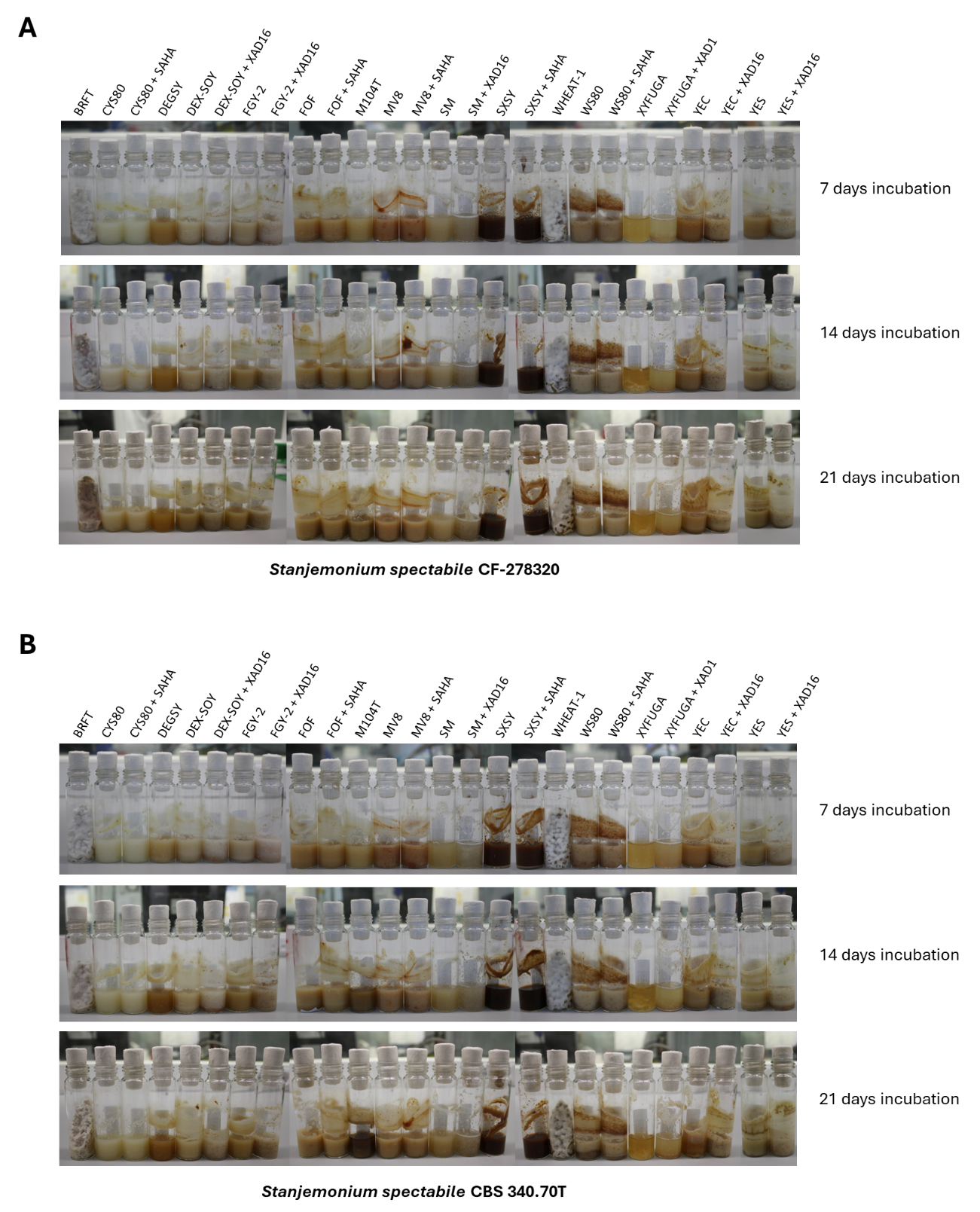


Figure S.6 Media testing cultivation in EPA vials format of **A.** S. spectabile CF-278320 and **B.** S. spectabile CBS 340.70T.

Table S.2. List of the annotated putative compounds and their corresponding production media/conditions, ordered by the number of media in which they were found.

Figure S.7 Structures of the most abundant putative compounds produced by T. spectabile.


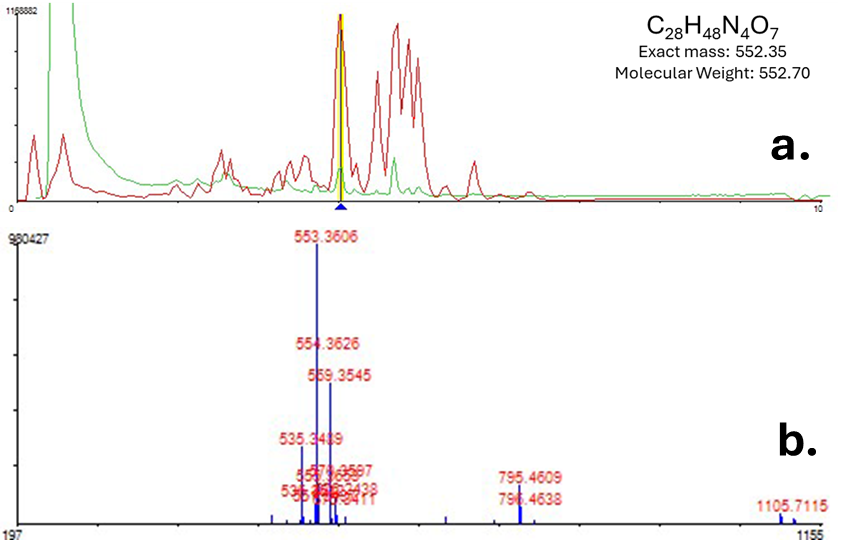


Figure S.8 **A** UV-LCMS chromatogram obtained the analysis of extracts from BRFT crude extract of S. spectabile. **B** Extracted MS spectrum in positive mode (m/z 552.35, C_28_H_48_N_4_O_7_).


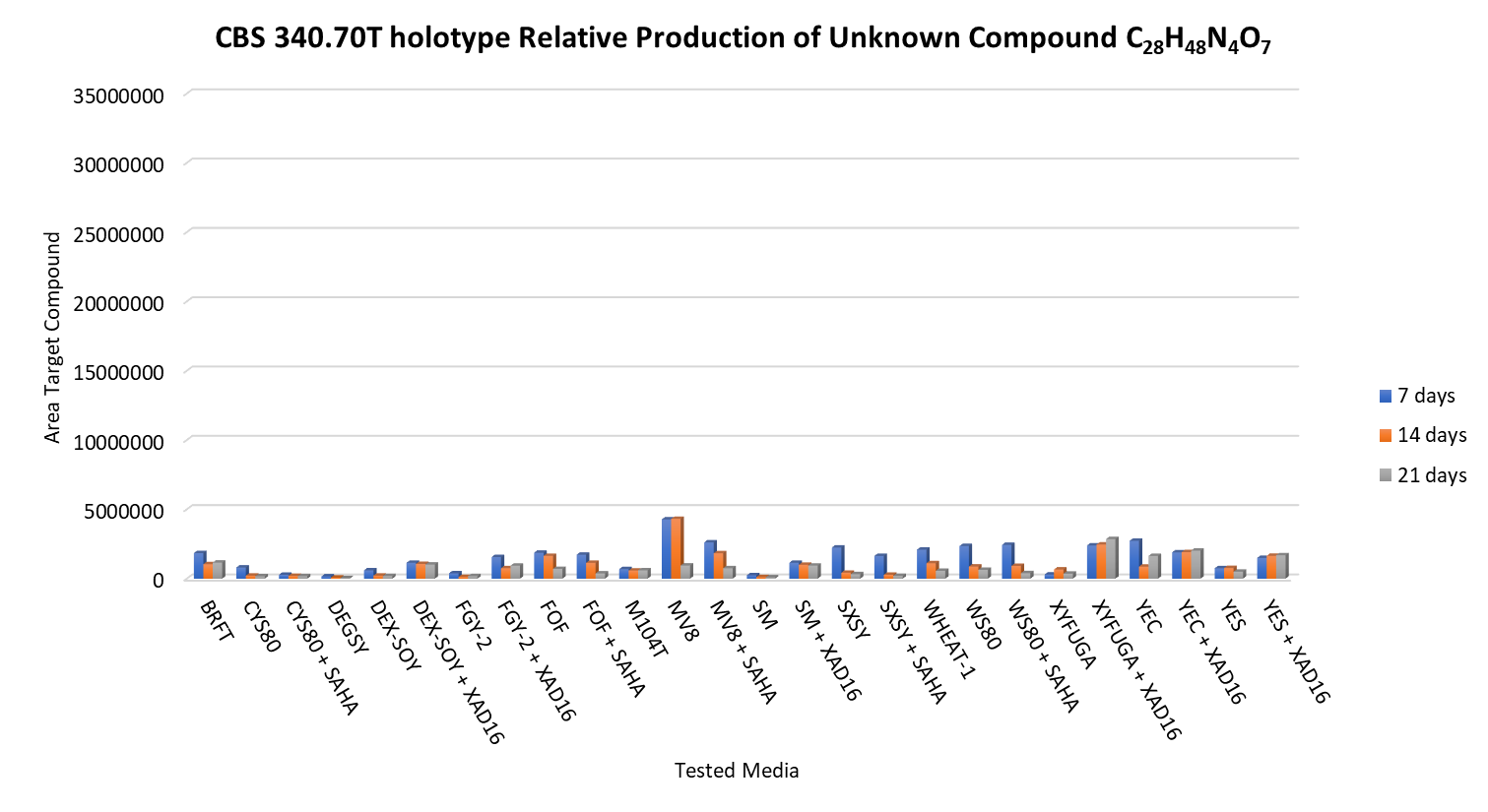


Figure S.9 Relative production of compound C_28_H_48_N_4_O_7_ for S. spectabile CBS 340.70T on the different media, represented as area under the peak of the target compound (EIC 553.360 ± 0.005)


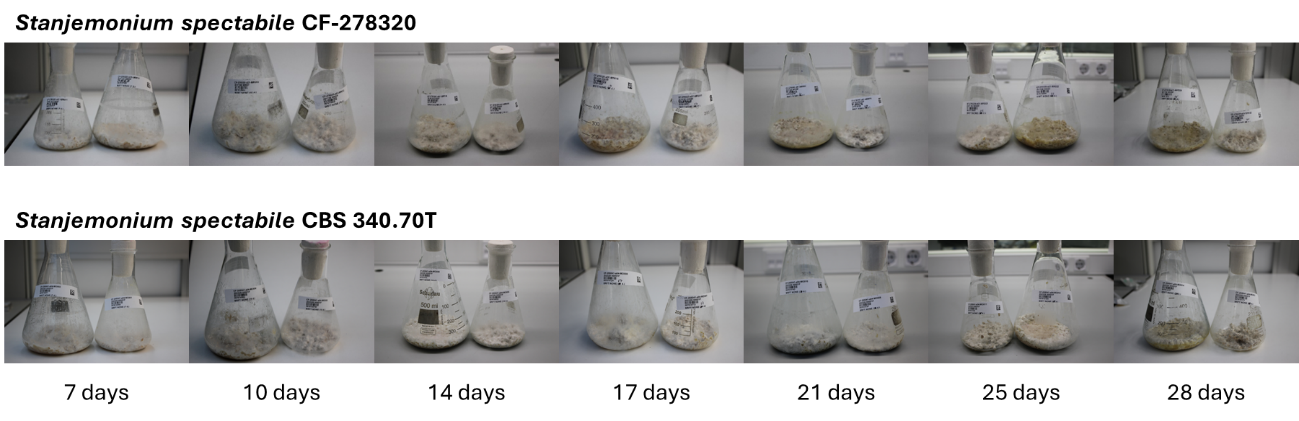


Figure S.10 Time course cultivation of S. spectabile strains CF-278320 and CBS 340.70T in BRFT medium.


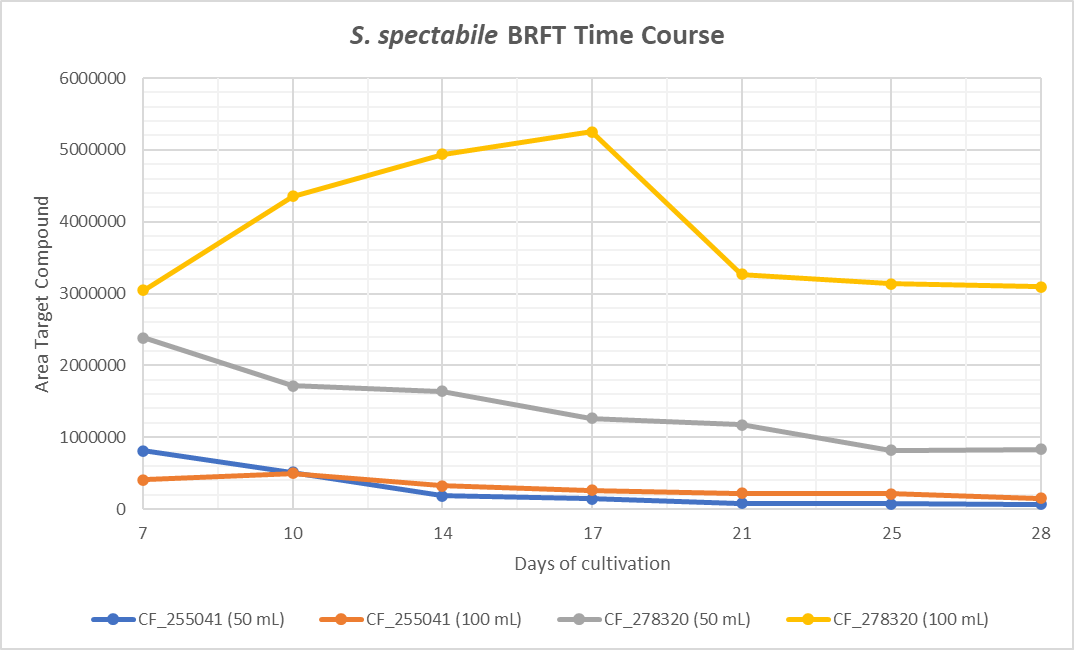


Figure S.11 Relative production of the unknown target compound during the time course experiment measured as the area under the peak of the UHPLC-UV chromatogram at 210 nm compared to the control areas.


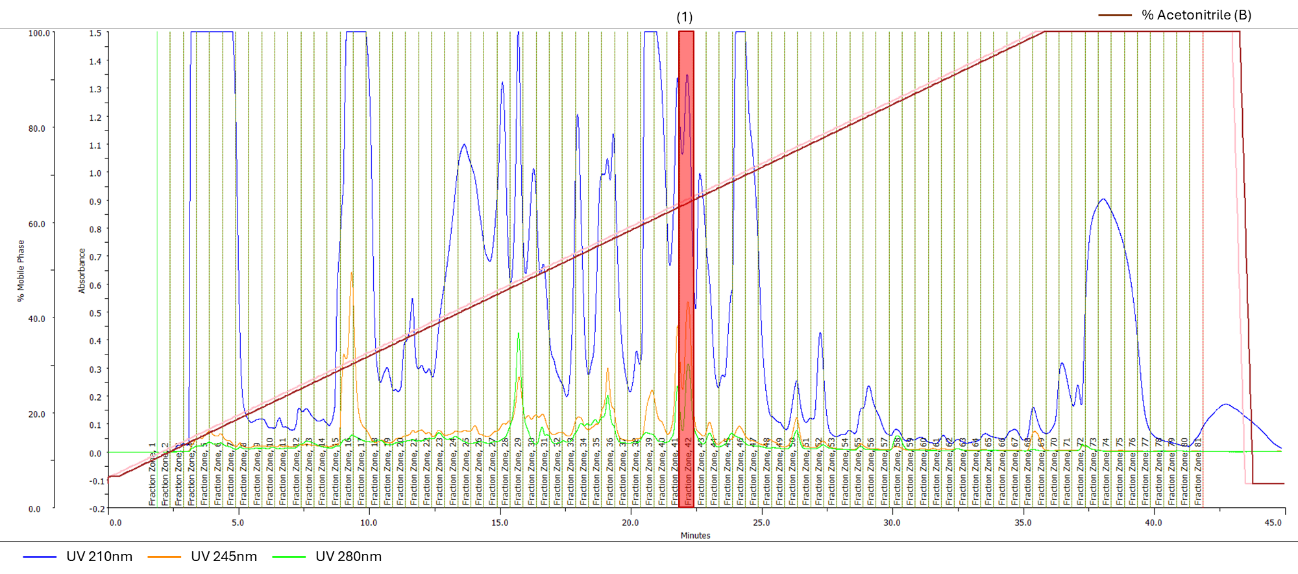


Figure S.12 Chromatogram of the first purification round of compound C_28_H_48_N_4_O_7_ (1) by semi-preparative reverse phase HPLC. The mobile phase was constituted by water + 0.1 % TFA (A) and acetonitrile +0.1 % TFA (B). UV detection was set at 210 nm (blue trace), at 245 nm (orange trace) and at 280 nm (green trace).


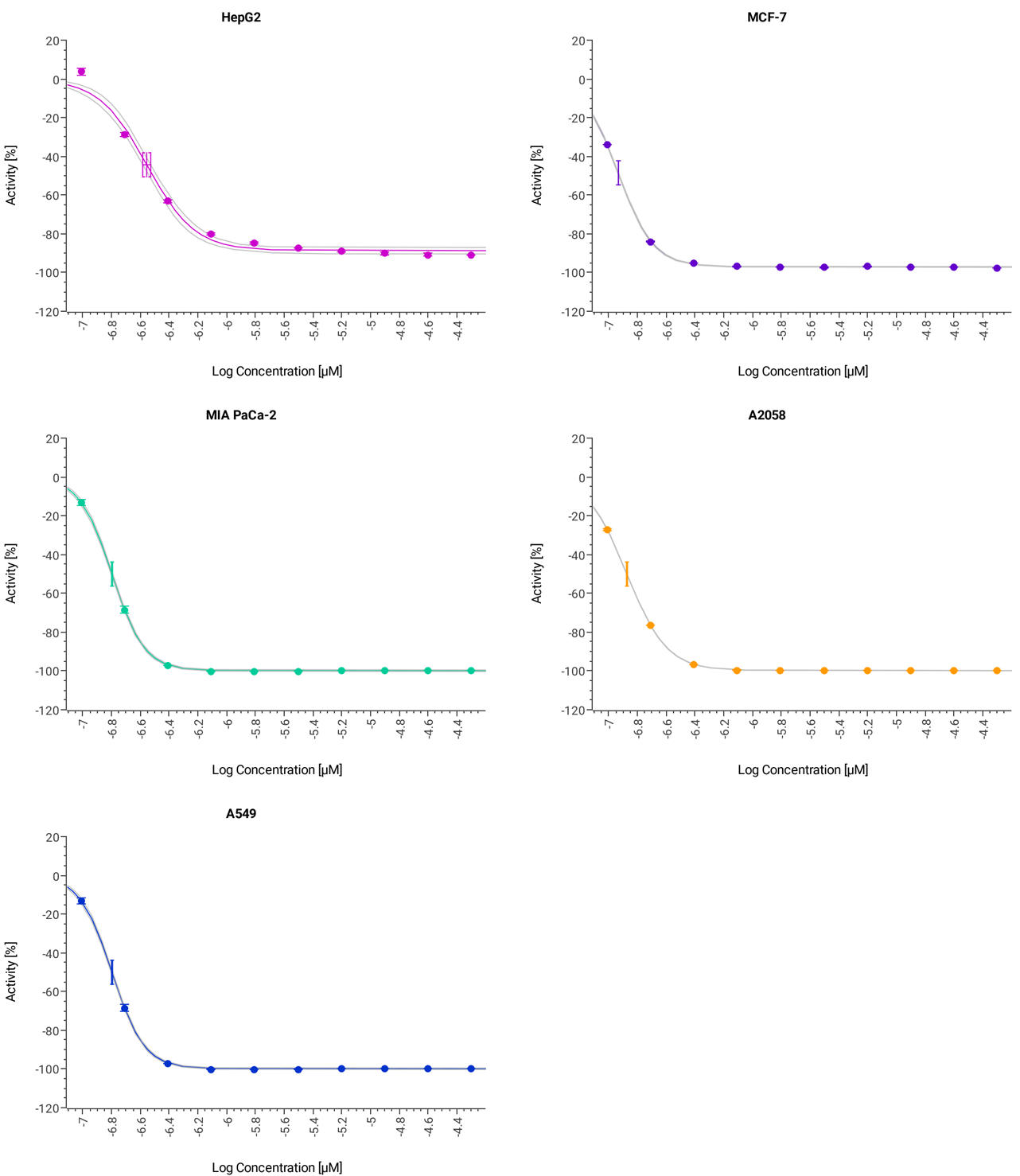


Figure S.13 Cytotoxicity MTT reduction assay dose-response curves for the panel of different tumoral cell lines.

Figure S.14 ^1^H NMR spectra of spectabilide A (500 MHz, methanol-d_4_).

Figure S.15 ^13^C NMR spectra of spectabilide A (125 MHz, methanol-d_4_).

Figure S.16 ^1^H-^13^C HMBC NMR spectra of spectabilide A (500 MHz, 125 MHz, methanol-d_4_).

Figure S.17 ^1^H-^1^H NOESY NMR spectra of spectabilide A (500 MHz, methanol-d_4_).

Figure S.18 ^1^H-^13^C HSQC NMR spectra of spectabilide A (500 MHz, 125 MHz, methanol-d_4_).

Figure S.19 ^1^H-^1^H COSY NMR spectra of spectabilide A (500 MHz, methanol-d_4_).

Figure S.20 ^1^H-^1^H TOCSY NMR spectra of spectabilide A (500 MHz, methanol-d_4_).


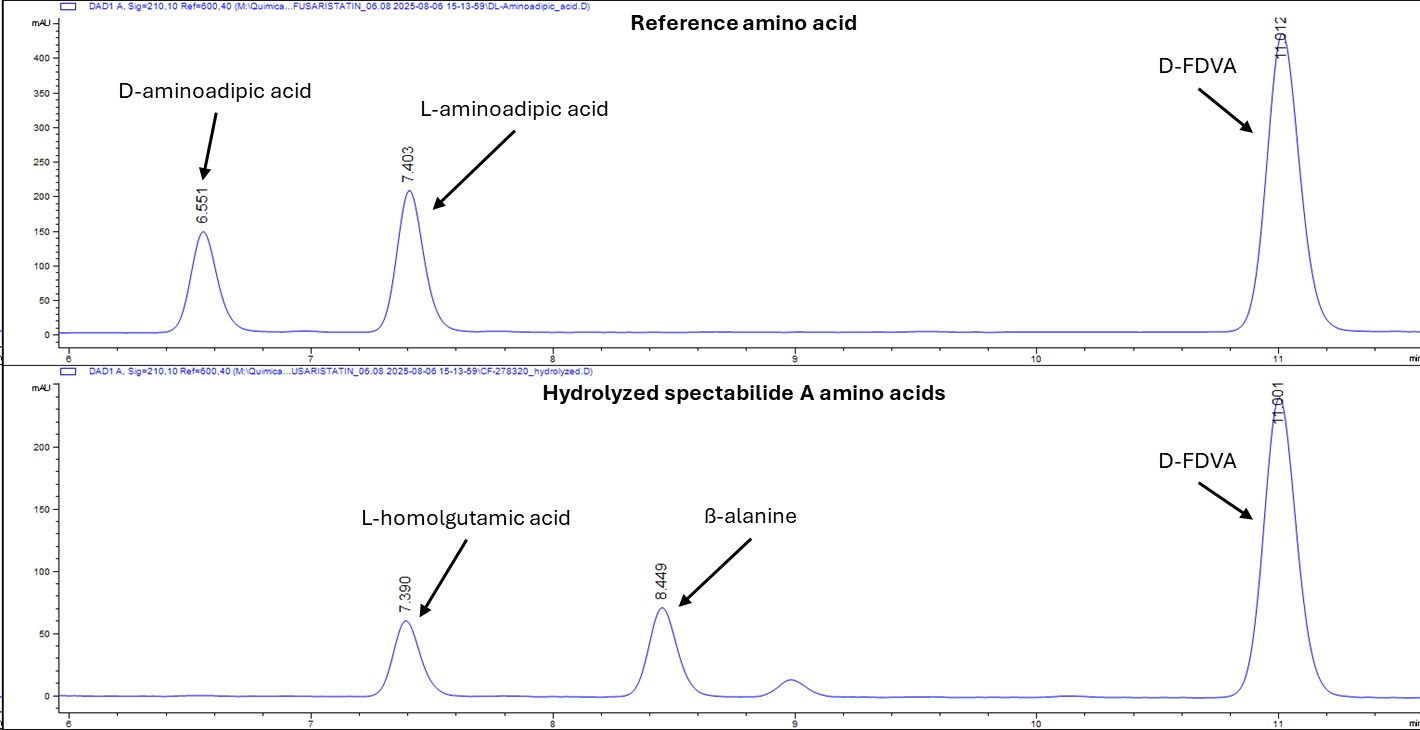


Figure S.21 Chromatographic profiles of the standard amino acid and of spectabilide A amino acids.

Table S.3 List of selected strains used in the ITS phylogenetic study, with their accession numbers.

| **Taxa** | **Strain** | **ITS GenBank accession number** | **References** |
| --- | --- | --- | --- |
| *Acremonium brachypenium* | CBS 866.73 | AB540570 | Kiyuna (2011) |
| *Acremonium fuci* | 9151P3 | JQ796750 | Konovalova (2011) |
| *Acremonium fusidioides* | UTHSC 08-1188 | FN706543 | Perdomo (2011) |
| *Acremonium fusidioides* | CBS 840.68 | FN706542 | Perdomo (2011) |
| *Acremonium hansfordii* | CBS 390.73 | AB540578 | Kiyuna (2011) |
| *Acremonium kiliense* | MUCL 9724 | FN691446 | Perdomo (2011) |
| *Acremonium murorum* | JZ-151 | HQ637278 | Unpublished |
| *Acremonium persicinum* | A20 | JQ012760 | Lo Piccolo (2015) |
| *Acremonium persicinum* | CBS 310.59 | FN706554 | Perdomo (2011) |
| *Acremonium persicinum* | UTHSC 01-1389 | FN706546 | Perdomo (2011) |
| *Acremonium persicinum* | UTHSC 01-1249 | FN706545 | Perdomo (2011) |
| *Acremonium polychromum* | NBRC 9440 | AB540566 | Kiyuna (2011) |
| *Acremonium polychromum* | CBS 181.27 | AB540567 | Kiyuna (2011) |
| *Acremonium potronii* | MS02 | JN021531 | Mouton (2019) |
| *Acremonium sclerotigenum* | CBS 281.80 | FN706549 | Perdomo (2011) |
| *Acremonium sclerotigenum* | CBS 124.42 | FN706552 | Perdomo (2011) |
| *Acremonium sclerotigenum* | CBS 270.86 | FN706551 | Perdomo (2011) |
| *Acremonium strictum* | GQ450275 | GU595023 | Xing (2010) |
| *Acremonium strictum* | CBS 346.70 | GQ376096 | Wicht (2012) |
| *Acremonium zeae* | NRRL 45893 | HM751086 | Poling (2008) |
| *Acremonium zeae* | CBS 226.84 | EU887241 | Wicklow (2009) |
| *Aphanocladium spectabile* | NBRC 101148 | - | Gams (1998) |
| *Bionectria compactiuscula* | CBS 592.93 | AF358247 | Cannon (2003) |
| *Bionectria grammicosporopsis* | CBS 111.87 | AF358255 | Cannon (2003) |
| *Bionectria pseudostriata* | CBS 119.87 | AF358251 | Cannon (2003) |
| *Bionectria ralfsii* | CBS 102845 | AF358253 | Cannon (2003) |
| *Bionectria samuelsii* | CBS 700.97 | AF210689 | Cannon (2003) |
| *Bionectria solani* | CBS 702.97 | AF210687 | Cannon (2003) |
| *Bionectria zelandiaenovae* | CBS 100979 | AF358229 | Cannon (2003) |
| *Ceratocystis fimbriata* | CMW2220; PREM51644 | AF043604 | Witthuhn (1999) |
| *Emericellopsis glabra* | CBS 119.40 | AY632657 | Zuccaro (2004) |
| *Emericellopsis minima* | CBS 190.55 | AY632669 | Zuccaro (2004) |
| *Emericellopsis terricola* | NBRC 7898 | AB425992 | Duc (2009) |
| *Endophyte* | IB211 | JX156018 | Gazis (2015) |
| *Gliomastix masseei* | CBS 794.69 | AB540553 | Kiyuna (2011) |
| *Gliomastix murorum* | NBRC 31241 | AB540557 | Kiyuna (2011) |
| *Hypocreales sp.* | KLAR B5 | EU164804 | Chinworrungsee (2008) |
| *Myrotheciomyces corymbiae* | CPC 33206 | NR_160351 | Crous (2018) |
| *Sarocladium kiliense* | CanL-10b | JF817256 | Zhang (2014) |
| *Stanjemonium dichromosporum* | CBS 638.73 | NR_077122 | Schoch (2014) |
| *Stanjemonium fuscescens* | CBS 264.96 | NR_160198 | Vu (2019) |
| *Stanjemonium grisellum* | CBS 100389 | PV272738 | Zhao (2025) |
| *Stanjemonium grisellum* | CBS 655.79 | NR_156501 | Zuccaro (2004) |
| *Stanjemonium ochroroseum* | CBS 656.79 | NR_137145 | Zuccaro (2004) |
| *Stanjemonium spectabile* | CBS 340.7^T^ | MH859702 | Vu (2019) |
| *Stanjemonium spectabile* | CF-278320 | PX069749 | Present study |
| *Stilbella albocitrina* | CBS 526.84 | - | Present study |
| *Trichothecium crotocinigenum* | cc56 | DQ882846 | Evans (2006) |
| *Trichothecium crotocinigenum* | Dzf16 | EU543259 | Zhang (2008) |
| *Trichothecium hongkongense* | CBS 117586 | OQ429888 | Hou (2023) |
| *Trichothecium hongkongense* | CBS 101444 | OQ429887 | Hou (2023) |
| *Trichothecium hongkongense* | CBS 102186 | OQ429886 | Hou (2023) |
| *Trichothecium indicum* | CBS 123.78 | OQ429889 | Hou (2023) |
| *Trichothecium ovalisporum* | MB 505966 | EU445372 | Seifert (2008) |
| *Trichothecium roseum* | LCP 50.627 | JQ434580 | Ropars (2012) |
| *Trichothecium roseum* | CBS 113334 | EU552162 | Marincowitz (2008) |
| *Trichothecium sp* | CF-136858 | - | Present study |
| *Trichothecium sp.* | CBS 254.77 | OQ429890 | Hou (2023) |
| *Trichothecium sp.* | HF8 | GU183172 | Unpublished |
| *Trichothecium sympodiale* | JCM 10014 | AB019365 | Okada (2022) |

Table S.4 Alignment used in the ITS phylogenetic study.

>Ceratocystis_fimbriata_AF043604 internal transcribed spacer 1, 5.8S ribosomal RNA gene and internal transcribed spacer 2, complete sequence; and large subunit ribosomal RNA gene, partial sequence...

ACT-GAGTTTTTGTAC-------TCTAT-AAACCA-TGTGTGAAC--GTACC--TATCTT

GTAGTGAGATGAATGCTGTTTTGGTGGT-AGGGCCC--TTC------TGA-AG--GGCAC

CGCTGCCAGCAGTATAGTCTCGCCACTGTAAACTCTT--ATATTTTCCAGATTTTTT---

--------------CAT---TGCTGAG----TGG--CATAACTAT-------AAAAAA--

----G---TTAAAACTTTCAACAACGGATCTCTTGGCTCTAGCATCGATG-AAGAACGC-

AGCGAAATGCGATAAGTAATGTGAATTGCAGAATTCAGTGAATCATCGAATCTTTGAACG

CACATTGCCCCTGGTAGTATTCTGCCAGGCATGCCTGTCCGAGCGTCATTTCACCACTCA

AGGACTCCTTTGTTCTT----GGCGTT----------GGAGGTCCTGTT----CTC---C

---------------------------CCT-------GA-ACA-----GGCCGCCGAAAT

GTATCGGCTGTTATACTTGCCAACTCCCCTGTGTAGTATAAAATTTCTAATTTTTACACT

TTGAAG--TTCTTGTGTAACAC-GCCGCTAAACCAACCCCCTCAACTTTT---G-

>Acremonium_strictum_GU595023, 627 bases, 0 checksum..

ACCAGAGTGCC--CTA---GGC-TCT-CC-AACCC-ATTGTGAAC--ATACC--TATC--

GTTCCCTCGGCGGG------CTCA-------GCGC----GCG----------GT-GCCTC

CGGG-CTCC------GGGCGTCCGCCGGGG-ACAA-C--CAAACTCT--G-ATTT-----

-------TATTGTGAA---TCTCTGAG----GGG--CGAAA-GCCCG---AAAACAAAA-

---TGAA-TCAAAACTTTCAACAACGGATCTCTTGGCTCTGGCATCGATG-AAGAACGC-

AGCGAAATGCGATAAGTAATGTGAATTGCAGAATTCAGTGAATCATCGAATCTTTGAACG

CACATTGCGCCCGCCGGCACTCCGGCGGGCATGCCTGTCCGAGCGTCATTTCAACCCTCA

GGA-CCCC--CTTTC------GGGGG-----------GGACCTGGTGCTGGGGATCAG-C

------------------------GG-CCTCC-------GGGCCC-CTGTCCCC-CAAAT

TGAGTGGCGGTCGCGCCGCAGCC-TCCCCTGCGTAGTAGC-----ACA--CCTC--GCAC

C-GGAG--AGCGGCTCGGCCAC-GCCGTGAAACCCCC-A-A-TTTTTT--AA-GG

>Acremonium_strictum_GQ376096_CBS34670, 627 bases, 0 checksum..

ACCAGAGTGCC--CTA---GGC-TCT-CC-AACCC-ATTGTGAAC--TTACC--AAAC--

GTTCCCTCGGCGGG------CTCA-------GCGC----GCG----------GTGGCCTC

CGGGCCTCC------GGGCGTCCGCCGGGG-AAAA-C--CAAACCCT--G-ATTT-----

-------AAT-CAGTAT-TTCTCTGAG----GGG--CGAAA-GCCCG---AAAACAAAA-

---TGAA-TCAAAACTTTCAACAACGGATCTCTTGGCTCTGGCATCGATG-AAGAACGC-

AGCGAAATGCGATAAGTAATGTGAATTGCAGAATTCAGTGAATCATCGAATCTTTGAACG

CACATTGCGCCCGCCGGCACTCCGGCGGGCATGCCTGTCCGAGCGTCATTTCAACCCTCA

GG--CCCACCCTTCC------GGGGGAG--------CGGGCCTGGTTCTGGGGATCGG-C

------------------------GGCCCTC------G-CGGCCC-CCGTCCCT-CAAAT

TCAGTGGCGGTCGCGCCGCAGCC-TCCCCTGCGTAGTAGC-----ACA-ACCTC--GCAC

C-GGAG--AGCGGAACGACCAC-GCCGTAAAACACCC-A-A-TTTTTT--AA-GG

>Acremonium_kiliense_FN691446_MUCL9724, 627 bases, 0 checksum..

ACCAGAGTGCC--CTA---GGC-TCT-CC-AACCC-ATTGTGAAC--ATACC--TATC--

GTTCCCTCGGCGGG------CTCA-------GCGC----GCG----------GT-GCCTC

CGGG-CTCC------GGGCGTCCGCCGGGG-ACAA-C--CAAACTCT--G-ATTTT----

-------A-TTGTGAA---TCTCTGAG----GGG--CGAAA-GCCCG---AAAACAAAA-

---TGAA-TCAAAACTTTCAACAACGGATCTCTTGGCTCTGGCATCGATG-AAGAACGC-

AGCGAAATGCGATAAGTAATGTGAATTGCAGAATTCAGTGAATCATCGAATCTTTGAACG

CACATTGCGCCCGCCGGCACTCCGGCGGGCATGCCTGTCCGAGCGTCATTTCAACCCTCA

GGA-CCCC--CTTTC------GGGGG-----------GGACCTGGTGCTGGGGATCAG-C

------------------------GG-CCTCC-------GGGCCC-CTGTCCCC-CAAAT

TGAGTGGCGGTCGCGCCGCAGCC-TCCCCTGCGTAGTAGC-----ACA--CCTC--GCAC

C-GGAG--AGCGGCTCGGCCAC-GCCGTGAAACCCCC-A-A-TTTTTT--AA-GG

>Sarocladium_kiliense_JF817256, 627 bases, 0 checksum..

ACCAGAGTGCC--CTA---GGC-TCT-CC-AACCC-ATTGTGAAC--ATACC--TATC--

GTTCCCTCGGCGGG------CTCA-------GCGC----GCG----------GT-GCCTC

CGGG-CTCC------GGGCGTCCGCCGGGG-ACAA-C--CAAACTCT--G-ATCTT----

-------A-TTGTGAA---TCTCTGAG----GGG--CGAAA-GCCCG---ACAACAAAA-

---TGAA-TCAAAACTTTCAACAACGGATCTCTTGGCTCTGGCATCGATG-AAGAACGC-

AGCGAGATGCGATAAGTAATGTGAATTGCAGAATTCAGTGAATCATCGAATCTTTGAACG

CACATTGCGCCCGCCGGCACTCCGGCGGGCATGCCTGTCCGAGCGTCATTTCAACCCTCA

GGA-CCCC--CTTTC------GGGGG-----------GGACCTGGTGCTGGGGATCAG-C

------------------------GG-CCTC-------AGGGCCC-CTGTCCCC-CAAAT

TGAGTGGCGGTCGCGCCGCAGCC-TCCCCTGCGTAGTAGC-----ACA--CCTC--GCAC

C-GGAG--AGCGGCTCGGCCAC-GCCGTGAAACCCCC-A-A-TTTTTT--AA-GG

>Acremonium_zeae_HM751086, 627 bases, 0 checksum..

ACCAGAGTGCC--CTA---GGT-CCT-CC-AACCC-ATTGTGAAC--ATACC--TAAC--

GTTCCCTCGGCGGG------CTCA-------GCGC----GCGG---------GT-GCCTC

CGGG-CTCCG-----GGGCGTCCGCGGGGG-ACAA-G--CAAACCCT--G-ATTTT----

-------A-TCGTGTA---TCTCTGAG----GGG--CGAAA-GCCCG---AAAACCAAA-

---TAAA-TCAAAACTTTTCACAACGGATCTTTTGGCTCTTGGATCGATGTAAGAACGCG

AGCGAAATGCGATAAGGAATGTGAATTGCGGAATTCTGTGAATCATCGAATCTTTGAACG

CACATTGTGCCCGCCGGGACTCTGG--GGCATGCCTTTCCGAGCGTCATTTCCACCCTCA

GGGG-CC---CTTTC------GGGGG-----------GGACCTGGTGCTGGG-ATCAG-C

------------------------GG-CCTC--------GGGCCCCCTTTCCC--CAAAT

ACAGTGGCGGTCGCGCCGCAGCC-TCCCCTGCGTAGTAGC-----ACA--CCTC--GCAC

C-GGAG--AGCGGTTCGACCAC-GCCGTAAAACCCCC-A-ACTTT---CCAA-GG

>Acremonium_zeae_EU887241_CBS22684, 627 bases, 0 checksum..

ACCAGAGTGCC--CTA---GGC-TCT-CC-AACCC-ATTGTGAAC--ATACC--TATC--

GTTCCCTCGGCGGG------CTCA-------GCGC----GCG----------GT-GCCTC

CGGG-CTCC------GGGCGTCCGCCGGGG-ACAA-C--CAAACCCT--G-ATTTT----

-------A-TCGTGTA---TCTCTGAG----GGG--CGAAA-GCCCG---AAAACAAAA-

---TAAA-TCAAAACTTTCAACAACGGATCTCTTGGCTCTGGCATCGATG-AAGAACGC-

AGCGAAATGCGATAAGTAATGTGAATTGCAGAATTCAGTGAATCATCGAATCTTTGAACG

CACATTGCGCCCGCCGGCACTCCGGCGGGCATGCCTGTCCGAGCGTCATTTCAACCCTCA

GGG-CCCC--CTTTC------GGGGG-----------GGACCTGGTGCTGGGGATCAG-C

------------------------GGCCCTC--------GGGCCC-CTGTCCCC-CAAAT

ACAGTGGCGGTCGCGCCGCAGCC-TCCCCTGCGTAGTAGC-----ACA--CCTC--GCAC

C-GGAG--AGCGGTTCGACCAC-GCCGTAAAACCCCC-A-ACTTT---CCAA-GG

>Trichothecium_sp_F136858, 627 bases, 0 checksum..

ACC-GAGTTT----AC---ACAAACTCCC-AACCC-TTTGTGAACC-TTACC--TACC--

GTTGCTTCGGCGG-A------CCGCCCC-GGGCGCT---GC-----------GT-GCCCC

GGAC-CCA--------GGCGCCCGCCGGGG-ACCATT--CAAACCCT--GTTTTTT----

---AACCA---GTGTAT--CTTCTGAG----CGAGCCGAAAGGC--------AACAAAA-

---CAA--TCAAAACTTTCAACAACGGATCTCTTGGTTCTGGCATCGATG-AAGAACGC-

AGCGAAATGCGATAAGTAATGTGAATTGCAGAATTCAGTGAATCATCGAATCTTTGAACG

CACATTGCGCCCGCCAGTATTCTGGCGGGCATGCCTGTCCGAGCGTCATTTCAACCCTCG

GGCCCCCCC----TCTAAC-CGGGGG---------CGGGAC-CGGTGTTGGGGCTCAGGC

-------------------------GTCCTCCTCTT---GGGCGC-CTGTCCCC-TAAAT

GCAGTGGCGGCCTCGCCGCTGCC-TCCTCCGCGTAGTAGC-----ACAAACCTC--GCGT

GTGGAA--GGCGGCGCGGCCAC-GCCGTAAAACCCCC-A-ACTTT-TACCAA-GG

>Trichothecium_hongkongense_CBS117586 Trichothecium hongkongense culture CBS:117586 small subunit ribosomal RNA gene, partial sequence; internal transcribed spacer 1 and 5.8S ribosomal RNA gene, complete sequence; and internal transcribed spacer 2, partial sequence.

ACC-GAGTTT----AC---ACAAACTCCC-AACCC-TTTGTGAACC-TTACC--TACC--

GTTGCTTCGGCGG-A------CCGCCCC-GGGCGCT---GC-----------GT-GCCCC

GGAC-CCA--------GGCGCCCGCCGGGG-ACCATT--CAAACCCT--GTTTTTT----

---AACCA---GTGTAT--CTTCTGAG----CGAGCCGAAAGGC--------AACAAAA-

---CAA--TCAAAACTTTCAACAACGGATCTCTTGGTTCTGGCATCGATG-AAGAACGC-

AGCGAAATGCGATAAGTAATGTGAATTGCAGAATTCAGTGAATCATCGAATCTTTGAACG

CACATTGCGCCCGCCAGTATTCTGGCGGGCATGCCTGTCCGAGCGTCATTTCAACCCTCG

GGCCCCCCC----TCTAAC-CGGGGG---------CGGGAC-CGGTGTTGGGGCTCAGGC

-------------------------GTCCTCCTCTT---GGGCGC-CTGTCCCC-TAAAT

GCAGTGGCGGCCTCGCCGCTGCC-TCCTCCGCGTAGTAGC-----ACAAACCTC--GCGT

GTGGAA--GGCGGCGCGGCCAC-GCCGTAAAACCCCC-A-ACTTT-TACCAA---

>Trichothecium_hongkongense_CBS101444T Trichothecium hongkongense culture CBS:101444 small subunit ribosomal RNA gene, partial sequence; internal transcribed spacer 1 and 5.8S ribosomal RNA gene, complete sequence; and internal transcribed spacer 2, partial sequence.

ACC-GAGTTT----AC---ACAAACTCCC-AACCC-TTTGTGAACC-TTACC--TACC--

GTTGCTTCGGCGG-A------CCGCCCC-GGGTGCT---GC-----------GT-GCCCC

GGAC-CCA--------GGCGCCCGCCGGGG-ACCATT--CAAACCCT--GTTTTTT----

---AATCA---GTGTAT--CTTCTGAG----CGAGCCGAAAGGC--------AACAAAA-

---CAA--TCAAAACTTTCAACAACGGATCTCTTGGTTCTGGCATCGATG-AAGAACGC-

AGCGAAATGCGATAAGTAATGTGAATTGCAGAATTCAGTGAATCATCGAATCTTTGAACG

CACATTGCGCCCGCCAGTATTCTGGCGGGCATGCCTGTCCGAGCGTCATTTCAACCCTCG

GGCCCCCCC----TCTAAC-CGGGGG---------CGGGAC-CGGTGTTGGGGCTCAGGC

-------------------------GTCCTTCTC-----GGGCGC-CTGTCCCC-TAAAT

GCAGTGGCGGCCTCGCCGCTGCC-TCCTCCGCGTAGTAGC-----ACAAACCTC--GCGG

CTGGAA--GGCGGCGCGGCCAC-GCCGTGAAACCCCC-A-ACTTT-TACCAA---

>Trichothecium_hongkongense_CBS102186 Trichothecium hongkongense culture CBS:102186 small subunit ribosomal RNA gene, partial sequence; internal transcribed spacer 1 and 5.8S ribosomal RNA gene, complete sequence; and internal transcribed spacer 2, partial sequence.

ACC-GAGTTT----AC---ACAAACTCCC-AACCC-TTTGTGAACC-TTACC--TACC--

GTTGCTTCGGCGG-A------CCGCCCC-GGGTGCT---GC-----------GT-GCCCC

GGAC-CCA--------GGCGCCCGCCGGGG-ACCATT--CAAACCCT--GTTTTTT----

---AATCA---GTGTAT--CTTCTGAG----CGAGCCGAAAGGC--------AACAAAA-

---CAA--TCAAAACTTTCAACAACGGATCTCTTGGTTCTGGCATCGATG-AAGAACGC-

AGCGAAATGCGATAAGTAATGTGAATTGCAGAATTCAGTGAATCATCGAATCTTTGAACG

CACATTGCGCCCGCCAGTATTCTGGCGGGCATGCCTGTCCGAGCGTCATTTCAACCCTCG

GGCCCCCCC----TCTAAC-CGGGGG---------CGGGAC-CGGTGTTGGGGCTCAGGC

-------------------------GTCCTTCTC-----GGGCGC-CTGTCCCC-TAAAT

GCAGTGGCGGCCTCGCCGCTGCC-TCCTCCGCGTAGTAGC-----ACAAACCTC--GCGG

CTGGAA--GGCGGCGCGGCCAC-GCCGTGAAACCCCC-A-ACTTT-TACCAA---

>Hypocreales_sp_EU164804, 627 bases, 0 checksum..

ACC-GAGTTT----AC---ACAAACTCCC-AACCC-TTTGTGAACC-TTACC--TACC--

GTTGCTTCGGCGG-A------CCGCCCC-GGGTGCT---GC-----------GT-GCCCC

GGAC-CCA--------GGCGCCCGCCGGGG-ACCATT--CAAACCCT--GTTTTTT----

---AATCA---GTGTAT--CTTCTGAG----CGAGCCGAAAGGC--------AACAAAA-

---CAA--TCAAAACTTTCAACAACGGATCTCTTGGTTCTGGCATCGATG-AAGAACGC-

AGCGAAATGCGATAAGTAATGTGAATTGCAGAATTCAGTGAATCATCGAATCTTTGAACG

CACATTGCGCCCGCCAGTATTCTGGCGGGCATGCCTGTCCGAGCGTCATTTCAACCCTCG

GGCCCCCCC----TCTAAC-CGGGGG---------CGGGAC-CGGTGTTGGGGCTCAGGC

-------------------------GTCCTTCTC-----GGGCGC-CTGTCCCC-TAAAT

GCAGTGGCGGCCTCGCCGCTGCC-TCCTCCGCGTAGTAGC-----ACAAACCTC--GCGG

CTGGAA--GGCGGCGCGGCCAC-GCCGTGAAACCCCC-A-ACTTT-TACCAA-GG

>Endophyte_IB211_JX156018 Fungal endophyte strain IB211 18S ribosomal RNA gene, internal transcribed spacer 1, 5.8S ribosomal RNA gene, internal transcribed spacer 2, and 28S ribosomal RNA gene, region.

ACC-GAGTTT----AC-------ACTCCC-AACCC-TTTGTGAACC-TTACC--TACC--

GTTGCTTCGGCGG-A------CCGCCCC-GGGCGCT---GC-----------GT-GCCCC

GGAC-CCA--------GGCGCCCGCCGGGG-ACCATT--CAAACCCT--GTTTTTT----

----AACA--TGTGTAT--CTTCTGAG----CGAGCCGAAAGGC--------AACAAAA-

---CAA--TCAAAACTTTCAACAACGGATCTCTTGGTTCTGGCATCGATG-AAGAACGC-

AGCGAAATGCGATAAGTAATGTGAATTGCAGAATTCAGTGAATCATCGAATCTTTGAACG

CACATTGCGCCCGCCAGTATTCTGGCGGGCATGCCTGTCCGAGCGTCATTTCAACCCTCG

GGCCCCCG---TTT----G-CGCGGG---------CGG--CCCGGCGTTGGGGATCAGGA

-------------------------GCCCTTCTC------GGCGC-CTGCCCCC-TAAAT

CCAGTGGCGGCCTCGCCGCTGCC-TCCTCCGCGTAGTAGC-----ACAAACCTC--GCGG

CTGGAA--GGCGGCGCGGCCAC-GCCGTAAAACCCCC-A-ACT-T-TACCAA-GG

>Trichothecium_indicum_CBS12378_OQ429889 Trichothecium indicum culture CBS:123.78 small subunit ribosomal RNA gene, partial sequence; internal transcribed spacer 1 and 5.8S ribosomal RNA gene, complete sequence; and internal transcribed spacer 2, partial sequence.

ACC-GAGTTT----AC-------ACTCCC-AACCC-TTTGTGAACC-ATACC--TACC--

GTTGCTTCGGCGG-A------CCGCCCC-GGGCGCT---GC-----------GT-GCCCC

GGAC-CCA--------GGCGCCCGCCGGGG-ACCACT--CAAACCCT--GTTTTTTTTTT

TTTGAACA--TGTGTAT--CTTCTGAG----CGAGCCGAAAGGCCTCGTAAAAACAAAA-

---CAA--TCAAAACTTTCAACAACGGATCTCTTGGTTCTGGCATCGATG-AAGAACGC-

AGCGAAATGCGATAAGTAATGTGAATTGCAGAATTCAGTGAATCATCGAATCTTTGAACG

CACATTGCGCCCGCCAGTATTCTGGCGGGCATGCCTGTCCGAGCGTCATTTCAACCCTCG

GGCTCCCC---TCTCTT---CGAGGG---------CGGG-CCCGGCGTTGGGGCTCAGGC

------------------------GCCGTCTCTGC---TCGGCGC-CTGTCCCC-TAAAT

GCAGTGGCGGCCTCGCCGCTGCC-TCCTCCGCGTAGTAGC-----ACAAACCTC--GCGG

CTGGAA--GGCGGCGCGGCCAC-GCCGTAAAACCCCCCA-ACTTT-TA-------

>Trichothecium_sp_CBS25477 Trichothecium sp. culture CBS:254.77 small subunit ribosomal RNA gene, partial sequence; internal transcribed spacer 1 and 5.8S ribosomal RNA gene, complete sequence; and internal transcribed spacer 2, partial sequence.

ACC-GAGTTT----AC-------ACTCCC-AACCC-TTTGTGAACC-TTACC--TACC--

GTTGCTTCGGCGG-A------CCGCCCC-GGGTGCT---GC-----------GT-GCCCC

GGAC-CCA--------GGCGCCCGCCGGGG-ACCATC--CAAACCCT--GTTTTTT----

---AAACA--TGTGTAT--CTTCTGAG----CGAGCCGAAAGGC--------AACAAAA-

---CAA--TCAAAACTTTCAACAACGGATCTCTTGGTTCTGGCATCGATG-AAGAACGC-

AGCGAAATGCGATAAGTAATGTGAATTGCAGAATTCAGTGAATCATCGAATCTTTGAACG

CACATTGCGCCCGCCAGTATTCTGGCGGGCATGCCTGTCCGAGCGTCATTTCAACCCTCG

GGCCCCCG---TTT----G-CGCGGG---------CGG--CCCGGCGTTGGGGATCAGGA

-------------------------GCCTCC-------ACGGCGCCCTGTCCCC-TAAAT

GCAGTGGCGGCCTCGCCGCTGCC-TCCTCCGCGTAGTAGC-----ACAAACCTC--GCGG

CTGGAA--GGCGGCGCGGCCAC-GCCGTAAAACCCCC-A-ACTTT-TACCAA---

>Trichothecium_sympodiale_AB019365_JCM10014, 627 bases, 0 checksum..

--C-GAGTTT----AC------AACTCCC-AACCC-TTTGTGAACC-TTACC--TATC--

GTTGCTTCGGCGG-A------CCGCCCC-GGGTGCT---GC-----------GT-GCCCC

GGAC-TCA--------GGCGCCCGCCGGGG-ACCAAC--CAAACCCT--GTTTTTT----

---AAACA--TGTGTAT--CATCTGAG----CGAGCCGAAAGG---------AACAAA--

---CAA--TCAAAACTTTCAACAACGGATCTCTTGGTTCTGGCATCGATG-AAGAACGC-

AGCGAAATGCGATAAGTAATGTGAATTGCAGAATTCAGTGAATCATCGAATCTTTGAACG

CACATTGCGCCCGCCAGTATTCTGGCGGGCATGCCTGTTCGAGCGTCATTTCAACCCTCG

GGCCCCCGC---TTCT----CGCGGG---------CGG---CC-GCGTTGGGGCTCAGGC

---------------------G-CCGT-CT-CTGCT---CGGCGC-CTGTTCCCCTAAAT

GCAGTGGCGGCCTCGC-GTTGCC-TCCTCCGCGTAGTAGC-----ACAAACCTC--GCGG

CTGGAA--GGCGGCGCGGCCAC-GC-GTAAAACCCCCCA-ACTAT-TACCAA-GG

>Trichothecium_crotocinigenum_DQ882846, 627 bases, 0 checksum..

-CC-GAGTTT----AC-------ACTCCC-AACCC-TTTGTGAACC-TTACC--TACC--

GTTGCTTCGGCGG-A------CCGCCCC-GGGCGCT---GC-----------GT-GCCCC

GGAC-CCA--------GGCGCCCGCCGGGG-ACCACT--CAAACCCT--GTTTTTGTTT-

TCAAAACA--TGTGTAT--CTTCTGAG----CGAGCCGAAAGGCG---------CAAAAA

A--CAA--TCAAAACTTTCAACAACGGATCTCTTGGTTCTGGCATCGATG-AAGAACGC-

AGCGAAATGCGATAAGTAATGTGAATTGCAGAATTCAGTGAATCATCGAATCTTTGAACG

CACATTGCGCCCGCCAGTATTCTGGCGGGCATGCCTGTCCGAGCGTCATTTCAACCCTCG

GGCCTCCCC----TCT-----GCGGG---------CGG--CCCGGCGTTGGGGCTCAGG-

--------------------TG-CCGT-CT-CTGCT---CGGCGC-CTGTCCCC-TAAAT

GCAGTGGCGGCCTCGCCGCTGCC-TCCTCCGCGTAGTAGC-----ACAAACCTC--GCGG

CTGGAA--GGCGGCGCGGCCAC-GCCGTAAAACCCCC-A-ACT-T-TACCAA-G-

>Trichothecium_crotocinigenum_EU543259_Dzf16, 627 bases, 0 checksum..

ACC-GAGTTT----AC-------ACTCCC-AACCC-TTTGTGAACC-TTACC--TACC--

GTTGCTTCGGCGG-A------CCGCCCC-GGGCGCT---GC-----------GT-GCCCC

GGAC-CCA--------GGCGCCCGCCGGGG-ACCACT--CAAACCCT--GTTTTTGTTT-

TCAAAACA--TGTGTAT--CTTCTGAG----CGAGCCGAAAGGCG---------CAAAAA

AAACAA--TCAAAACTTTCAACAACGGATCTCTTGGTTCTGGCATCGATG-AAGAACGC-

AGCGAAATGCGATAAGTAATGTGAATTGCAGAATTCAGTGAATCATCGAATCTTTGAACG

CACATTGCGCCCGCCAGTATTCTGGCGGGCATGCCTGTCCGAGCGTCATTTCAACCCTCG

GGCCTCCCC----TCT-----GCGGG---------CGG--CCCGGCGTTGGGGCTCAGG-

--------------------TG-CCGT-CT-CTGCT---CGGCGC-CTGTCCCC-TAAAT

GCAGTGGCGGCCTCGCCGCTGCC-TCCTCCGCGTAGTAGC-----ACAAACCTC--GCGG

CTGGAA--GGCGGCGCGGCCAC-GCCGTAAAACCCCC-A-ACT-T-TACCAA-GG

>Trichothecium_sp_GU183172, 627 bases, 0 checksum..

ACC-GAGTTT----AC------AACTCCC-AACCC-TTTGTGAACC-TTACC--TATC--

GTTGCTTCGGCGG-A------CCGCCCC-GGGTGCT---GC-----------GT-GCCCC

GGAC-TCA--------GGCGCCCGCCGGGG-ACCAAC--CAAACCCT--GTTTTTT----

---AAACA--TGTGTAT--CATCTGAG----CGAGCCGAAAGGC--------AACAAA--

---CAA--TCAAAACTTTCAACAACGGATCTCTTGGTTCTGGCATCGATG-AAGAACGC-

AGCGAAATGCGATAAGTAATGTGAATTGCAGAATTCAGTGAATCATCGAATCTTTGAACG

CACATTGCGCCCGCCAGTATTCTGGCGGGCATGCCTGTTCGAGCGTCATTTCAACCCTCG

GGCCCCCGC---TTCT----CGCGGG---------CGG--CCCGGCGTTGGGGCTCAGGC

---------------------G-CCGT-CT-CTGCT---CGGCGC-CTGTCCCC-TAAAT

GCAGTGGCGGCCTCGCCGCTGCC-TCCTCCGCGTAGTAGC-----ACAAACCTC--GCGG

CTGGAA--GGCGGCGCGGCCAC-GCCGTAAAACCCCC-A-ACTAT-TACCAA-GG

>Trichothecium_ovalisporum_EU445372, 627 bases, 0 checksum..

ACC-GAGTTT----AC------AACTCCC-AACCC-TTTGTGAACC-TTACC--TATC--

GTTGCTTCGGCGG-A------CCGCCCC-GGGTGCT---GC-----------GT-GCCCC

GGAC-TCA--------GGCGCCCGCCGGGG-ACCAAC--CAAACCCT--GTTTTTT----

---AAACA--TGTGTAT--CATCTGAG----CGAGCCGAAAGGC--------AACAAA--

---CAA--TCAAAACTTTCAACAACGGATCTCTTGGTTCTGGCATCGATG-AAGAACGC-

AGCGAAATGCGATAAGTAATGTGAATTGCAGAATTCAGTGAATCATCGAATCTTTGAACG

CACATTGCGCCCGCCAGTATTCTGGCGGGCATGCCTGTTCGAGCGTCATTTCAACCCTCG

GGCCCCCGC---TTCT----CGCGGG---------CGG--CCCGGCGTTGGGGCTCAGGC

---------------------G-CCGT-CT-CTGCT---CGGCGC-CTGTCCCC-TAAAT

GCAGTGGCGGCCTCGCCGCTGCC-TCCTCCGCGTAGTAGC-----ACAAACCTC--GCGG

CTGGAA--GGCGGCGCGGCCAC-GCCGTAAAACCCCC-A-ACTAT-TACCAA-GG

>Trichothecium_roseum_JQ434580, 627 bases, 0 checksum..

ATA-GAGTTA----ACAAAAC-AACTCCC-AACCC-TTTGTGAACC-TTACC--TACC--

GTTGCTTCGGCGG-A------CCGCCCC-GGGCGCT---GC-----------GT-GCCCC

GGAC-CCAA-------GGCGCCCGCCGGGG-ACCACA--CGAACCCT--GTTTAA-----

--CAAACA--TGTGTAT--CCTCTGAG----CGAGCCGAAAGGC--------AACAAAA-

---CAAA-TCAAAACTTTCAACAACGGATCTCTTGGTTCTGGCATCGATG-AAGAACGC-

AGCGAAATGCGATAAGTAATGTGAATTGCAGAATTCAGTGAATCATCGAATCTTTGAACG

CACATTGCGCCCGCCAGTATTCTGGCGGGCATGCCTGTCCGAGCGTCATTTCAACCCTCG

GGCCCCCCCCTTTTCCCCT-CGCGGGGGA-GGGGGCGGG-CCCGGCGTTGGGGCCCAGGC

-------------------------GTCCTCC--AAG---GGCGC-CTGTCCCC-GAAAC

CCAGTGGCGGCCTCGCCGCTGCC-TCCTCCGCGTAGTAGC-----ACAAACCTC--GCGG

GCGGAA--GGCGGCGCGGCCAC-GCCGTAAAACCCCA-A-ACTTT-TACCAA-GG

>Trichothecium_roseum_EU552162_CBS113334, 627 bases, 0 checksum..

ATA-GAGTTA----ACAAAAC-AACTCCC-AACCC-TTTGTGAACC-TTACC--TACC--

GTTGCTTCGGCGG-A------CCGCCCC-GGGCGCT---GC-----------GT-GCCCC

GGAC-CCAA-------GGCGCCCGCCGGGG-ACCACA--CGAACCCT--GTTTAA-----

--CAAACA--TGTGTAT--CCTCTGAG----CGAGCCGAAAGGC--------AACAAAA-

---CAAA-TCAAAACTTTCAACAACGGATCTCTTGGTTCTGGCATCGATG-AAGAACGC-

AGCGAAATGCGATAAGTAATGTGAATTGCAGAATTCAGTGAATCATCGAATCTTTGAACG

CACATTGCGCCCGCCAGTATTCTGGCGGGCATGCCTGTCCGAGCGTCATTTCAACCCTCG

GGCCCCCCCCTTTTCCCCT-CGCGGGGGA-GGGGGCGGG-CCCGGCGTTGGGGCCCAGGC

-------------------------GTCCTCC--AAG---GGCGC-CTGTCCCC-GAAAC

CCAGTGGCGGCCTCGCCGCTGCC-TCCTCCGCGTAGTAGC-----ACAAACCTC--GCGG

GCGGAA--GGCGGCGCGGCCAC-GCCGTAAAACCCCA-A-ACTTT-TACCAA-GG

>Myrotheciomyces_corymbiae_CPC33206_NR_160351 Myrotheciomyces corymbiae CPC 33206 ITS region; from TYPE material.

ACT-GAGTTTT---AC----CAAACTCCC-AACCCCTATGTGAACC-ATACCTTTCCACT

GTTGCTTCGGCGGGA-----CACGCCCC-GGGTGCT---GC-----------GC-GCCCC

GGAA-CCA--------GGCGCCCGCCGGGG-ACCATTTACAAACTCTT-GTTTCTT----

----ACTA---CTGTAT--CTTCTGAGCAAAACCGCCGAAAGGCG------AAACAAAA-

---CAA--TCAAAACTTTCAACAACGGATCTCTTGGTTCTGGCATCGATG-AAGAACGC-

AGCGAAATGCGATAAGTAATGTGAATTGCAGAATTCAGTGAATCATCGAATCTTTGAACG

CACATTGCGCCCGCCAGTATTCTGGCGGGCATGCCTGTCCGAGCGTCATTTCAACCCTCG

CCCACTCCCC-TTT----A-GGGGGGAGA------CGGG---CGGTGTTGGGGCTCAGGC

CGAAGGCGGCGGTTCTCCGGAACCCGCCC----GCCCCACGG-GC-CTGTCCCC-TAAAT

GCAGTGGCGGCCTCGCCGCTGCC-TCCTCCGCGTAGTAGC-----ACAAACCTC--GCGG

GTGGAA--GGCGGCGCGGCCACAGCCGTAAAACCCCCCA-ACTTTTT-C-AA-GG

>Stilbella_albocitrina_CF255045_CBS52684 ..

ACC-GAGTTT----AC---ACAAACTCCC-AACCC-ACTGTGAACC-TTACC--TATC--

GTTGCTTCGGCGG-A------CCGCCCC-GGGTGCT---GC-----------GT-GCCCC

GGAA-CCA--------GGCGCCCGCCGGGG-ACCA-T--CAAACCCT--GTTTTTT----

-------A--TGTGAAT--CTTCTGAG-----TGGCCGAAAGGC-------AAACAAAA-

---CAA--TCAAAACTTTCAACAACGGATCTCTTGGTTCTGGCATCGATG-AAGAACGC-

AGCGAAATGCGATAAGTAATGTGAATTGCAGAATTCAGTGAATCATCGAATCTTTGAACG

CACATTGCGCCCGCCAGTATTCTGGCGGGCATGCCTGTCCGAGCGTCATTTCAACCCTCG

GGACCCCG---TTT--GCT-CG--------------GGGCCCCGGTGTTGGGGCTCAGGC

-------------------------GTCCT----ACCCAGGGCGC-CTGTCCCC-TAAAT

GCAGTGGCGGCCTCGCCGCTGCC-TCCTCCGCGTAGTAGC-----ACAAACCTC--GCGG

GTGGAA--GGCGGCGCGGCCAC-GCCGTAAAACCCCC-A-AATTT-TACCAA-GG

>Stanjemonium_spectabile_CF255041_CBS3407 ..

ACT-GAGTTT----AC---ACAAACTCCC-AACCC-TATGTGAACC-TTACCTTTACC--

GTTGCTTCGGCGGG-------CCGCCCC-GGGTGCT---GC-----------GT-GCCCC

GGAC-CCA--------GGCGCCCGCCGGGG-AC-ACC--CAAACTCT--GTATTT-----

----ACCA--AGTGTAT--CTTTTGAG-----CCGCCGAAAGGCG-----AAAACAAAAC

---GAA--TCAAAACTTTCAACAACGGATCTCTTGGTTCTGGCATCGATG-AAGAACGC-

AGCGAAATGCGATAAGTAATGTGAATTGCAGAATTCAGTGAATCATCGAATCTTTGAACG

CACATTGCGCCCGCCAGTATTCTGGCGGGCATGCCTGTCCGAGCGTCATTTCAACCCTCG

GGCCCCCC-CTTTTCCCCTTCACGGGGGACGGGGGCGGG-CCCGGTGTTGGGGCTCAGGC

---------------------G-CCG--CT----AAGCCGGCCGC-CTGTCCCC-TAAAT

CCAGTGGCGGCCTCGCCGCTGCC-TCCTCCGCGTAGTAGC-----ACAAACCTC--GCGG

TTGGAA--GGCGGCGCGGCCAC-GCCGTAAAACCCCC-G-ACTTTTTACCAA-GG

>Aphanocladium_spectabile_NBRC101148 spectabile, NBRC101148, ITS-LSU rDNA D1D2, CC00779401, .

ACT-GAGTTT----AC---ACAAACTCCC-AACCC-TATGTGAACC-TTACCTTTACC--

GTTGCTTCGGCGGG-------CCGCCCC-GGGTGCT---GC-----------GT-GCCCC

GGAC-CCA--------GGCGCCCGCCGGGG-AC-ACC--CAAACTCT--GTATTT-----

----ACCA--AGTGTAT--CTTCTGAG-----CCGCCGAAAGGCG-----AAAACAAAAC

---GAA--TCAAAACTTTCAACAACGGATCTCTTGGTTCTGGCATCGATG-AAGAACGC-

AGCGAAATGCGATAAGTAATGTGAATTGCAGAATTCAGTGAATCATCGAATCTTTGAACG

CACATTGCGCCCGCCAGTATTCTGGCGGGCATGCCTGTCCGAGCGTCATTTCAACCCTCG

GGCCCCCC-CTTTTCCCCTTCACGGGGGACGGGGGCGGG-CCCGGTGTTGGGGCTCAGGC

---------------------G-CCG--CT----AAGCCGGCCGC-CTGTCCCC-TAAAT

CCAGTGGCGGCCTCGCCGCTGCC-TCCTCCGCGTAGTAGC-----ACAAACCTC--GCGG

TTGGAA--GGCGGCGCGGCCAC-GCCGTAAAACCCCC-G-ACTTTTTACCAA-GG

>Stanjemonium_spectabile_CF278320 Aphanocladium VG its_28s..

ACT-GAGTTT----AC---ACAAACTCCC-AACCC-TATGTGAACC-TTACCTTTACC--

GTTGCTTCGGCGGG-------CCGCCCC-GGGTGCT---GC-----------GT-GCCCC

GGAC-CCA--------GGCGCCCGCCGGGG-AC-ACC--CAAACTCT--GTATTT-----

----ACCA--AGTGTAT--CTTCTGAG-----CCGCCGAAAGGCG-----AAAACAAAAC

---GAA--TCAAAACTTTCAACAACGGATCTCTTGGTTCTGGCATCGATG-AAGAACGC-

AGCGAAATGCGATAAGTAATGTGAATTGCAGAATTCAGTGAATCATCGAATCTTTGAACG

CACATTGCGCCCGCCAGTATTCTGGCGGGCATGCCTGTCCGAGCGTCATTTCAACCCTCG

GGCCCCCC-CTTTTCCCCTTCACGGGGGACGGGGGCGGG-CCCGGTGTTGGGGCTCAGGC

---------------------G-CCG--CT----AAGCCGGCCGC-CTGTCCCC-TAAAT

CCAGTGGCGGCCTCGCCGCTGCC-TCCTCCGCGTAGTAGC-----ACAAACCTC--GCGG

TTGGAA--GGCGGCGCGGCCAC-GCCGTAAAACCCCC-G-ACTTTTTACCAA-GG

>Stanjemonium_grisellum_CBS100389_CF241431 ..

ACT-GAGTTA----TC----C-AACTCCCAAACCC--CTGTGAAC--ATACC--TAC---

GTTGCTTCGGCGGG-------CCGTCCCGCGGCGC----GC-CCACGTGG-CGT-GACCC

GGAC-CCA--------GGCGCCCGCCGGGG-ACC-CC--CAAACTCTT-GTTTT------

-----CCA---GTGTCT--CCTCTGAG----TGG--CATAA-GC---------A-AAAA-

---TAAA--CAAAACTTTCAGCAACGGATCTCTTGGTTCTGGCATCGATG-AAGAACGC-

AGCGAAATGCGATAAGTAATGTGAATTGCAGAATTCAGTGAATCATCGAATCTTTGAACG

CACATTGCGCCCGCCAGTATTCTGGCGGGCATGCCTGTCTGAGCGTCATTTCAACCCTCA

G-CCCCCG------CT----CGCGGGG--------CG----CTGGCGTTGGGGAT-CGGC

C------------------------GTCCTC--G-----CGGCGG-CCGGCCCC-GAAAC

ACAGTGGCGGTCTC-TCGCGGAC-TCCCCTGCGTAGTAGC-----ACT-ACCTC--GCAG

AAGGGACGAGCGGGCTGACCAC-GCCGTAAAACCCCCC--ACT-TCT-CCA--GG

>Stanjemonium_grisellum_CBS65579 Stanjemonium grisellum CBS 655.79 ITS region; from TYPE material.

ACT-GAGTTA----TC----C-AACTCCCAAACCC--CTGTGAAC--ATACC--TAC---

GTTGCTTCGGCGGG-------CCGTCCCGCGGCGC----GC-CCACGTGG-CGT-GACCC

GGAC-CCA--------GGCGCCCGCCGGGG-ACC-CC--CAAACTCTT-GTTTT------

-----CCA---GTGTCT--CCTCTGAG----TGG--CATAA-GC---------A-AAAA-

---TAAA--CAAAACTTTCAGCAACGGATCTCTTGGTTCTGGCATCGATG-AAGAACGC-

AGCGAAATGCGATAAGTAATGTGAATTGCAGAATTCAGTGAATCATCGAATCTTTGAACG

CACATTGCGCCCGCCAGTATTCTGGCGGGCATGCCTGTCTGAGCGTCATTTCAACCCTCA

G-CCCCCG------CT----CGCGGGG--------CG----CTGGCGTTGGGGAT-CGGC

C------------------------GTCCTC--G-----CGGCGG-CCGGCCCC-GAAAC

ACAGTGGCGGTCTC-TCGCGGAC-TCCCCTGCGTAGTAGC-----ACT-ACCTC--GCAG

AAGGGACGAGCGGGCTGACCAC-GCCGTAAAACCCCCC--ACT-TCT-CCA--GG

>Stanjemonium_ochroroseum_CBS65679T Stanjemonium ochroroseum CBS 656.79 ITS region; from TYPE material.

ACT-GAGTTA----TC----C-AAATCCCAAACCC--CTGTGAAC--ATACC--TAC---

GTTGCTTCGGCGGG-------CCGTCCCGCGGCGC----GC-CCACGTGG-CGT-GACCC

GGAC-CCA--------GGCGCCCGCCGGGG-ACC--C--CAAACTCTT-GTTTT------

-----CCA---GTGTCT--CCTCTGAG----TGG--CATAA-GC---------A-AAAA-

---TAAA--CAAAACTTTCAGCAACGGATCTCTTGGTTCTGGCATCGATG-AAGAACGC-

AGCGAAATGCGATAAGTAATGTGAATTGCAGAATTCAGTGAATCATCGAATCTTTGAACG

CACATTGCGCCCGCCAGTATTCTGGCGGGCATGCCTGTCTGAGCGTCATTTCAACCCTCA

G-CCCCCG------CT----CGCGGGG--------CG----CTGGCGTTGGGGAT-CGGC

C------------------------GTCCTC--G-----CGGCGG-CCGGCCCC-GAAAC

ACAGTGGCGGTCTC-TCGCGGAC-TCCCCTGCGTAGTAGC-----ACT-ACCTC--GCAG

AAGGGACGAGCGGGCTGACCAC-GCCGTAAAACCCCC---ACT-TCT-CCA--GG

>Stanjemonium_fuscescens_CBS26496T Stanjemonium fuscescens CBS 264.96 ITS region; from TYPE material.

ACT-GAGTTA----TC----C-AACTCCCAAACCC--CTGTGAAC--ATACC--TAC---

GTTGCTTCGGCGGG-------CCGTCCCGCGGCGC----GC-CCACGTGG-CGT-GACCC

GGAC-CCC--------GGCGCCCGCCGGGG-ACC--C--CAAACTCTT-GTTTT------

-----CCA---GTGTCT--CCTCTGAG----TGG--CATAA-GC---------A-AAAA-

---TAAA--CAAAACTTTCAGCAACGGATCTCTTGGTTCTGGCATCGATG-AAGAACGC-

AGCGAAATGCGATAAGTAATGTGAATTGCAGAATTCAGTGAATCATCGAATCTTTGAACG

CACATTGCGCCCGCCAGTATTCTGGCGGGCATGCCTGTCTGAGCGTCATTTCAACCCTCA

G-CCCCCG------CT----CGCGGGG--------CG----CTGGCGTTGGGGCT-CGGC

C------------------------GTCCTC--G-----CGGCGG-CCGGCCCC-GAAAC

ACAGTGGCGGTCTC-TCGCGGAC-TCCCCTGCGTAGTAAC-----ACT-ACCTC--GCAG

AAGGGACGAGCGGGCGGACCAC-GCCGTAAAACCCCCC--ACT-TCT-CCA--GG

>Stanjemonium_dichromosporum_CBS63873T Stanjemonium dichromosporum CBS 638.73 ITS region; from TYPE material.

ACT-GAGTTA----TC----C-AACTCCCAAACCC--CTGTGAAC--ATACC--TAC---

GTTGCTTCGGCGGG-------CCGTCCCGCGGCGC----GC-CCACGTGG-CGT-GACCC

GGAC-CCA--------GGCGCCCGCCGGGG-ACC--C--CAAACTCTT-GTTTT------

-----CCA---GTGTCT--CCTCTGAG----TGG--CATAA-GC---------A-AAAA-

---TAAA--CAAAACTTTCAGCAACGGATCTCTTGGTTCTGGCATCGATG-AAGAACGC-

AGCGAAATGCGATAAGTAATGTGAATTGCAGAATTCAGTGAATCATCGAATCTTTGAACG

CACATTGCGCCCGCCAGTATTCTGGCGGGCATGCCTGTCTGAGCGTCATTTCAACCCTCA

G-CCCCCG------CT----CGCGGGG--------CG----CTGGCGTTGGGGAT-CGGC

C------------------------GTCCTC--G-----CGGCGG-CCGGCCCC-GAAAC

ACAGTGGCGGTCTC-CCGCGGAC-TCCCCTGCGTAGTAGC-----ACT-ACCTC--GCAG

AAGGGACGAGCGGGCTGGCCAC-GCCGTAAAACCCCCC--ACT-TCT-CAA--GG

>Emericellopsis_terricola_AB425992_NBRC7898 Emericellopsis terricola genes for ITS1, 5.8S rRNA, ITS2, partial sequence, strain: NBRC 7898.

ACT-GAGTTAT---CC------AACTCCCAAACCC--CTGTGAAC--ATACC--TA-T--

GTTGCTTCGGCGGG-------TCGTCCCGCGGCGC----GC-CCTCGTGG-CGT-GACCC

GGAC-CCA--------GGCGCCCGCCGGGG-AA---C--CAAACTCTT-GTCTT------

-----CCA---GTGTCT--CCTCTGAG----TGG--CATAA-GC---------A-AAAA-

---TAAA--CAAAACTTTCAGCAACGGATCTCTTGGTTCTGGCATCGATG-AAGAACGC-

AGCGAAATGCGATAAGTAATGTGAATTGCAGAATTCAGTGAATCATCGAATCTTTGAACG

CACATTGCGCCCGCCAGTATTCTGGCGGGCATGCCTGTCTGAGCGTCATTTCAACCCTCA

G-CCCCCG----TTC------GCGGGG--------CG----CTGGCGTTGGGGCC-CGGC

C------------------------GTCCTC--G-----CGGCGG-CCGTCCCC-GAAAC

ACAGTGGCGGTCTC-CCGCAGAC-TCCCCTGCGTAGTAGC-----ACT-ACCTC--GCAG

AAGGGACGAGCGGGC-GGCCAC-GCCGTAAAACACCCC--ACT-TCT-CCA--G-

>Emericellopsis_glabra_AY632657_CBS11940 Emericellopsis glabra strain CBS 119.40 internal transcribed spacer 1, 5.8S ribosomal RNA gene, and internal transcribed spacer 2, complete sequence; and 28S ribosomal RNA gene, partial sequence.

ACT-GAGTTAT---CC------AACTCCCAAACCC--CTGTGAAC--ATACC--TA-T--

GTTGCTTCGGCGGG-------CCGTCCCGCGGCGC----GC-CCACGTGG-CGT-GACC-

GGAC-CCA--------GGCGCCCGCCGGG--AA---C--CAAACTCTT-GTCTT------

------C--GAGTGTCT--CCTCTGAG----TGG--CATAA-GC---------A-AAAA-

---TAAA--CAAAACTTTCAGCAACGGATCTCTTGGTTCTGGCATCGATG-AAGAACGC-

AGCGAAATGCGATAAGTAATGTGAATTGCAGAATTCAGTGAATCATCGAATCTTTGAACG

CACATTGCGCCCGCCAGTATTCTGGCGGGCATGCCTGTCTGAGCGTCATTTCAACCCTCA

G-CCCCCG----TTC------GCGGGG--------CG----CTGGCGTTGGGGCC-CGGC

C------------------------GTCCTC--G-----CGGCGG-CCGTCCCC-GAAAC

ACAGTGGCGGTCTC-CCGCAGAC-TCCCCTGCGTAGTAGC-----ACT-ACCTC--GCAG

AAGGGACGAGCGGGCTGGCCAC-GCCGTAAAACACCCC--ACT-TCT-CCA--GG

>Emericellopsis_minima_AY632669_CBS19055 Emericellopsis minima strain CBS 190.55 internal transcribed spacer 1, 5.8S ribosomal RNA gene, and internal transcribed spacer 2, complete sequence.

ACT-GAGTTT----AC------AACTCCCAAACCC--CTGTGAAC--ATACC--TA-T--

GTTGCTTCGGCGGG-------CCGTCCCGCGGCGC----GC-CCACGTGG-CGT-GACCC

GGAA-CCA--------GGCG-CCGCCGGGG-AC---C--CAAACTCTT-GCCTTTT----

---------TCGTGTCT--CCTCTGAG----TGG--CATAA-GC---------A-AAAA-

---TAAA--CAAAACTTTCAGCAACGGATCTCTTGGTTCTGGCATCGATG-AAGAACGC-

AGCGAAATGCGATAAGTAATGTGAATTGCAGAATTCAGTGAATCATCGAATCTTTGAACG

CACATTGCCCCCGCCAGTATTCTGGCGGGCATGCCTGTCTGAGCGTCATTTCAACCCTCA

G-CCCCCG----TTC------GCGGGG--------CG----CTGGCGTTGGGGATCCGGC

C------------------------GTCCTC--G-----CGGCGG-CCGGCCCC-GAAAC

GCAGTGGCGGTCTC-T-GCGGAC-TCCCCTGCGTAGTAGC-----ACT-ACCTC--GCAG

AAGGGACGAGCGGGCTGGCCAC-GCCGTAAAACCCCC-A-ACT-TCT-CC-----

>Acremonium_potronii_JN021531, 627 bases, 0 checksum..

ACT-GAGTTT----AC------AACTCCCAAACCC--CTGTGAAC--ATACC--TA-T--

GTTGCTTCGGCGGG-------CCGTCCCGCGGCGC----GC-CCACGTGG-CGT-GACCC

GGAA-CCA--------GGCGCCCGCCGGGG-AC---C--CAAACTCTT-GCCTTTT----

---------TAGTGTCT--CCTCTGAG----TGG--CATAA-GC---------A-AAAA-

---TAAA--CAAAACTTTCAGCAACGGATCTCTTGGTTCTGGCATCGATG-AAGAACGC-

AGCGAAATGCGATAAGTAATGTGAATTGCAGAATTCAGTGAATCATCGAATCTTTGAACG

CACATTGCGCCCGCCAGTATTCTGGCGGGCATGCCTGTCTGAGCGTCATTTCAACCCTCA

G-CCCCCG----TTC------GCGGGG--------CG----CTGGCGTTGGGGAT-CGGC

C------------------------GCCCTCC-G-----CGGCGG-CCGGCCCC-GAAAC

ACAGTGGCGGTCTCCT-GCAGAC-TCCCCTGCGTAGTAGC-----ACT-ACCTC--GCAG

AAGGGACGAGCGGGCTGGCCAC-GCCGTAAAACCCCC-A-ACT-TCT--CAA-GG

>Acremonium_fuci_JQ796750, 627 bases, 0 checksum..

ACT-GAGTTT----AC------AACTCCCAAACCC--CTGTGAAC--ATACC--TA-T--

GTTGCTTCGGCGGG-------CCGTCCCGCGGCGC----GC-CCACGTGG-CGT-GACCC

GGAA-CCA--------GGCGCCCGCCGGGG-AC---C--CAAACTCTT-GCCT--GT---

----A----TAGTGTCT--CCTCTGAG----TGG--CATAA-GC---------A-AAAA-

---TAAA--CAAAACTTTCAGCAACGGATCTCTTGGTTCTGGCATCGATG-AAGAACGC-

AGCGAAATGCGATAAGTAATGTGAATTGCAGAATTCAGTGAATCATCGAATCTTTGAACG

CACATTGCGCCCGCCAGTATTCTGGCGGGCATGCCTGTCTGAGCGTCATTTCAACCCTCA

G-CCCCCG--C--TC------GCGGGG--------CG----CTGGCGTTGGGGCT-CGGC

C------------------------GTCCACC-G-----CGGCGG-CCGGCCCC-GAAAC

ACAGTGGCGGTCTC-CCGCGGAC-TCCC-TGCGTAGTAGC-----ACT-ACCTC--GCAG

AAGGGACGAGCGGGCTGGCCAC-GCCGTAAAACCCCCCA----------------

>Acremonium_hansfordii_AB540578_CBS39073 Acremonium hansfordii genes for ITS1, 5.8S rRNA, ITS2 and 28S rRNA, partial sequence, strain: CBS 390.73..

ATA-GAGTGT----A-------AACTCCCAAACC--TTTGTGAAC--ATACC-----T--

GTTGCTTCGGCGG--------TCCTCAC-CGGC-C----GC------------------C

-GAA------------GGC---------------CTT---ATATTCTT-GAATTT-----

----AC----A-TGAA---TTTCTGAG----TAT--CA-AA--C---------A-AAAA-

---TAAA-TAAAAACTTTCAGCAACGGATCTCTTGGCTCTGGCATCGATG-AAGAACGC-

AGCGAAATGCGATAAGTAATGTGAATTGCAGAATTCAGTGAATCATCGAATCTTTGAACG

CACATTGCGCCCACCAGTACTCTGGTGGGCATGCCTGTCCGAGCGTCATTTCAACCCTCA

GGGCCC-G----TTC------GCGGG-------------ACCTGGTGTTGGGGAT-CGGC

C--------------------------CCA-----------CCGG-CCGGCCCC-GAAAT

ACAGTGGCGGCACATCCGCGACC-TCCTCTGCGTAGTAGC-----AAT-GCCTC--GCAG

CTGGAT--AGCGGTTGCGCCTC-GCCGTAAAACCCCCC--ACT-TTT--CAATGG

>Acremonium_fusidioides_FN706543_UTHSC081188 Acremonium fusidioides 18S rRNA gene (partial), ITS1, 5.8S rRNA gene, ITS2 and 28S rRNA gene (partial), culture collection UTHSC:08-1188..

ATA-GAGTGT----A-------AACTCCCTAACC--TTTGTGAAC--ATACC-----T--

GTTGCTTCGGCGG--------TCCTCAC-CGGC-C----GC------------------C

-GAA------------GGC---------------CT----ATATTCTT-GAATTT-----

----AC----A-TGAA---TTTCTGAG----TAT--CA-AA--C---------A-AAAA-

---TAAA-TAAAAACTTTCAGCAACGGATCTCTTGGCTCTGGCATCGATG-AAGAACGC-

AGCGAAATGCGATAAGTAATGTGAATTGCAGAATTCAGTGAATCATCGAATCTTTGAACG

CACATTGCGCCCACCAGTACTCTGGTGGGCATGCCTGTCCGAGCGTCATTTCAACCCTCA

GGGCCC-G----TTC------GCGGG-------------ACCTGGTGTTGGGGAT-CGGC

C--------------------------CCA-----------CCGG-CCGGCCCC-GAAAT

ACAGTGGCGGCACATCCGCGACC-TCCTCTGCGTAGTAGC-----AAT-GCCTC--GCAG

CTGGAT--AGCGGTTGCGCCTC-GCCGTAAAACCCCCC--ACT-TTT--CAATGG

>Acremonium_fusidioides_FN706542_CBS84068 Acremonium fusidioides ITS1, 5.8S rRNA gene, ITS2 and 28S rRNA gene (partial), culture collection CBS:840.68..

--------GT----A-------AACTCCCTTACC--TTTGTGAAC--ATACC-----T--

GTTGCTTCGGCGG--------TCCTCAC-CGGC-C----GC------------------C

-GAA------------GGC---------------CT----ATATTCTT-GAATTT-----

----AC----A-TGAA---TTTCTGAG----TAT--CA-AA--C---------A-AAAA-

---TAAA-TAAAAACTTTCAGCAACGGATCTCTTGGCTCTGGCATCGATG-AAGAACGC-

AGCGAAATGCGATAAGTAATGTGAATTGCAGAATTCAGTGAATCATCGAATCTTTGAACG

CACATTGCGCCCACCAGTACTCTGGTGGGCATGCCTGTCCGAGCGTCATTTCAACCCTCA

GGGCCC-G----TTC------GCGGG-------------ACCTGGTGTTGGGGAT-CGGC

C--------------------------CCA-----------CCGG-CCGGCCCC-GAAAT

ACAGTGGCGGCACACCCGCGACC-TCCTCTGCGTAGTAGC-----AAT-GCCTC--GCAG

CTGGAT--AGCGGTTGCGCCTC-GCCGTAAAACCCCCC--ACT-TCT--CAAAGG

>Acremonium_sclerotigenum_FN706549_CBS28180 Acremonium sclerotigenum 18S rRNA gene (partial), ITS1, 5.8S rRNA gene, ITS2 and 28S rRNA gene (partial), culture collection CBS:281.80..

ACC-GAGTGT----AA-----AAACTCCCAAACC--ATTGTGAAC--CTACC---ACT--

GTTGCTTCGGCGGC------CTCGCCCC-GGGCGCGTTCGC-----------GC-GGCCC

GGAC-CCA--------GGCGTCCGCCGGAGG---CTC--CAAACTCTT-GTCTTT-----

---------TAGTGTA---TTTCTGAG----TGG--CATAA-GC-----------AAA--

---TAAA-TCAAAACTTTCAGCAACGGATCTCTTGGTTCTGGCATCGATG-AAGAACGC-

AGCAAAATGCGATAAGTAATGTGAATTGCAGAATTCAGTGAATCATCGAATCTTTGAACG

CACATTGCGCCCGCCAGTATTCTGGCGGGCATGCCTGTCTGAGCGTCATTTCAACCCTCA

GGACCC-G----TTC------GCGGG-------------ACCTGGCGTTGGGGATCA-GC

C-------------------TGCCC------CTGG----CGGCGG-CTGGCCCT-GAAAT

CCAGTGGCGGTTCCCTCGCGAAC-TCCTCCGTGCAGTAAT----TAA--ACCTCTCGCGG

CAGGAT--AGCGGTTGAACCAC-GCCGTTA-ACCCCCC--ACT-TCT--CAA-GG

>Acremonium_sclerotigenum_FN706552_CBS12442 Acremonium sclerotigenum ITS1, 5.8S rRNA gene and ITS2, culture collection CBS:124.42..

ACC-GAGTGT----AA-----AAACTCCCAAACC--ATTGTGAAC--CTACC---ACT--

GTTGCTTCGGCGGC------CTCGCCCC-GGGCGCGTTCGC-----------GC-GGCCC

GGAC-CCA--------GGCGTCCGCCGGAGG---CTC--CAAACTCTT-GTCTTT-----

---------TAGTGTA---TTTCTGAG----TGG--CATAA-GC-----------AAA--

---TAAA-TCAAAACTTTCAGCAACGGATCTCTTGGTTCTGGCATCGATG-AAGAACGC-

AGCAAAATGCGATAAGTAATGTGAATTGCAGAATTCAGTGAATCATCGAATCTTTGAACG

CACATTGCGCCCGCCAGTATTCTGGCGGGCATGCCTGTCTGAGCGTCATTTCAACCCTCA

GGACCC-G----TTC------GCGGG-------------ACCTGGCGTTGGGGATCA-GC

C-------------------TGCCC------CTGG----CGGCGG-CTGGCCCT-GAAAT

CCAGTGGCGGTTCCCTCGCGAAC-TCCTCCGTGCAGTAAT----TAA--ACCTCTCGCGG

CAGGAT--AGCGGTTGAACCAC-GCCGTTAAACCCCCC--ACT-TCT--CAA-G-

>Acremonium_sclerotigenum_FN706551_CBS27086 Acremonium sclerotigenum ITS1, 5.8S rRNA gene and ITS2, culture collection CBS:270.86..

--C-GAGTG-----AA-----AAACTCCCAAACC--ATTGTGAAC--TTACC---ACT--

GTTGCTTCGGCGGC------CTCGCCCC-GGGCGCGTTCGC-----------GC-GGCCC

GGAC-CCA--------GGCGTCCGCCGGAGG---CTC--CAAACTCTT-GTCTTT-----

---------TAGTGTA---TTTCTGAG----TGG--CATAA-GC-----------AAA--

---TAAA-TCAAAACTTTCAGCAACGGATCTCTTGGTTCTGGCATCGATG-AAGAACGC-

AGCAAAATGCGATAAGTAATGTGAATTGCAGAATTCAGTGAATCATCGAATCTTTGAACG

CACATTGCGCCCGCCAGTATTCTGGCGGGCATGCCTGTCTGAGCGTCATTTCAACCCTCA

GGACCC-G----TTC------GCGGG-------------ACCTGGCGTTGGGGATCA-GC

C-------------------TGCCC------CTGG----CGGCGG-CTGGCCCT-GAAAT

CCAGTGGCGGTTCCCTCGCGAAC-TCCTCCGTGCAGTAAT----TAA--ACCTCTCGCGG

CAGGAT--AGCGGTTGAACCAC-GCCGTTAAACCCCCC--ACT-TCT--CAA-GG

>Acremonium_brachypenium_AB540570_CBS86673 Acremonium brachypenium genes for ITS1, 5.8S rRNA, ITS2 and 28S rRNA, partial sequence, strain: CBS 866.73...

ACT-GAGTGT----AA-----AAACTCCCAAACCC-TATGCGAAC--CTACC---AAA--

GTTGCTTCGGCGG-A------CCGCCCC-GGGCGCCCT-GC-----------GC--ACCC

GGGC-CTA--------GGCGGCCGCCGGAGG---CTC--CAAACTCT--GAATTT-----

----AC----AGTGGA---TTTCTGAG----TGG--CATAA-GC-----------AAA--

---TAAA-TCAAAACTTTCAGCAACGGATCTCTTGGTTCTGGCATCGATG-AAGAACGC-

AGCAAAATGCGATAAGTAATGTGAATTGCAGAATTCAGTGAATCATCGAATCTTTGAACG

CACATTGCGCCCGCCAGTATTCTGGCGGGCATGCCTGTCTGAGCGTCATTTCAACCCTCG

GGACCCCG----TTC------GCGGG-------------ACCCGGCGTTGGGGATCA-GC

C--------------C-G-AAGCCC-T-CG-C-GGC---AGGCGG-CTGGCCCC-TAAAC

CTAGTGGCGGTCCTCCCGGCGACCTCCTCTGCGCAGTAGT---TTAAT--CGCCTCGCAG

CTGGAA--CGCGGGAAGGCCAC-GCCGTAAAACACCCA--ACTATTCA-CAA-GG

>Acremonium_persicinum_JQ012760, 627 bases, 0 checksum..

ACT-GAGTCT---AAC-----AAACTCCCAAACCC--CTGTGAAC--ATACC--TACT--

GTTGCTTCGGCGGGA------CCGCCCC-GGGCGCCTTCGCG----------GT-GCCCC

GGAA-CCA--------GGCGCCCGCCGGGG-AC-ATC---AAACTCTT-G-ATT-GTT--

--------ATAGTGGCAT-TCTCTGAG----TAAAACATA---C-----------AAA--

---TAAG-TCAAAACTTTCAACAACGGATCTCTTGGCTCTGGCATCGATG-AAGAACGC-

AGCGAAATGCGATAAGTAATGCGAATTGCAGAATTCAGTGAATCATCGAATCTTTGAACG

CACATTGCGCCCGCTAGTATTCTGGCGGGCATGCCTGTCTGAGCGTCATTTCAACCCTCG

C-CCCCGG--C-TTCT-----GCTGG------------GAG-CGGTGTTGGGGAT-CGGC

C----------------G----CCCGT-CA-CT------GGGAGG-CCGGCCCC-GAAAT

AGAGTGGCGACCACGCCGTGTGC-TCCTCTGCGTAGTAGT----AAATCACCTC--GCAG

GCGGAC--AGCGGTGCGGCC-T-GCCGTAAAACCCCC-A-ACT--CTTTCT--GG

>Acremonium_persicinum_FN706554_CBS31059 Acremonium persicinum 18S rRNA gene (partial), ITS1, 5.8S rRNA gene, ITS2 and 28S rRNA gene (partial), culture collection CBS:310.59..

CCT-GAGTCT---AAC-----AAACTCCCAAACCC--CTGTGAAC--ATACC--TACT--

GTTGCTTCGGCGGGA------CCGCCCC-GGGCGCCTTCGCG----------GT-GCCCC

GGAA-CCA--------GGCGCCCGCCGGGG-AC-ATC---AAACTCTT-G-ATT-GTT--

--------ATAGTGGCAT-TCTCTGAG----TAAAACATA---C-----------AAA--

---TAAG-TCAAAACTTTCAACAACGGATCTCTTGGCTCTGGCATCGATG-AAGAACGC-

AGCGAAATGCGATAAGTAATGCGAATTGCAGAATTCAGTGAATCATCGAATCTTTGAACG

CACATTGCGCCCGCTAGTATTCTGGCGGGCATGCCTGTCTGAGCGTCATTTCAACCCTCG

C-CCCCGG--C-TTTT-----GCTGG------------GAG-CGGTGTTGGGGAT-CGGC

C----------------G----CCCGT-CA-CT------GGGAGG-CCGGCCCC-GAAAT

AGAGTGGCGACCACGCCGTGTGC-TCCTCTGCGTAGTAGT----AAATCACCTC--GCAG

GCGGAC--AGCGGTGCGGCC-T-GCCGTAAAACCCCC-A-ACT--CTTTCTATGG

>Acremonium_persicinum_FN706546_UTHSC011389 Acremonium persicinum 18S rRNA gene (partial), ITS1, 5.8S rRNA gene, ITS2 and 28S rRNA gene (partial), culture collection UTHSC:01-1389..

ACT-GAGTCT---AAC-----AAACTCCCAAACCC--CTGTGAAC--ATACC--TACT--

GTTGCTTCGGCGGGA------CCGCCCC-GGGCGCCTTCGCG----------GT-GCCCC

GGAA-CCA--------GGCGCCCGCCGGGG-AC-ATC---AAACTCTT-G-ATT-GTT--

--------ATAGTGGCAT-TCTCTGAG----TAAAACATA---C-----------AAA--

---TAAG-TCAAAACTTTCAACAACGGATCTCTTGGCTCTGGCATCGATG-AAGAACGC-

AGCGAAATGCGATAAGTAATGCGAATTGCAGAATTCAGTGAATCATCGAATCTTTGAACG

CACATTGCGCCCGCTAGTATTCTGGCGGGCATGCCTGTCTGAGCGTCATTTCAACCCTCG

C-CCCCGG--C-TTTT-----GCTGG------------GAG-CGGTGTTGGGGAT-CGGC

C----------------G----CCCGT-CA-CT------GGGAGG-CCGGCCCC-GAAAT

AGAGTGGCGACCACGCCGTGTGC-TCCTCTGCGTAGTAGT----AAATCACCTC--GCAG

GCGGAC--AGCGGTGCGGCC-T-GCCGTAAAACCCCC-A-ACT--CTTTCT--GG

>Acremonium_persicinum_FN706545_UTHSC011249 Acremonium persicinum 18S rRNA gene (partial), ITS1, 5.8S rRNA gene, ITS2 and 28S rRNA gene (partial), culture collection UTHSC:01-1249..

ACT-GAGTCT---AAC-----AAACTCCCAAACCC--CTGTGAAC--ATACC--TACT--

GTTGCTTCGGCGGGA------CCGCCCC-GGGCGCCTTCGCG----------GT-GCCCC

GGAA-CCA--------GGCGCCCGCCGGGG-AC-ATC---AAACTCTT-G-ATT-GTT--

--------ATAGTGGCAT-TCTCTGAG----TAAAACATA---C-----------AAA--

---TAAG-TCAAAACTTTCAACAACGGATCTCTTGGCTCTGGCATCGATG-AAGAACGC-

AGCGAAATGCGATAAGTAATGCGAATTGCAGAATTCAGTGAATCATCGAATCTTTGAACG

CACATTGCGCCCGCTAGTATTCTGGCGGGCATGCCTGTCTGAGCGTCATTTCAACCCTCG

C-CCCCGG--C-TTTT-----GCTGG------------GAG-CGGTGTTGGGGAT-CGGC

C----------------G----CCCGT-CA-CT------GGGAGG-CCGGCCCC-GAAAT

AGAGTGGCGACCACGCCGTGTGC-TCCTCTGCGTAGTAGT----AAATCACCTC--GCAG

GCGGAC--AGCGGTGCGGCC-T-GCCGTAAAACCCCC-A-ACT--CTTTCT--GG

>Acremonium_polychromum_AB540566, 627 bases, 0 checksum..

ACC-GAGTT-----GC----AAAACTCCCAAACCC-ACTGTGAACCTCTACC---ACT--

GTTGCTTCGGCGG-A-----ACCGCCCC-GGGCGCACCTCCTC-ACGGGGGCGT-GCCCC

GGAA-CCA--------GGCGCCCGCCGGGGG---ACC--GAAACCTCT-GTATTTTTT--

----AC---TTGAGTA----CTCTGAG----TGT--GATTT------------ACAAAAT

---CAAAATTAAAACTTTCAACAACGGATCTCTTGGCTCTAGCATCGATG-AAGAACGC-

AGCGAAATGCGATAAGTAATGCGAATTGCAGAATTCAGTGAATCATCGAATCTTTGAACG

CACATTGCGCCCGCCAGTATTCTGGCGGGCATGCCTGTCTGAGCGTCGTTTCGACCCTCG

CG-CCCGG--C-TTCT----GTCGGGGG--------------CGGTGTTGGGGAT-CGGC

C---------------A---CACCC-T-C--C---AGA-GGGAGG-CCGGCCCC-TAAAT

CCAGTGGCGACCACGCTGTAGCC-TCCCCTGCGTAGTACT---AAAACCACCTC--GCAG

GCGGAG--AGCGGTGCGGCC-C-GCCGTAAAACCCCCCG-ACT-TTTA-CAA-GG

>Acremonium_polychromum_AB540567_CBS18127 Acremonium polychromum genes for ITS1, 5.8S rRNA, ITS2 and 28S rRNA, partial sequence, strain: CBS 181.27.

ACC-GAGTT-----GC----AAAACTCCCAAACCC-ACTGTGAACCTATACC---ACT--

GTTGCTTCGGCGG-A-----TACGCCCC-GGGCGCACCCCCTCCAGGGGGTTGT-GCCCC

GGAA-CCA--------GGCGCCCGCCGGGGG---ACC--GAAACCTCT-GTATTT-----

----ACCGTTTGAGTA----CTCTGAG----TGT--GATTT------------ACAAAAT

---CAAAATTAAAACTTTCAACAACGGATCTCTTGGCTCTAGCATCGATG-AAGAACGC-

AGCGAAATGCGATAAGTAATGCGAATTGCAGAATTCAGTGAATCATCGAATCTTTGAACG

CACATTGCGCCCGCCAGTATTCTGGCGGGCATGCCTGTCTGAGCGTCGTTTCGACCCTCG

CG-CCCGG--C-TTCT----GTCGGGGG--------------CGGTGTTGGGGAT-CGGC

C---------------A---CACCC----A-CTCCGGT-GGGAGG-CCGGCCCC-TAAAT

CCAGTGGCGACCACGCTGTAGCC-TCCCCTGCGTAGTACT---AAAACCACCTC--GCAG

GCGGAG--AGCGGTGCGGCC-C-GCCGTAAAACCCCC-A-ACT-TTTA-CAA-GG

>Gliomastix_masseei_AB540553_CBS79469 Gliomastix masseei genes for ITS1, 5.8S rRNA, ITS2 and 28S rRNA, partial sequence, strain: CBS 794.69.

ACT-GAGTT-----GC----AAAACTCCCAAACCC-ACTGTGAACC--TACC---ACT--

GTTGCTTCGGCGG-A-----TCCGCCCC-GGGCGCACCCT-TC-AGGGGG-TGT-GCCCC

GGAA-CCA--------GGCGCCCGCCGGGGG---ACC--GAAACCTCT-GTATCTT----

---CA--G-TTGAGTTA---CTCTGAG----TGT--GATTT------------CTAAAAT

---CAAAATTAAAACTTTCAACAACGGATCTCTTGGCTCTAGCATCGATG-AAGAACGC-

AGCGAAATGCGATAAGTAATGCGAATTGCAGAATTCAGTGAATCATCGAATCTTTGAACG

CACATGGCGCCCGCTAGTATTCTGGCGGGCATGCCTGTCTGAGCGTCGTTTCGACCCTCG

CC-CCCGG--C-GTCT----GTCGGGGG--------------CGGTGTTGGGGAT-CGGC

C---------------A--CCACCC-TTCA-CT------GGGCGG-CCGTCCCC-TAAAT

TCAGTGGCGACCACGCTGTAGCC-TCCCCTGCGTAGTACT---AAAACCACCTC--GCAG

GCGGAG--AGCGGTGCGGCC-C-GCCGTAAAACCCCCCAAACT-TTTA-CAA-GG

>Gliomastix_murorum_AB540557_NBRC31241 Gliomastix murorum genes for ITS1, 5.8S rRNA, ITS2 and 28S rRNA, partial sequence, strain: NBRC 31241.

ACC-GAGTT-----GC----AAAACTCCCAAACCC-ATTGTGAACCAATACC---ACT--

GTTGCTTCGGCGG-A-----CACGCCCC-GGGCGCACCTCCTC-AGGGGG-TGT-GCCCC

GGAA-CCA--------GGCGCCCGCCGGGGG---ACC--GAAACCTCT-GTATTT-----

----ACCATTCGAGTA----CTCTGAG----TGT--GATTT------------ACAAAAT

---CAAAATTAAAACTTTCAACAACGGATCTCTTGGCTCTAGCATCGATG-AAGAACGC-

AGCGAAATGCGATAAGTAATGCGAATTGCAGAATTCAGTGAATCATCGAATCTTTGAACG

CACATTGCGCCCGCCAGTATTCTGGCGGGCATGCCTGTCTGAGCGTCGTTTCGACCCTCG

CG-CCCGG--C-TTCT----GTCGGGGG--------------CGGTGTTGGGGAT-CGGC

C---------------A---CACCC-TTTA-CT------GGGCGG-CCGTCCCC-TAAAT

CCAGTGGCGACCACGCTGTAGCC-TCCCCTGCGTAGTACT---AAAACCACCTC--GCAG

GCGGAG--AGCGGTGCGGCC-C-GCCGTAAAACCCCCCA-ACT-TTTA-CAA-GG

>Acremonium_murorum_HQ637278, 627 bases, 0 checksum..

ACG-GAGAT-----GC------AACTCCC-AACCC-ATTGTGAACCAATACC---ACT--

GTTGCTTCGGCGG-A-----CACGCCCC-GGGCGCACCTCCTC-AGGGGG-TGT-GCCCC

GGAA-CCA--------GGCGCCCGCCGGGGG---ACC--GAAACCTCT-GTATTT-----

----ACCATTCGAGTA----CTCTGAG----TGT--GATTT------------ACAAAAT

---CAAAATTAAAACTTTCAACAACGGATCTCTTGGCTCTAGCATCGATG-AAGAACGC-

AGCGAAATGCGATAAGTAATGCGAATTGCAGAATTCAGTGAATCATCGAATCTTTGAACG

CACATTGCGCCCGCCAGTATTCTGGCGGGCATGCCTGTCTGAGCGTCGTTTCGACCCTCG

CG-CCCGG--C-TTCT----GTCGGGGG--------------CGGTGTTGGGGAT-CGGC

C---------------A---CACCC-TTTA-CT------GGGCGG-CCGTCCCC-TAAAT

CCAGTGGCGACCACGCTGTAGCC-TCCCCTGCGTAGTACT---AAAACCACCTC--GCAG

GCGGAG--AGCGGTGCGGCC-C-GCCGTAAAACCCCCCA-ACT-TTTA-CAA-GG

>Bionectria_grammicosporopsis_AF358255, 627 bases, 0 checksum..

-CC-GAGTTT----AC------AACTCCCAAACCC-A-TGTGAAC--ATACC--TATC--

GTTTCTTCGGCGGGA-----T-TGCCC-----------ATC-------------------

------CA--------GGCGCCTGCCG-AGGGG-ACC--TCAACTCTT-GTTTTA-----

-----C-AAT--AGTAT--CTTCTGAG----TAAC--ATTTT-----------T-AAA--

---TAAA-TAAAAACTTTCAACAACGGATCTCTTGGTTCTGGCATCGATG-AAGAACGC-

AGCGAAATGCGAAAAGTAATGTGAATTGCAGAATTCAGTGAACCATCGAATCTTTGAACG

CACATTGCGCCCGCCAGTATTCTGGCGGGCATGCCTGTCTGAGCGTCATTTCAACCCTCA

TG-CCCCC-------------CGGGGCG--------------TGGTGTTGGGGAT-CGGC

C--------------AAGG---CCC--------GC-AAGGGACGG-CCGGCCCC-TAAAT

CTAGTGGCGGACCCGTCGTGGCC-TCCCCTGCGAAGTAGT-----AATATT-CC--GCAT

-CGGAC--AGCGACGAGCCCCT-GCCGTTAAACCCCC-A-ACTTTAT--CAA-GG

>Bionectria_pseudostriata_AF358251, 627 bases, 0 checksum..

-CC-GAGTTT----AC------AACTCCCAAACCC-A-TGTGAAC--ATACC--TACT--

GTTGCTTCGGCGGGA-----T-CGCCCC-GGGCGCCTT-GC-----------GT-GCCCC

GGAC-CCA--------GGCGCCCGCCT-AGGA--ACC--TTAACTCTT-GTTTTATTTT-

------------GGAAT--CTTCTGAG----TAG---TTTTT-----------ACAAA--

---TAAA-TAAAAACTTTCAACAACGGATCTCTTGGTTCTGGCATCGATG-AAGAACGC-

AGCGAAATGCGATAAGTAATGTGAATTGCAGAATTCAGTGAATCATCGAATCTTTGAACG

CACATTGCGCCCGCCAGTATTCTGGCGGGCATGCCTGTCTGAGCGTCATTTCAACCCTCA

TG-CCCC--------------TAGGGCG--------------TGGTGTTGGGGAT-CGGC

C--------------AAGG---CCC--------GC-GAGGGACGG-CCGGCCCC-TAAAT

CTAGTGGCGGACCCGTCGTGGCC-TCCTCTGCGAAGTAGT-----GATATT-CC--GCAT

-CGGAG--AGCGACGAGCCCCT-GCCGTTAAACCCCC-A-ACT-TCT--CAA-GG

>Bionectria_ralfsii_AF358253, 627 bases, 0 checksum..

-CC-GAGTTT----AC------AACTCCCAAAACCCA-TGTGAAC--ATACC--TCTT--

GTTGCTTCGGCGGGA-----T-CGCCCC-GGGCGCCTT-GC-----------GT-GCCCC

GGAT-CCA--------GGCACCCGCCGGGGGA--CTT--TCAACTCTT-GTTTTTTCTT-

------------GGAAT--CTTCTGAG----TCG---TTTTT-----------ACAAA--

---TAAA-TAAAAACTTTCAACAACGGATCTCTTGGTTCTGGCATCGATG-AAGAACGC-

AGCGAAATGCGAAAAGTAATGTGAATTGCAGAATTCAGTGAATCATCGAATCTTTGAACG

CACATTGCGCCCGCCAGTATTCTGGCGGGCATGCCTGTCTGAGCGTCATTTCAACCCTCA

TG-CCCC--------------TAGGGCG--------------TGGTGTTGGGGAT-CGGC

C--------------AAGG---CCC--------GC-AAGGGACGG-CCGGCCCC-TAAAT

CTAGTGGCGGACCCATCGTGGCC-TCCCCTGCGAAGTAGT-----GATATT-CC--GCAT

-CGGAC--AGCGATGAGCCCCT-GCCGTTAAACCCCCCA-AACTT-T--CAA-GG

>Bionectria_compactiuscula_AF358247, 627 bases, 0 checksum..

-CC-GAGTTT----AC------AACTCCCAAACCC-A-TGTGAAC--ATACC--TATC--

GTTGCTTCGGCGGGA------CCGCCCC-GGGCGCATT-CC-----------GT-GCCCC

GGAT-CCA--------GGCGCCCGCCGGGGG---ACC--TTAACTCTT-GTTTTATTT--

------------AGAAT--CTTCTGAG----TAG---TTTTT-----------ACAAA--

---TAAA-TAAAAACTTTCAACAACGGATCTCTTGGTTCTGGCATCGATG-AAGAACGC-

AGCGAAATGCGAAAAGTAATGTGAATTGCAGAATTCAGTGAATCATCGAATCTTTGAACG

CACATTGCGCCCGCCAGTATTCTGGCGGGCATGCCTGTCTGAGCGTCATTTCAACCCTCA

TG-CCCC--------------TAGGGCG--------------TGGTGTTGGGGAT-CGGC

C--------------AAAG---CCC--------GC-AAGGGACGG-CCGGCCCC-TAAAT

CTAGTGGCGGACCCGTCGTGGCC-TCCTCTGCGAAGTAGT-----AATATT-CC--GCAT

-CGGAC--AGCGACGAGCCCCT-GCCGTTAAACCCCC-A-ACTTTCT---AA-GG

>Bionectria_zelandiaenovae_AF358229, 627 bases, 0 checksum..

-CC-GAGTTT----AC------AACTCCCAAACCC-A-TGTGAAC--ATACC--TATT--

GTTGCTTCGGCGGGA-----T-CGCCCC-AGGCGCCTT--------GT----GT-GCCCT

GGA--CCC--------GGCGCCCGCCT-AGGA--ACC--TTAACTCTT-GTTTTATTTT-

-------------GAAT--CTTCTGAG----TAG---TTTTT-----------ACAAA--

---TAAA-TAAAAACTTTCAACAACGGATCTCTTGGTTCTGGCATCGATG-AAGAACGC-

AGCGAAATGCGATAAGTAATGTGAATTGCAGAATTCAGTGAATCATCGAATCTTTGAACG

CACATTGCGCCCGCCAGTATTCTGGCGGGCATGCCTGTCTGAGCGTCATTTCAACCCTCA

TG-CCCC--------------TAGGGCG--------------TGGTGTTGGGGAT-CGGC

C--------------AAAG---CCC--------GT-AAGGGACGG-CCGGCCCC-TAAAT

CTAGTGGCGGACCCGTCGTGGCC-TCCCCTGCGAAGTAGT-----GATATT-CC--GCAT

-CGGAG--AGCGATGAGCCCCA-GCCGTTAAACCCCC-A-ACTTTC---CAA-GG

>Bionectria_samuelsii_AF210689, 627 bases, 0 checksum..

-TC-GAGTTT----AC------AACTCCCAAACCC-A-TGTGAAC--ATACC--TATC--

GTTGCTTCGGCGGGA-----T-CGCCCC-GGGCGCCTT--------GT----GT-GCCCC

GGAT-CCA--------GGCGCCCGCCAGAGG---ACT--TTAACTCTT-GTTTTATTTT-

T------------GAAT--CATCTGAG----TAG---TTTTTT----------ACAAA--

---TAAA-TAAAAACTTTCAACAACGGATCTCTTGGTTCTGGCATCGATG-AAGAACGC-

AGCGAAATGCGAAAAGTAATGTGAATTGCAGAATTCAGTGAATCATCGAATCTTTGAACG

CACATTGCGCCCGCCAGTATTCTGGCGGGCATGCCTGTCTGAGCGTCATTTCAACCCTCA

TG-CCCC--------------TAGGGCG--------------TGGTGTTGGGGAT-CGGC

C--------------AAGG---CCC--------GC-AAGGGACGG-CCGGCCCC-TAAAT

CTAGTGGCGGACCCGTCGTGGCC-TCCTCTGCGAAGTAGT-----AATATT-CC--GCAT

-CGGAG--AGCGACGAGCCCCT-GCCGTTAAACCCCC-A-ACTTT--A-CAA-GG

>Bionectria_solani_AF210687, 627 bases, 0 checksum..

-CC-GAGTTT----AC------AACTCCCAAACCC-A-TGTGAAC--ATACC--TACT--

GTTGCTTCGGCGGGA-----T-TGCCCC-GGGCACCTC--------GT----GT-GCCCC

GGAT--CA--------GGCGCCCGCCT-AGGA--AAC--TTAACTCTT-GTTTTATTTT-

------------GGAAT--CTTCTGAG----TAG---TTTTT-----------ACAAA--

---TAAA-TAAAAACTTTCAACAACGGATCTCTTGGTTCTGGCATCGATG-AAGAACGC-

AGCGAAATGCGATAAGTAATGTGAATTGCAGAATTCAGTGAATCATCGAATCTTTGAACG

CACATTGCGCCCGCCAGTATTCTGGCGGGCATGCCTGTCTGAGCGTCATTTCAACCCTCA

TG-CCCC--------------TAGGGCG--------------TGGTGTTGGGGAT-CGGC

C--------------AAAG---CCC--------GC-GAGGGACGG-CCGGCCCC-TAAAT

CTAGTGGCGGACCCGTCGTGGCC-TCCTCTGCGAAGTAGT-----AATATT-CC--GCAT

-CGGAC--AGCGACGAGCCCCT-GCCGTTAAACCCCC-A-ACTTTCT---AA-GG


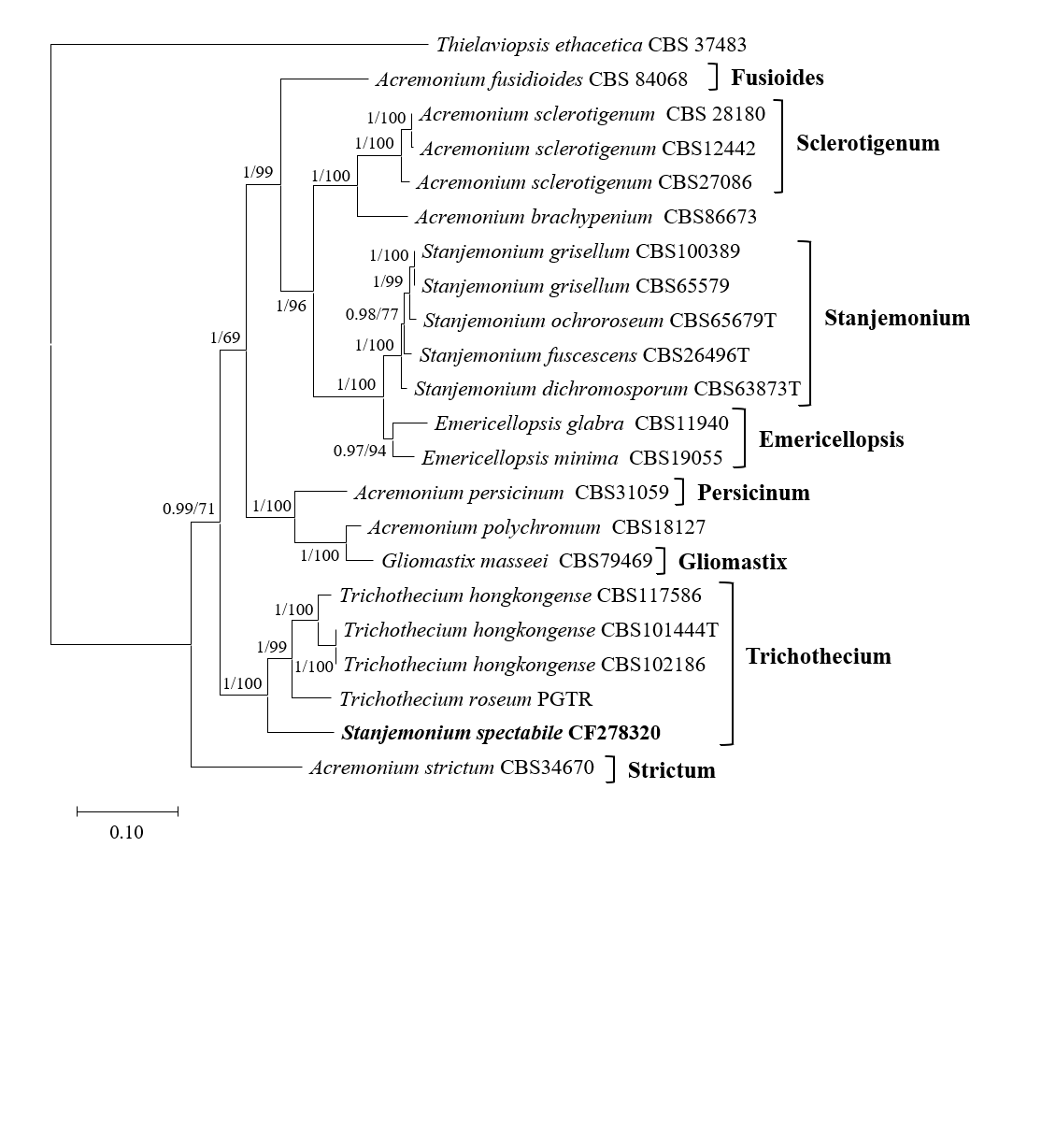


Figure S.22 Phylogenetic tree inferred from Bayesian and Maximum Likelihood (ML) analyses based on a concatenated alignment of ITS, LSU, tef1, and rpb2 sequences. Probability values/ML bootstrap values are indicated respectively on the branches.

Table S.5 List of strains used in the multi-locus ITS, LSU, *tef1*, and *rpb2* phylogenetic study, with their accession numbers.

| ***Taxa*** | **Strain** | **ITS GenBank accession Nº** | **References** | **LSU GenBank accession Nº** | **References** | **TEF1 GenBank accession Nº** | **References** | **RPB2 GenBank accession Nº** | **References** |
| --- | --- | --- | --- | --- | --- | --- | --- | --- | --- |
| *Acremonium brachypenium* | CBS 866.73 | AB540570 | Kiyuna (2011) | OQ055354 | Hou (2023) | OQ470740 | Hou (2023) | OQ453837 | Hou (2023) |
| *Acremonium fusidioides* | CBS 840.68 | FN706542 | Perdomo (2011) | HQ232039 | Summerbell (2011) | OQ471272 | Hou (2023) | OQ454339 | Hou (2023) |
| *Acremonium persicinum* | CBS 310.59 | FN706554 | Perdomo (2011) | OQ430172 | Hou (2023) | OQ471251 | Hou (2023) | OQ454318 | Hou (2023) |
| *Acremonium polychromum* | CBS 181.27 | AB540567 | Kiyuna (2011) | OQ055528 | Hou (2023) | OQ470931 | Hou (2023) | OQ454020 | Hou (2023) |
| *Acremonium sclerotigenum* | CBS 281.80 | FN706549 | Perdomo (2011) | HQ232128 | Summerbell (2011) | PV414821 | Zhao (2026) | PV458242 | Zhao (2026) |
| *Acremonium sclerotigenum* | CBS 124.42 | FN706552 | Perdomo (2011) | OQ055363 | Hou (2023) | OQ470750 | Hou (2023) | OQ453846 | Hou (2023) |
| *Acremonium sclerotigenum* | CBS 270.86 | FN706551 | Perdomo (2011) | OQ055374 | Hou (2023) | OQ470760 | Hou (2023) | OQ453857 | Hou (2023) |
| *Acremonium strictum* | CBS 346.70 | GQ376096 | Wicht (2012) | HQ232141 | Summerbell (2011) | OQ471184 | Hou (2023) | OQ454252 | Hou (2023) |
| *Emericellopsis glabra* | CBS 119.40 | AY632657 | Zuccaro (2004) | MH867552 | Vu (2019) | OQ470872 | Hou (2023) | OQ453965 | Hou (2023) |
| *Emericellopsis minima* | CBS 190.55 | AY632669 | Zuccaro (2004) | OQ055482 | Hou (2023) | OQ470876 | Hou (2023) | KC999031 | Grum-Grzhimaylo (2013) |
| *Gliomastix masseei* | CBS 794.69 | AB540553 | Kiyuna (2011) | OQ055510 | Hou (2023) | OQ470911 | Hou (2023) | OQ454000 | Hou (2023) |
| *Stanjemonium dichromosporum* | CBS 638.73 | NR_077122 | Schoch (2014) | OQ430115 | Hou (2023) | OQ471197 | Hou (2023) | OQ454265 | Hou (2023) |
| *Stanjemonium fuscescens* | CBS 264.96 | NR_160198 | Vu (2019) | OQ430116 | Hou (2023) | OQ471198 | Hou (2023) | OQ454266 | Hou (2023) |
| *Stanjemonium grisellum* | CBS 100389 | PV272738 | Zhao (2025) | PV272960 | Zhao (2025) | PV273518 | Zhao (2025) | PV273317 | Zhao (2025) |
| *Stanjemonium grisellum* | CBS 655.79 | NR_156501 | Zuccaro (2004) | OQ430117 | Hou (2023) | OQ471199 | Hou (2023) | OQ454267 | Hou (2023) |
| *Stanjemonium ochroroseum* | CBS 656.79 | NR_137145 | Zuccaro (2004) | OQ430118 | Hou (2023) | OQ471200 | Hou (2023) | OQ454268 | Hou (2023) |
| *Stanjemonium spectabile* | CF-278320 | PX069749 | Present study | PX069749 | Present study | PZ462790 | Present study | PZ464162 | Present study |
| *Thielaviopsis ethacetica* | CBS 374.83 | JX518329 | Mbenoun (2014) | - | - | JX518297 | Mbenoun (2014) | DQ368641 | Tang (2006) |
| *Trichothecium hongkongense* | CBS 117586 | OQ429888 | Hou (2023) | OQ430140 | Hou (2023) | OQ471220 | Hou (2023) | OQ454289 | Hou (2023) |
| *Trichothecium hongkongense* | CBS 101444 | OQ429887 | Hou (2023) | OQ430139 | Hou (2023) | OQ471219 | Hou (2023) | OQ454288 | Hou (2023) |
| *Trichothecium hongkongense* | CBS 102186 | OQ429886 | Hou (2023) | OQ430138 | Hou (2023) | OQ471218 | Hou (2023) | OQ454287 | Hou (2023) |
| *Trichothecium roseum* | PGTR-1 | OQ996599 | Zhang (2021) | OQ996628 | Zhang (2021) | OR101076 | Zhang (2021) | OR100959 | Zhang (2021) |

Table S.6 Alignment used in the multi-locus (ITS, LSU, *rpb2*, *tef1*) phylogenetic study.

>Thielaviopsis_ethacetica_CBS37483 ...

-TC-GAGTTTTT--------AACTCTTAAACCATTTGTGAAC--TTACC--TTCTGGCTG

CTTTGGCAGG--TCCTT---CGGGATTT--------------------------------

-GCCGGTAGC------ACAAA-CAAACTCTTTATATTT--CTAGAGAATTATTCATTGCT

GAGTGG--CATTA---------ACTAAA-TAAG-TTAAAACTTTCAACAACGGATCTCTT

GGCTCTAGCATCGATGAAGAACGCAGCGAAATGCGATACGTAATGTGAATTGCAGAACTC

AGTGAATCATCGAATCTTTGAACGCACATTGCACCTGGCAGCATTCTGCCAGGTATGCCT

GTCCGAGCGTCATTTCACCACTCAAGCTCTGC----------------------------

----TTGGCGTTGGAGGACCCGC-------------------------GTTTGCGGGCCG

CCGAAATGAATCGGCTGTTATACTTGCAGC-TTCCCTGCGTAGTAATTT--T-GTGTT--

ACGCTTTGAAACTCTTGTACTAC-ATGCCGTTAAACCCATC-AATTTTTT----------

------------------------------------------------------------

------------------------------------------------------------

------------------------------------------------------------

------------------------------------------------------------

------------------------------------------------------------

------------------------------------------------------------

------------------------------------------------------------

------------------------------------------------------------

------------------------------------------------------------

--------------------CGCCAGCTTCACAACACTCACTGGGGTCTGGTGTGTCCCG

CTGAGACTCCCGAAGGTCAGGCCTGTGGTCTGGTCAAGAATCTTTCGCTCATGTGCTTCG

TCAGTGTCGGAACGCCGTCTGATCCTATAATAGAATTTATGATCAACCGCGGCATGGAGA

TCATTGAAGAATATGAACCTTTGCGCTACCCCAACGCCACCAAGGTGTTTGTCAACGGTG

TCTGGGTCGGTGTTCACCAGGATCCCAGACACCTGGTCACTGAGATGATGGAGACCCGTC

GTCGTTCGTACCTCCCCAACGAGGTTTCTCTTGTTCGCGATATCAGAGATCGCGAATTCA

AGATTTTCTCCGATGCTGGGCGTGTCATGCGACCCGTGTTTGTTGTCCAGCGTGATGACG

ACC-CTGCCCGTGGTCTTCAGGCTGGCCAGCTGGTGCTCACCAAGGAGCACGTCAATGCC

ATTCGCCAGGAGCAGG-AGGCTACCAACGGC---------GAAACTCGCG------ACTT

TGGATGGAACTGGCTCGTCAACAAGGGTGTTATTGAATACCTGGATGCCGAAGAAGAGGA

AACCACCATGATCTGCATGACGCCTGAAGATCTTGATACGTATCGCGCCAAGAAGGCC--

-------------------------GGTGTTGAGCCTGAGCCG---------GAACCCAT

GACCGAGGCTGATATCAACAAGCGTCTCAAAACCAACATCAAGCCCACAACACACATGTA

CACTCACTGCGAGATCCACCCCAGCATGA------GAGAATAAGTCTC-------CCCTA

TCCCCAGTTCTACACTTG------AAAAACTTTTTG--GCCCATGGTCTGTTTTTTGCGG

CTACACTTGCCCACAATTTGTGCGCGAGTGGACGTCAAAGAAAACTTTTTTACCCCTCCC

TTGATGTGGGGCACA-TCGCACGCTTTGTGCGTGTGTCC---TGTCAAGACCCACAAATA

ACAATTTGTGAGGGGTAGACCCTGGTCTATTGCTCCTTCCCTACGCATGCATTCATTGCA

TGCATATTTTT---CAAATTCAC--GTACTGACCTACTTTGCACAG--GAGGCCGCTGAG

CTCGGTAAGGGTTCCTT---CAAGTACGCCTGGGTTCTTGACAAGCTCAAGGCCGAGCGT

GAGCGTGGTATCACCATCGATATTGCCCTGTGGAAGTTCGAGACCCCTAAGTACTACGTC

ACTGTCATCGGTA----TGTTGTCTTCGCCTACGTCGTTA--TCCTTGACATTCCACTAA

CAATTGATGTTTACAGATGCCCCCGGTCACAGA---GATTTCA---TCAAGAACATG---

ATCACTGGTACCTCGCAGGCTGACTGCGCT----ATCCTGATCAT-----TGCTGCCGGT

ACCGGTGAGTTCGAGGCTGGTATCTCCAAGGATGGCCAGACCCGTGAGCACGCT--CTGC

TG-GCTTTCACCCTGGG-TGTCAAGCAGCTGATTGTTGCCATCAACAAGATGGACACCAC

CAAGTGGTCTGAGGCCCGTTACCAGGAGATCATCAAGGAGACCTCCTCCTTCATCAAG--

--------

>Acremonium_fusidioides_FN706542_CBS84068 Acremonium fusidioides ITS1, 5.8S rRNA gene, ITS2 and 28S rRNA gene (partial), culture collection CBS:840.68......

------GT-----A------AACTCCCTTACC-TTTGTGAAC--ATACC-----T-GTTG

CTTCGGCGG---TCCTCAC-CGGC-C----GC------------------C-GAA-----

-GGC---------------CT--ATATTCTT-GAATTT---AC----A-TGAA--TTTCT

GAGTAT--CA-AA--C------A-AAAA-TAAA-TAAAAACTTTCAGCAACGGATCTCTT

GGCTCTGGCATCGATGAAGAACGCAGCGAAATGCGATAAGTAATGTGAATTGCAGAATTC

AGTGAATCATCGAATCTTTGAACGCACATTGCGCCCACCAGTACTCTGGTGGGCATGCCT

GTCCGAGCGTCATTTCAACCCTCAGGGCCC-G----TTC-----GCGGG-----------

--ACCTGGTGTTGGGGAT-CGGCC-------CCA--------------CCGG-CCGGCCC

C-GAAATACAGTGGCGGCACACCCGCGACC-TCCTCTGCGTAGTAGC--AAT-GCCTC--

GCAGCTGGAT--AGCGGTTGCGCCTCGCCGTAAAACCCCCC--ACT-TCT--CAAAAGTA

ACGGCGA-GTGAAGCGGCAACAGCTCAAATTTTGAAATCT-GGC--TCT-CGGGCCCG-A

GTTGTAATTTGTAGAGGATGTTTCTGGCGACGTGTCTTCCGAGTTCCCTGGCAACGGGAC

GCCATAGAGGGTGAGAGCCCCGTCCGGTCGTACGCC-T-AGCCTCTGTGAAACTCCTTCG

ACGAGTCGAGTAGTTTGGGAATGCTGCTCAAAATGGGAGGTATACGTCTTCTAAAGCTAA

ATACCGGCCAGAGACCGATAGCGCACAAGTAGAGTGATCGAAAGATGAAAAGCACTTTGA

AAAGAGGGTTAAATAGTACGTGAAATTGCTGAAAGGGAAGCGCTCATGACCAGACTTGGG

CTCGGTGAATCATCCGGCGTTCTCGCCGGTGCACTTT-G-CCGGCCCAGGCCAGCATCAG

CTCGCCTCGGGGGACAAAGGCTTCGGGAATGTAGCTCC-CTCGT--GGAGTGTTATAGAC

CGTTGCGTAATACCCTGGGGCGGGCTGAGGTACGCG-TTCTGCAAGGATGCTGGCGTAAT

GGTCATCAGTGACCCGTCTTCGACAGCTTCACAACACCCACTGGGGCTTGGTTTGTCCCG

CAGAAACACCCGAAGGCCAGGCTTGTGGTCTGGTCAAGAACTTGTCTCTGATGTGCTACG

TTTCCGTTGGCTCTCCCTCTGAGCCTCTGCTGTCCTTCATGATTAGCAGAGGCATGGAAC

TTATCGAAGAGTACGACCCGTTACGATTCCCTCACGCTACCAAGATCTTCATTAATGGTA

GCTGGGTGGGCATTCATCAAGAACCTAAACAGCTTGTCGATCATTTGGTGGCGCTGCGTC

GCTCTGCTCGAATGGCTCACGAGGTGTCTCTCGTTCGCGACATTCGTGACCGTGAGTTCA

AATTCTTCTCTGATGCCGGTCGCGTGATGCGTCCCGTCTTCACTGTACGTCAAGAAGATG

GTG-AGATTGGTGACCCACCCAAGGGTTCCCTGGTCCTGACCAAAGATATCATAAACAAC

ATCGATGAAGATTACT-GGCACTCTGATAAGGA-------AGAATACTTG------A--G

AGGCTGGGATAAGCTTGTCGGTATTGGTTGCGTTGAATACCTAGACGCAGAGGAAGAGGA

GACAGCCATGATTTGCATGACTCCTGAGGATCTCGAGTCCTATCGAGTGCAACGA-----

----------------------------CTTGGTTACGAGGCTCCC------GATGCCTA

CGAGGAGCTGGAACCCAACAAGCGCTTGAAGACGAAGACCAATCCGACGACGCACATGTA

TACCCACTGTGAGATTCATCCTAGCATGATCCAAGGATGGCCAGACTCGTGAGCACGCTC

TCCTCGCCTACACTCTCGGTGTCCGACAGCTCATTGTCGCCATCAACAAGATGGACACTG

CT-CAGTGGAAGGAGGCCCGTTACCTTGAGATTATCAAGGAGA--CTTCCAACTTCATCA

AGAAGGTCGGCTACAACCCCAAGACTGTTGCCTTCGTCCCCATCTCTGGTTTCAACGGTG

ACAACATGCTGGAGC----CTTCCACCAACTGCCCCTGGTACAAGGGCTGGGAGAAGGAG

GGTAAGGGTGGTGCCAAGGTCACTGGCAAGACCATCCTCGAGGCCATTGACTCCATCGAG

CCCCCCAAGCGTCCTCTCGACAAGCCCCTCCGTCTTCCCCTCCAGGATGTCTACAAGATC

GGTGGTATTGGAACGGTGCCTGTCGGCCGTATCGAGACTGGTATCATCAAGCCCGGTATG

GTCGTCACCTTCGCTCCCGCCAACGTCACCACTGAAGTCAAGTCCGTCGAGATGCACCAC

GAGCAGCTCACCGAGGGTCTCCCCGGTGACAACGTTGGTTTCAACGTGAAGAACGTCTCC

GTCAAGGATATCCGCCGTGGTAACGTTGCTTCTGACTCCAAGAACGACCCCGCCTCCGGC

GCCGCTACCTTCAACGCCCAGGTCATTGTCCTGAACCACCCTGGTCAGGTTGGCCCTGGC

TACGCCCCCGTTCTCGACTGCCACACTGCCCAC-ATTGCTTGCAAGTTCACCGAGATTCT

TGAGAAGATCGACCGCCGTACTGGTAAGTCGGTTGAGAACAACCCCAAGTTCATCAAGTC

CGGTGACT

>Acremonium_sclerotigenum_FN706549_CBS28180 Acremonium sclerotigenum 18S rRNA gene (partial), ITS1, 5.8S rRNA gene, ITS2 and 28S rRNA gene (partial), culture collection CBS:281.80......

ACC-GAGTGT---AA----AAACTCCCAAACC-ATTGTGAAC--CTACC---ACT-GTTG

CTTCGGCGGC-CTCGCCCC-GGGCGCGTTCGC-----------GC--GGCCCGGAC-CCA

-GGCGTCCGCCGGAGG---CTCCAAACTCTT-GTCTTT--------TAGTGTA--TTTCT

GAGTGG--CATAA-GC--------AAA--TAAA-TCAAAACTTTCAGCAACGGATCTCTT

GGTTCTGGCATCGATGAAGAACGCAGCAAAATGCGATAAGTAATGTGAATTGCAGAATTC

AGTGAATCATCGAATCTTTGAACGCACATTGCGCCCGCCAGTATTCTGGCGGGCATGCCT

GTCTGAGCGTCATTTCAACCCTCAGGACCC-G----TTC-----GCGGG-----------

--ACCTGGCGTTGGGGATCA-GCC----TGCCC------CTGG---CGGCGG-CTGGCCC

T-GAAATCCAGTGGCGGTTCCCTCGCGAAC-TCCTCCGTGCAGTAAT-TAA--ACCTCTC

GCGGCAGGAT--AGCGGTTGAACCACGCCGTTA-ACCCCCC--ACT-TCT--CAA-AGTA

ACGGCGA-GTGAAGCGGCAACAGCTCAAATT-TGAAATCT-GGCC-TC-AC-GGTCCG-A

ATTGTAATTTGTAGAGGATGTTTCTGGCGACGTGTCTTCCGAGTTCCCTGG-AACGGGAC

GCCATAGAGGGTGAGAGCCCCGTCCGGTCGTACACC-T-AGCCTCTGTGAAACTCCTTCG

ACGAGTCGAGTAGTTTGGGAATGCTGCTCTAAATGGGAGGTATACGTCTTCTAAAGCTAA

ATACCGGCCAGAGACCGATAGCGCACAAGTAGAGTGATCGAAAGATGAAAAGCACTTTGA

AAAGAGGGTTAAATAGTACGTGAAATTGCTGAAAGGGAAGCGCTTATGACCAGACTTGGG

CTCGGTGAATCATCCGGCGTTCTCGCCGGTGCACTTT-G-CCGTCCCAGGCCAGCATCAG

TTCGCGCCGGGGGATAAAGGTTTCGGGAATGTAGCTCC-CTC---GGGAGTGTTATAGCC

CGTTGCGTAATACCCTGGCGTGGACTGAGGTCCGCGC-TCTGCAAGGATGCTGGCGTAAT

GGTCATCAGTGACCCGTCTTCGGCAGCTACACAACACCCATTGGGGTCTTGTGTGTCCTG

CCGAGACCCCCGAGGGTCAAGCTTGTGGTTTGGTGAAGAACCTGTCACTGATGTGTCACG

TTTCCGTTGGCACACCCAGCGAACCTCTCTACGGATACTTCATCAACCGTGGCATGGAAG

TGCTCGAAGAGTACGAACCCCAGCGGTTCCCCAACGCCACCAAGGTGTTCATCAACGGTG

CCTGGGTCGGTGTGCATACGAGCCCGAAAGATCTCGTAGATAGTATCATGCATCTGCGGC

GCTATGGTGACCTTAACCACGAAGCTTCCGTCATCCGCGACATTCGGGATCGAGAGTTCA

GAGTCGTCACAGATGCTGGTCGTGTCATGCGCCCAGTATTCACAGTGCAGCAAGAAGACA

----AGCTAGACGGGCCCGAAAAGGGGTCGTTATGCATGACCAAGGAGCATCTTGCCGGG

TTGGATGACTGGCATC-TGGTCAACGAGGAGAG-------GGAGGAGATGG---CCA--C

GGGCTGGGAATATCTCGTGAAGAGTGGGTGTATCGAGTACCTGGACGCCGAAGAAGAAGA

GACGGCAATGATCTGCATGACCCCCGAAGATTTGGAGTCTTACCGCAAGGAGAAGTAC--

----------------------------CTGGATCAGAAACCCCAG------GAACACAA

CGTGGAAGCCGAGCCCAACAAGCGACTCAAGACAAAGACTAACCCAACGACGCACATGTA

CACCCACTGCGAGATTCATCCCAGTATGATCCAAGGATGGCCAGACCCGTGAGCACGCTC

TGCTCGCCTACACCCTGGGTGTGCGTCAGCTCATTGTTGCCATCAACAAGATGGACACCA

CC-CAGTGGTCTGAGGCCCGTTACCAGGAGATCATCAAGGAGA--CTTCCAACTTCATCA

AGAAGGTCGGCTTCAACCCCAAGACTGTTGCCTTCGTCCCCATCTCTGGTTTCCACGGCG

ACAACATGTTGGCTG----CTTCCACCAACTGCCCCTGGTACAAGGGCTGGGAGAAGGAG

GGCAAGGGTGGCGCCAAGGTCACCGGCAAGACCCTGCTTGAGGCCATCGACTCCGTCGAG

CCCCCCAAGCGTCCCAACGACAAGCCCCTCCGTCTTCCTCTCCAGGATGTCTACAAGATC

GGTGGTATTGGCACAGTGCCCGTCGGCCGTATCGAGACCGGTATCCTCAAGCCCGGCATG

GTCGTCACCTTCGCTCCCGCCAACGTCACCACTGAAGTCAAGTCCGTCGAGATGCACCAC

GAGCAGCTGCCCGAGGGTCTCCCCGGTGACAACGTTGGCTTCAACGTGAAGAACGTTTCC

GTTAAGGATATCCGCCGTGGCAACGTCGCCTCTGACTCCAAGAACGACCCCGCCATGGGC

GCTGCTTCCTTCAACGCCCAGGTCATTGTCCTGAACCACCCTGGTCAGGTCGGTGCCGGT

TACGCTCCCGTCCTCGACTGCCACACCGCCCAC-ATTGCTTGCAAGTTCTCCGAGATCCT

CGAGAAGATCGACCGCCGTACCGGTAAGTCGGTTG-------------------------

--------

>Acremonium_sclerotigenum_FN706552_CBS12442 Acremonium sclerotigenum ITS1, 5.8S rRNA gene and ITS2, culture collection CBS:124.42......

ACC-GAGTGT---AA----AAACTCCCAAACC-ATTGTGAAC--CTACC---ACT-GTTG

CTTCGGCGGC-CTCGCCCC-GGGCGCGTTCGC-----------GC--GGCCCGGAC-CCA

-GGCGTCCGCCGGAGG---CTCCAAACTCTT-GTCTTT--------TAGTGTA--TTTCT

GAGTGG--CATAA-GC--------AAA--TAAA-TCAAAACTTTCAGCAACGGATCTCTT

GGTTCTGGCATCGATGAAGAACGCAGCAAAATGCGATAAGTAATGTGAATTGCAGAATTC

AGTGAATCATCGAATCTTTGAACGCACATTGCGCCCGCCAGTATTCTGGCGGGCATGCCT

GTCTGAGCGTCATTTCAACCCTCAGGACCC-G----TTC-----GCGGG-----------

--ACCTGGCGTTGGGGATCA-GCC----TGCCC------CTGG---CGGCGG-CTGGCCC

T-GAAATCCAGTGGCGGTTCCCTCGCGAAC-TCCTCCGTGCAGTAAT-TAA--ACCTCTC

GCGGCAGGAT--AGCGGTTGAACCACGCCGTTAAACCCCCC--ACT-TCT--CAA-AGTA

ACGGCGA-GTGAAGCGGCAACAGCTCAAATT-TGAAATCT-GGCC-TC-AC-GGTCCG-A

ATTGTAATTTGTAGAGGATGTTTCTGGCGACGTGTCTTCCGAGTTCCCTGG-AACGGGAC

GCCATAGAGGGTGAGAGCCCCGTCCGGTCGTACACC-T-AGCCTCTGTGAAACTCCTTCG

ACGAGTCGAGTAGTTTGGGAATGCTGCTCTAAATGGGAGGTATACGTCTTCTAAAGCTAA

ATACCGGCCAGAGACCGATAGCGCACAAGTAGAGTGATCGAAAGATGAAAAGCACTTTGA

AAAGAGGGTTAAATAGTACGTGAAATTGCTGAAAGGGAAGCGCTTATGACCAGACTTGGG

CTCGGTGAATCATCCGGCGTTCTCGCCGGTGCACTTT-G-CCGTCCCAGGCCAGCATCAG

TTCGCGCCGGGGGATAAAGGTTTCGGGAATGTAGCTCC-CTC---GGGAGTGTTATAGCC

CGTTGCGTAATACCCTGGCGTGGACTGAGGTCCGCGC-TCTGCAAGGATGCTGGCGTAAT

GGTCATCAGTGACCCGTCTTCGGCAGCCTCACAACACCCATTGGGGTCTTGTGTGTCCTG

CCGAGACCCCCGAGGGTCAAGCTTGTGGTTTGGTGAAGAACCTGTCACTGATGTGTCACG

TTTCCGTTGGCACACCCAGCGAACCTCTCTACGGATACTTCATCAACCGTGGCATGGAAG

TGCTCGAAGAGTACGAACCCCAGCGGTTCCCCAACGCCACCAAGGTGTTCATCAACGGTG

CCTGGGTCGGTGTGCATACGAGCCCGAAAGATCTCGTAGATAGTATCATGCATCTGCGGC

GCTATGGTGACCTTAACCACGAAGCTTCCGTCATCCGCGACATTCGGGATCGAGAGTTCA

GAGTCGTCACAGATGCTGGTCGTGTCATGCGCCCAGTATTCACAGTGCAGCAAGAAGACA

----AGCTAGACGGGCCCGAAAAGGGGTCGTTATGCATGACCAAGGAGCATCTTGCCGGG

TTGGATGACTGGCATC-TGGTCAACGAGGAGAG-------GGAGGAGATGG---CCA--C

GGGCTGGGAATATCTCGTGAAGAGTGGGTGTATCGAGTACCTGGACGCCGAAGAAGAAGA

GACGGCAATGATCTGCATGACCCCCGAAGATTTGGAGTCTTACCGCAAGGAGAAGTAC--

----------------------------CTGGATCAGAAACCCCAG------GAACACAA

CGTGGAAGCCGAGCCCAACAAGCGACTCAAGACAAAGACTAACCCAACGACGCACATGTA

CACCCACTGCGAGATTCATCCC-------TCCAAGGATGGCCAGACCCGTGAGCACGCTC

TGCTCGCCTACACCCTGGGTGTGCGTCAGCTCATTGTTGCCATCAACAAGATGGACACCA

CC-CAGTGGTCTGAGGCCCGTTACCAGGAAATCATCAAGGAGA--CTTCCAACTTCATCA

AGAAGGTCGGCTTCAACCCCAAGACTGTTGCCTTCGTCCCCATCTCTGGTTTCCACGGCG

ACAACATGTTGGCTG----CTTCCACCAACTGCCCCTGGTACAAGGGCTGGGAGAAGGAG

GGCAAGGGTGGCGCCAAGGTCACCGGCAAGACCCTGCTTGAGGCCATCGACTCCGTCGAG

CCCCCCAAGCGTCCCAACGACAAGCCCCTCCGTCTTCCTCTCCAGGATGTCTACAAGATC

GGTGGTATTGGCACAGTGCCCGTCGGCCGTATCGAGACCGGTATCCTCAAGCCCGGCATG

GTCGTCACCTTCGCTCCCGCCAACGTCACCACTGAAGTCAAGTCCGTCGAGATGCACCAC

GAGCAGCTGCCCGAGGGTCTCCCCGGTGACAACGTTGGCTTCAACGTGAAGAACGTTTCC

GTTAAGGATATCCGCCGTGGCAACGTCGCCTCTGACTCCAAGAACGACCCCGCCATGGGC

GCTGCTTCCTTCAACGCCCAGGTCATTGTCCTGAACCACCCTGGTCAGGTCGGTGCCGGT

TACGCTCCCGTCCTCGACTGCCACACCGCCCAC-ATTGCTTGCAAGTTCTCCGAGATCCT

CGAGAAGATCGACCGCCGTACCGGTAAGTCGGTTGAGAACAACCCCAAGTTCATCAAGTC

CGGCGACT

>Acremonium_sclerotigenum_FN706551_CBS27086 Acremonium sclerotigenum ITS1, 5.8S rRNA gene and ITS2, culture collection CBS:270.86......

--C-GAGTG----AA----AAACTCCCAAACC-ATTGTGAAC--TTACC---ACT-GTTG

CTTCGGCGGC-CTCGCCCC-GGGCGCGTTCGC-----------GC--GGCCCGGAC-CCA

-GGCGTCCGCCGGAGG---CTCCAAACTCTT-GTCTTT--------TAGTGTA--TTTCT

GAGTGG--CATAA-GC--------AAA--TAAA-TCAAAACTTTCAGCAACGGATCTCTT

GGTTCTGGCATCGATGAAGAACGCAGCAAAATGCGATAAGTAATGTGAATTGCAGAATTC

AGTGAATCATCGAATCTTTGAACGCACATTGCGCCCGCCAGTATTCTGGCGGGCATGCCT

GTCTGAGCGTCATTTCAACCCTCAGGACCC-G----TTC-----GCGGG-----------

--ACCTGGCGTTGGGGATCA-GCC----TGCCC------CTGG---CGGCGG-CTGGCCC

T-GAAATCCAGTGGCGGTTCCCTCGCGAAC-TCCTCCGTGCAGTAAT-TAA--ACCTCTC

GCGGCAGGAT--AGCGGTTGAACCACGCCGTTAAACCCCCC--ACT-TCT--CAA-AGTA

ACGGCGA-GTGAAGCGGCAACAGCTCAAATT-TGAAATCT-GGCC-TC-AC-GGTCCG-A

ATTGTAATTTGTAGAGGATGTTTCTGGCGACGTGTCTTCCGAGTTCCCTGG-AACGGGAC

GCCATAGAGGGTGAGAGCCCCGTCCGGTCGTACACC-T-AGCCTCTGTGAAACTCCTTCG

ACGAGTCGAGTAGTTTGGGAATGCTGCTCTAAATGGGAGGTATACGTCTTCTAAAGCTAA

ATACCGGCCAGAGACCGATAGCGCACAAGTAGAGTGATCGAAAGATGAAAAGCACTTTGA

AAAGAGGGTTAAATAGTACGTGAAATTGCTGAAAGGGAAGCGCTTATGACCAGACTTGGG

CTCGGTGAATCATCCGGCGTTCTCGCCGGTGCACTTT-G-CCGTCCCAGGCCAGCATCAG

TTCGCGCCGGGGGATAAAGGTTTCGGGAATGTAGCTCC-TTC---GGGAGTGTTATAGCC

CGTTGCGTAATACC-TGGCGTGGACTGAGGTCCGCGC-TCTGCAAGGATGCTGGCGTAAT

GGTCATCAGTGACCCGTCTTCGGCAGCTACACAACACGCATTGGGGTCTTGTGTGTCCCG

CCGAGACCCCCGAGGGTCAGGCTTGTGGTTTGGTGAAGAACCTGTCACTGATGTGTCACG

TTTCCGTTGGCACACCTAGCGAACCTCTCTACGGATACTTCATCAACCGTGGCATGGAAG

TGCTCGAAGAGTACGAGCCCCAGCGGTTCCCCAACGCCACCAAGGTGTTCATCAACGGTG

CCTGGGTCGGTGTGCACACAAGCCCGAAAGATCTCGTGGATAGCATCATGCATCTGCGGC

GCTATGGTGACCTGAACCATGAAGCTTCCGTCATCCGCGACATTCGGGATCGAGAGTTCA

GGGTCGTCACGGATGCTGGTCGTGTTATGCGCCCGGTATTCACCGTGCAGCAAGAAGACA

----AGCTAGACGGGCCCGAGAAGGGCTCGTTGTGCATGACCAAGGAGCATCTTGCCGGT

TTGGATGACTGGCATC-TGGTCAACGAGGAGAG-------GGAAGAGATGG---CCA--C

GGGCTGGGAGTACCTCGTGAAGAGTGGGTGTATTGAGTACTTGGACGCCGAAGAAGAAGA

GACGGCAATGATTTGCATGACACCAGAAGACTTGGAGTCTTACCGCAAGGAGAAGTAC--

----------------------------CTCGATCAGAAACCCCAG------GAGCACAA

CGTGGAAGCCGAGCCCAACAAGCGACTCAAGACGAAGACCAACCCGACGACACACATGTA

CACCCACTGCGAGATTCATCCCAGTATGATCCAAGGATGGCCAGACCCGTGAGCACGCTC

TGCTCGCCTACACCCTGGGTGTGCGTCAGCTCATTGTTGCCATCAACAAGATGGACACCA

CC-CAGTGGTCTGAGGCCCGTTACCAGGAAATCATCAAGGAGA--CTTCCAACTTCATCA

AGAAGGTCGGCTTCAACCCCAAGACTGTTGCCTTCGTCCCCATCTCTGGTTTCCACGGCG

ACAACATGCTGTCTG----CTTCCACCAACTGCCCCTGGTACAAGGGCTGGGAGAAGGAG

GGCAAGGGTGGCGCCAAGGTCACCGGCAAGACCCTGCTTGAGGCCATCGACTCCGTCGAG

CCCCCCAAGCGTCCCAACGACAAGCCCCTCCGTCTTCCTCTCCAGGATGTCTACAAGATC

GGTGGTATTGGCACAGTGCCCGTTGGCCGTATCGAGACCGGTATCCTCAAGCCCGGCATG

GTCGTCACCTTCGCCCCCGCCAACGTCACCACTGAAGTCAAGTCCGTCGAGATGCACCAC

GAGCAGCTGCCCGAGGGTCTTCCCGGTGACAACGTTGGTTTCAACGTGAAGAACGTCTCC

GTCAAGGATATCCGCCGTGGCAACGTCGCCTCTGACTCCAAGAACGACCCCGCCATGGGC

GCTGCCTCCTTCAACGCCCAGGTCATTGTCCTGAACCACCCTGGTCAGGTCGGTGCCGGT

TACGCTCCCGTCCTCGACTGCCACACCGCCCAC-ATTGCTTGCAAGTTCTCCGAGATCCT

CGAGAAGATCGACCGCCGTACCGGTAAGTCGGTTGAGAACAACCCCAAGTTCATCAAGTC

CGGCGACT

>Acremonium_brachypenium_AB540570_CBS86673 Acremonium brachypenium genes for ITS1, 5.8S rRNA, ITS2 and 28S rRNA, partial sequence, strain: CBS 866.73.......

ACT-GAGTGT---AA----AAACTCCCAAACCCTATGCGAAC--CTACC---AAA-GTTG

CTTCGGCGG-A-CCGCCCC-GGGCGCCCT-GC-----------GC---ACCCGGGC-CTA

-GGCGGCCGCCGGAGG---CTCCAAACTCT--GAATTT---AC----AGTGGA--TTTCT

GAGTGG--CATAA-GC--------AAA--TAAA-TCAAAACTTTCAGCAACGGATCTCTT

GGTTCTGGCATCGATGAAGAACGCAGCAAAATGCGATAAGTAATGTGAATTGCAGAATTC

AGTGAATCATCGAATCTTTGAACGCACATTGCGCCCGCCAGTATTCTGGCGGGCATGCCT

GTCTGAGCGTCATTTCAACCCTCGGGACCCCG----TTC-----GCGGG-----------

--ACCCGGCGTTGGGGATCA-GCCCG-AAGCCC-T-CG-C-GGC--AGGCGG-CTGGCCC

C-TAAACCTAGTGGCGGTCCTCCCGGCGACCTCCTCTGCGCAGTAGTTTAAT--CGCCTC

GCAGCTGGAA--CGCGGGAAGGCCACGCCGTAAAACACCCA--ACTATTCA-CAA-AGTA

ACGGCGA-GTGAAGCGGCAACAGCTCAAATT-TGAAATCT-GGCC-TC-AC-GGTCCG-A

GTTGTAATTTGTAGAGGATGCTTCTGGCGACGTGCCTTCCGAGTTCCCTGG-AACGGGAC

GCCATAGAGGGTGAGAGCCCCGTCCGGTCGTGCACC-T-AGCCTCTGTGAAGCTCCTTCG

ACGAGTCGAGTAGTTTGGGAATGCTGCTCTAAATGGGAGGTATACGTCTTCTAAAGCTAA

ATACCGGCCAGAGACCGATAGCGCACAAGTAGAGTGATCGAAAGATGAAAAGCACTTTGA

AAAGAGGGTTAAATAGTACGTGAAATTGCTGAAAGGGAAGCGCTTATGACCAGACTTGGG

CTCGGTGAATCATCCGGCGTTCTCGCCGGTGCACTTT-G-CCGTCCCAGGCCAGCATCAG

TTCGCGCCGGGGGATAAAGGCTTCGGGAATGTAGCTCC-TTC---GGGAGTGTTATAGCC

CGTTGTGTAATACCCTGGCGTGGACTGAGGTCCGCGC-TCTGCAAGGATGCTGGCGTAAT

G-TCATCAGTGACCCGTC--CGACAGCTACACAATACCCACTGGGGTCTCGTGTGTCCCG

CGGAAACGCCCGAAGGTCAGGCTTGCGGTCTTGTCAAGAATTTGTCATTGATGTGTCACG

TTTCCGTTGGCACACCCAGCGAGCCCCTTTACGGATACTTCATCAACCGAGGCATGGAAG

TCCTCGAAGAATACGAGCCGCAGCGGTTCCCTAACGCAACGAAGGTGTTCATCAATGGCG

CCTGGGTTGGTGTGCATACCAGCCCGAAGGACCTTGTGGATAGTATCATGCACCTTCGTC

GTTATGGCGACCTTAACCACGAGGCATCGGTCATTCGTGACATTCGTGATCGAGAGTTCA

GGGTGGTCACGGATGCTGGACGTGTTATGCGTCCTGTGTTCACTGTCCAGCAAGAAGACA

----AGGTGGGGGAGCCCGAGAAGGGATCTCTGCGAATCACCAAGGAGCACATGGCTGGT

CTGAATGAATGGCATG-TGGTCAATGAAGAGAG-------GGAGGAGATGG---CGA--C

GGGATGGGAGTATTTGGTCAAGAGCGGCTGCATCGAATACTTGGATGCCGAAGAAGAGGA

GACAGCCATGATCTGCATGACACCAGAGGATCTGGAGTCTTACCGCCTTCAGAAG-----

----------------------------ATGGGCCACGATGTCACG------GAG-----

-GAGCAAGC---GCCTAACCAGCGCTTGAGAACGAAGACGAACCCAACCACACACATGTA

CACGCACTGCGAGATTCATCCCAGTATGATCCAAGGATGGCCAGACCCGTGAGCACGCTC

TGCTTGCCTACACCCTCGGTGTTCGTCAGCTCATCGTCGCCATCAACAAGATGGACACCA

CC-CAGTGGTCCGAGGCCCGTTACCAGGAGATCATCAAGGAGA--CCTCCAACTTCATCA

AGAAGGTCGGCTTCAACCCCAAGACTGTTGCCTTCGTCCCCATCTCCGGTTTCAACGGCG

ACAACATGCTTGAGG----CCTCCACCAACTGCCCCTGGTACAAGGGCTGGGAGAAGGAG

GGCGCCAAGGGTGCCAAGGCTACCGGCAAGACCCTGCTTGAGGCCATCGACTCCATCGAG

CCCCCCAAGCGTCCCAACGACAAGCCTCTCCGTCTTCCCCTCCAGGATGTCTACAAGATC

GGTGGTATTGGCACAGTGCCCGTCGGCCGTATCGAGACTGGTGTCCTCAAGCCCGGTATG

GTCGTTACCTTCGCTCCCGCCAACGTCACCACTGAGGTCAAGTCCGTCGAGATGCACCAC

GAGCAGCTTCCCGAGGGTGTCCCCGGTGACAACGTTGGTTTCAACGTGAAGAACGTCTCC

GTCAAGGACATTCGCCGTGGTAACGTCGCTTCTGACTCCAAGAACGACCCCGCCTCCGGC

GCTGCCTCCTTCAACGCCCAGGTCATCGTCCTGAACCACCCCGGTCAGGTTGGTGCTGGT

TACGCCCCCGTTCTCGACTGCCACACTGCCCAC-ATTGCTTGCAAGTTCTCCGAGATCCT

TGAGAAGATCGACCGCCGTACTGGTAAGTCGGTTGAGAACAACCCCAAGTTCATCAAGTC

CGGT----

>Acremonium_persicinum_FN706554_CBS31059 Acremonium persicinum 18S rRNA gene (partial), ITS1, 5.8S rRNA gene, ITS2 and 28S rRNA gene (partial), culture collection CBS:310.59......

CCT-GAGTCT--AAC----AAACTCCCAAACCC-CTGTGAAC--ATACC--TACT-GTTG

CTTCGGCGGGA-CCGCCCC-GGGCGCCTTCGCG----------GT--GCCCCGGAA-CCA

-GGCGCCCGCCGGGG-AC-ATC-AAACTCTT-G-ATTGT----TATAGTGGCAT-TCTCT

GAGTAAAACATA---C--------AAA--TAAG-TCAAAACTTTCAACAACGGATCTCTT

GGCTCTGGCATCGATGAAGAACGCAGCGAAATGCGATAAGTAATGCGAATTGCAGAATTC

AGTGAATCATCGAATCTTTGAACGCACATTGCGCCCGCTAGTATTCTGGCGGGCATGCCT

GTCTGAGCGTCATTTCAACCCTCGC-CCCCGGC---TTTT----GCTGG-----------

-GAG-CGGTGTTGGGGAT-CGGCC-----GCCCGT-CA-CT-----GGGAGG-CCGGCCC

C-GAAATAGAGTGGCGACCACGCCGTGTGC-TCCTCTGCGTAGTAGT-AAATCACCTC--

GCAGGCGGAC--AGCGGTGCGGCC-TGCCGTAAAACCCCCA--ACTCTTT--CTATAGTA

ACGGCGA-GTGAAGCGGCAACAGCTCAAATT-TGAAATCTTGGCCCT--A--GGCCCG-A

GTTGTAATTTGCAGAGGATGTTTCTGGCGAGGTGCCTTCCGAGTTCCCTGG-AACGGGAC

GCCATAGAGGGTGAGAGCCCCGTACGGTTGGTCGC--TAAGCCTCTGTGAAACTCCTTCG

ACGAGTCGAGTAGTTTGGGAATGCTGCTCTAAATGGGAGGTATACGTCTTCTAAAGCTAA

ATACCGGCCAGAGACCGATAGCGCACAAGTAGAGTGATCGAAAGATGAAAAGCACTTTGA

AAAGAGGGTTAAATAGTACGTGAAATTGTTGAAAGGGAAGCGCTTATGACCAGACTTGGA

C-CGGTTGATCATCCATCGTTCTCGGTGGTGCACTCT-G-CCGGTTCAGGCCAGCATCAG

TTTGTCCCGGGGGATAAAGGCTTTGGGAATGTGGCTCT-TC-----GGAGTGTTATAGCC

CATTGCGTAATACCCTGGGATAGACTGAGGTTCGCGC-TCCGCAAGGATGCTGGCGTAAT

GGTCATCAGTGACCCGTCTTCGACAGTTACACAACACCCACTGGGGTCTGGTGTGCCCAG

CAGAGACTCCAGAGGGCCAAGCTTGCGGTCTGGTCAAGAATTTGTCACTCATGTGTTACG

TGAGTGTCGGATCACCGTCTGAGCCTCTAATTGATTTCATGATCAACCGTGGCATGGAAG

TTGTGGAAGAGTATGAACCTCTAAGATACCCGCACGCGACTAAGATCTTCATCAATGGCA

GCTGGGTCGGCGTACATCAGGACCCCAAGCACCTTGTCAGCCATGTTCTAGATCTTCGCC

GAAAGTCTTACTTGCCATACGAAGTATCGCTCATTCGCGATATTCGAGACAGAGAGTTCA

AGATCTTTTCTGACGCTGGGCGTGTCATGCGACCGGTCTTCACCGTACGGCAAGAAGATG

GTC-AAGTCGGCGATCCTCCCAAGGGTTCTCTTGTACTGTCAAAGGAGCTGGTGAATCAG

CTAGCTAAGGAACAGGCCG----AGCCC-----------GAGA-CAGGAGAATCCAAGTC

TGGCTGGGACCGTTTGATTGGAGCGGGTGCCGTCGAGTACTTGGATGCTGAAGAAGAGGA

GACGTCCATGATATGCATGACGCCTGAGGATTTGGAATCTTATCGCATCCAAAAAAT---

------------------------------GGGATACGCAGCGCCT------GACGAGGG

TGAAGAACTGGAGCCCAATAAGCGCCTGAAAACGAAAACAAACCCAACGACACACATGTA

TACCCATTGCGAGATTCATCCCAGCATGATCCAAGGATGGCCAGACTCGTGAGCACGCTC

TGCTCGCTTACACCCTTGGTGTCAAGCAGCTGATCGTCGCCATCAACAAGATGGACACCA

CC-CAGTGGTCCGAGGCTCGTTTCAACGAGATTATCAAGGAGA--CCTCCAACTTCATCA

AGAAGGTCGGCTACAACCCCAAGACCGTTGCCTTCGTCCCTATCTCCGGTTTCCACGGCG

ATAACATGTTGGCTG----CCTCCACCAACTGCCCCTGGTACAAGGGCTGGGAGAAGGAG

ACCAAGGCTGG---CAAGTCCACTGGCAAGACCCTCCTCGAGGCTATTGACTCCATCGAG

CCCCCCAAGCGTCCTCTCGACAAGCCCCTCCGTCTTCCCCTCCAGGATGTTTACAAGATT

GGTGGTATTGGAACGGTTCCTGTCGGCCGTATCGAGACTGGTATCATCAAGCCCGGTATG

GTCGTTACCTTCGCTCCTTCCAACGTCACCACTGAAGTCAAGTCCGTCGAGATGCACCAC

GAGCAGCTTACCGAGGGTGTCCCCGGTGACAACGTTGGCTTCAACGTGAAGAACGTCTCC

GTCAAGGACATTCGCCGTGGCAACGTCGCCGGTGACTCCAAGAACGATCCCCCTCAGGGC

GCTGCTTCCTTCAACGCTCAGGTCATTGTTCTTAACCACCCTGGTCAGGTCGGTGCCGGT

TACGCTCCCGTCCTTGACTGCCACACCGCCCAC-ATTGCCTGCAAGTTCGCCGAGCTCCT

CGAGAAGATCGACCGCCGTACCGGTAAGGCTGTTGAGGCCTCCCCCAAGTTCATCAAGTC

TGGTGACT

>Acremonium_polychromum_AB540567_CBS18127 Acremonium polychromum genes for ITS1, 5.8S rRNA, ITS2 and 28S rRNA, partial sequence, strain: CBS 181.27.....

ACC-GAGTT----GC---AAAACTCCCAAACCCACTGTGAACCTATACC---ACT-GTTG

CTTCGGCGG-ATACGCCCC-GGGCGCACCCCCTCCAGGGGGTTGT--GCCCCGGAA-CCA

-GGCGCCCGCCGGGGG---ACCGAAACCTCT-GTATTT---ACCGTTTGAGTA---CTCT

GAGTGT--GATTT---------ACAAAATCAAAATTAAAACTTTCAACAACGGATCTCTT

GGCTCTAGCATCGATGAAGAACGCAGCGAAATGCGATAAGTAATGCGAATTGCAGAATTC

AGTGAATCATCGAATCTTTGAACGCACATTGCGCCCGCCAGTATTCTGGCGGGCATGCCT

GTCTGAGCGTCGTTTCGACCCTCGCGCCC-GGC---TTCT----GTCGGGGG--------

-----CGGTGTTGGGGAT-CGGCC---ACACCC----A-CTCCGGTGGGAGG-CCGGCCC

C-TAAATCCAGTGGCGACCACGCTGTAGCC-TCCCCTGCGTAGTACTAAAACCACCTC--

GCAGGCGGAG--AGCGGTGCGGCC-CGCCGTAAAACCCCCA--ACT-TTTA-CAA-AGTA

ACGGCGA-GTGAAGCGGCAACAGCTCAAATT-TGAAATCT-GGCCC-C-A--GGCCCG-A

GTTGTAATTTGCAGAGGATGTTTCTGGCGAGGTGCCTTCCGAGTTCCCTGG-AACGGGAC

GCCATAGAGGGTGAGAGCCCCGTACGGTTGGTCGC--TAAGCCTCTGTGAAACTCCTTCG

ACGAGTCGAGTAGTTTGGGAATGCTGCTCTAAATGGGAGGTGTACGCCTTCTCAAGCTAA

ATACCGGCTAGAGACCGATAGCGCACAAGTAGAGTGATCGAAAGATGAAAAGCACTTTGA

AAAGAGGGTTAAATAGTACGTGAAATTGTTGAAAGGGAAGCGCTCTTGACCAGACTTGCG

C-CGGTTGATCATCCACCGTTCTCGGTGGTGCACTCT-G-CCGGCTCAGGCCAGCATCAG

TTCGGCCCGGGGGATAAAGGCTTCGGGAATGTGGCTCTCTCC--GGGGAGTGTTATAGCC

CGTTGCGTAATACC-TGGACCGGACTGAGGTTCGCGCATCTGCATGGATGCTGGCGTAAT

GGTCATCAGTGACCCGTCTTAGACAGCTGCACAACACCCACTGGGGACTGGTCTGCCCAG

CTGAAACCCCAGAAGGCCAGGCCTGTGGTCTGGTCAAGAACTTATCACTCATGTGCTATG

TCAGTGTCGGATCGCCATCTGATCCTCTTATTGACTTCATGATCAACCGTGGTATGGAAG

TTGTGGAGGAATACGAGCCACTCAGATACCCGCACGCAACAAAGATCTTCATCAACGGAA

GCTGGGTTGGTGTGCACCAAGACCCCAAGCATCTCGTCAGTCATGTTCTCGATCTTCGCC

GAAAGTCGTACCTGCCATACGAAGTCTCTCTTGTGCGTGATATCCGCGACCGAGAGTTCA

AGATCTTCTCAGATGCTGGCCGTGTGATGCGTCCCGTCTTCACCGTTCGACAGGAAGATG

GTC-AGGTCGGCGAACCTGCGAAGGGCTCACTCGTGCTATCAAAGGAGCTCGTCAACCAG

CTTGCCAAGGAACAGGCAG----AG-CCG----------GAGA-CAGGCGAATCGAAATC

TGGCTGGGACCGCCTTATTGGTGCTGGTGCAGTCGAGTATCTCGATGCCGAGGAGGAGGA

GACAGCCATGATTTGCATGACACCAGAAGACCTGGAGTCTTACCGTATCCAGAAGCT---

------------------------------GGGCTACGCCGCACCT------GACGAGGG

TGAGGAGCTGGAGCCTAACAAGCGATTGAGGACAAAGACAAACCCGACAACCCATATGTA

CACCCACTGCGAGATTCATCCCAGTATGATCCAAGGATGGCCAGACTCGTGAGCACGCTC

TGCTCGCCTACACCCTCGGTGTCAAGCAGCTCATCGTTGCCATCAACAAGATGGACACTG

CC-CAGTGGGCTGAGGCTCGTTTCAACGAGATCATCAAGGAGA--CCTCCAACTTCATCA

AGAAGGTCGGCTACAACCCCAAGACTGTTGCCTTCGTCCCCATCTCTGGTTTCCACGGCG

ACAACATGATTGCGC----CCACCACCAACGCTCCCTGGTACAAGGGTTGGGAGAAGGAG

ACCAAGGCTGG---CAAGTCCACCGGCAAGACCCTCCTCGAGGCCATCGACTCCATCGAG

CCCCCCAAGCGCCCCGTCGACAAGCCCCTCCGTCTTCCCCTCCAGGATGTCTACAAGATC

GGTGGTATTGGCACAGTCCCTGTCGGCCGTATCGAGACCGGTGTCATCAAGCCCGGTATG

GTCGTTACCTTCGCCCCCTCCAACGTCACCACTGAAGTCAAGTCCGTCGAGATGCACCAC

GAGCAGCTCACTGAGGGTGTCCCCGGTGACAACGTTGGCTTCAACGTGAAGAACGTCTCC

GTCAAGGACATTCGTCGTGGCAACGTCGCCTCCGACTCCAAGAACGACCCGGCCCAGGCT

GCTGCCTCCTTCAACGCCCAGGTCATCGTTCTCAACCACCCTGGTCAGGTCGGTGCCGGC

TACGCCCCCGTCCTCGACTGCCACACCGCCCAC-ATTGCGTGCAAGTTCTCCGAGCTCCT

CGAGAAGATTGACCGCCGTACCGGTAAGGCTGTTGAGGAGGCCCCCAAGTTCATCAAGTC

TGGTGACT

>Acremonium_strictum_GQ376096_CBS34670, 627 bases, 0 checksum......

ACCAGAGTGCCCTA---GGC-TCT-CC-AACCCATTGTGAAC--TTACC--AAAC-GTTC

CCTCGGCGGG----CTCA----GCGC----GCG----------GTGGCCTCCGGGCCTCC

GGGCGTCCGCCGGGG-AAAA-CCAAACCCT--G-ATTT------AAT-CAGTATTTCTCT

GAGGGG--CGAAA-GCCCGAAAACAAAA-TGAA-TCAAAACTTTCAACAACGGATCTCTT

GGCTCTGGCATCGATGAAGAACGCAGCGAAATGCGATAAGTAATGTGAATTGCAGAATTC

AGTGAATCATCGAATCTTTGAACGCACATTGCGCCCGCCGGCACTCCGGCGGGCATGCCT

GTCCGAGCGTCATTTCAACCCTCAGGCCCACCC---TTCC----GGGGGAG-------CG

GGCC-TGGTTCTGGGGAT-CGGC-----GGCC-------CTCG---CGGCCC-CCGTCCC

T-CAAATTCAGTGGCGGTCGCGCCGCAGCC-TCCCCTGCGTAGTAGC--ACA-ACCTC--

GCACC-GGAG--AGCGGAACGACCACGCCGTAAAACACCCA--ATTTTTT---AA-AGTA

ACGGCGA-GTGAAGCGGCAACAGCTCAAATT-TGAAATCT-GGCC-TC-AC-GGTCCG-A

GTTGTAATTTGTAGAGGATGCTTTTGGCGAGGTGCTTTCCGAGTTCCCTGG-AACGGGAC

GCCATAGAGGGTGAGAGCCCCGTACGGTAAGACCACCG-AGCCTCTGTAAAGCTCCTTCG

ACGAGTCGAGTAGTTTGGGAATGCTGCTCTAAATGGGAGGTGTACGTCTTCTAAAGCTAA

ATACCGGCCAGAGACCGATAGCGCACAAGTAGAGTGATCGAAAGATGAAAAGCACTTTGA

AAAGAGGGTTAAAAAGTACGTGAAATTGTTGAAAGGGAAGCATTCATGACCAGACTTGGG

CTTGGTTGAACATCCGGCGTTCTCGCCGGTGCACTCT-G-CCAGTCCAGGCCAGCATCAG

TTTGCCCCGGGGGACAAAGGCGGTGGGAATGTGGCTCT-CT-TCGGGGAGTGTTATAGCC

CGCCGTGTAATGCCCTGGGGCGGACTGAGGAACGCGCTTCGGCACGGATGC---------

--------------------CGTCAATTACACAACACTCATTGGGGTCTTGTCTGCCCTG

CAGAAACACCCGAAGGACAGGCTTGTGGTCTTGTGAAGAACCTGTCGCTCATGTGCTACG

TGAGTCTAGGTTCGCCTGCAGACCCAATCGAGAGGTTCATGATCGATCGTGGCATGGATC

TGGTGGAAGAGTACGAACCGCTGCGTTTCCCGCATGCTACCAAGGTTTTCGTCAACGGCG

TGTGGTGTGGTGTTCACCAAGAGCCCAAGCAGTTGGTCGATGCCCTGGTCACAACCCGCC

GTCGCGGACTGATCTCCAACGAGGTATCGCTTGTTCGCGATATCAGAGATCGTGAATTCA

AGGTCTTCTCCGACGCTGGTCGTGTGATGCGTCCTGTGTTCACTGTTGAGCAGAGTGATG

ATTCCGATCAAGGAAT-TGAGAAGGGCAGCCTGATCCTCACCAAGGATATGGTCAACAAG

CTTGACAAGGAGCGGGCTGATGAGTTC-------------GAGGACGGCGAACGCTTGTC

A---TGGCAGGAACTTACCGCTCTCGGAGCAGTCGAGTATCTCGATGCCGAGGAAGAGGA

GACGGCGATGATCTGCATGACACCTGAGGATCTCGAGCTTTACAGGCTGCAGAAAGC---

-----------------------------TGGTATTCAGATTCCC-------GAAGAGGA

CCCGGGTGATGATCCCAACAAGCGACTGAAGACGAAGACCAACCCAACCACTCACACATA

CACACACTGCGAGATTCATCCCAGTATGATCCAAGGATGGCCAGACTCGTGAGCACGCCC

TGCTCGCCTACACCCTTGGTGTGCGTCAGCTCATCGTCGCCATCAACAAGATGGACACCA

CC-AAGTGGTCCGAGGCCCGTTACCAGGAGATTATCAAGGAGA--CCTCCAACTTCATCA

AGAAGGTCGGCTACAACCCCAAGACCGTCGCCTTCGTCCCCATCTCCGGTTTCCACGGTG

ACAACATGCTTGCCC----CCACCACCAACGCCCCCTGGTACAAGGGTTGGGAGCGTGAG

ATCAAGGGCAA---CAAGCAGACCGGCAAGACCCTCCTCGAGGCCATTGACGGTTGCGAG

CCTCCCAAGCGCCCCAACGACAAGCCCCTCCGTCTTCCCCTCCAGGATGTCTACAAGATT

GGTGGTATTGGAACGGTTCCTGTCGGCCGTATCGAGACTGGTATCCTCAAGCCCGGTATG

GTCGTCACCTTCGCTCCCGCCAACGTCACCACTGAAGTCAAGTCCGTCGAGATGCACCAC

GAGCAGCTCACTGAGGGTCTTCCCGGTGACAACGTCGGCTTCAACGTGAAGAACGTCTCC

GTCAAGGAGATCCGTCGTGGTAACGTTGCTGGTGACTCCAAGAACGACCCCCCGCTTGGT

GCCGCCTCTTTCGAGGCCCAGGTCATCGTCCTCAACCACCCCGGTCAGGTCGGCCCCGGT

TACGCTCCCGTCCTCGATTGCCACACTGCCCAC-ATTGCTTGCAAGTTCGCCGAGATCAA

GGAGAAGATCGACCGCCGTACCGGAAAGTCCGTTGAGGATGCTCCCAAGTTCATCAAGTC

TGGTGACT

>Trichothecium_hongkongense_CBS117586 Trichothecium hongkongense culture CBS:117586 small subunit ribosomal RNA gene, partial sequence; internal transcribed spacer 1 and 5.8S ribosomal RNA gene, complete sequence; and internal transcribed spacer 2, partial sequence.....

ACC-GAGTTT---AC--ACAAACTCCC-AACCCTTTGTGAACC-TTACC--TACC-GTTG

CTTCGGCGG-A-CCGCCCC-GGGCGCT---GC-----------GT--GCCCCGGAC-CCA

-GGCGCCCGCCGGGG-ACCATTCAAACCCT--GTTTTTTA-ACCA---GTGTAT-CTTCT

GAGCGAGCCGAAAGGC-----AACAAAA-CAA--TCAAAACTTTCAACAACGGATCTCTT

GGTTCTGGCATCGATGAAGAACGCAGCGAAATGCGATAAGTAATGTGAATTGCAGAATTC

AGTGAATCATCGAATCTTTGAACGCACATTGCGCCCGCCAGTATTCTGGCGGGCATGCCT

GTCCGAGCGTCATTTCAACCCTCGGGCCCCCCC----TCTAAC--CGGGGG-------CG

GGAC-CGGTGTTGGGGCTCAGGC------GTCCTCCTCTT------GGGCGC-CTGTCCC

C-TAAATGCAGTGGCGGCCTCGCCGCTGCC-TCCTCCGCGTAGTAGC--ACAAACCTC--

GCGTGTGGAA--GGCGGCGCGGCCACGCCGTAAAACCCCCA--ACT-TTTACCAA-----

--GGCGA-GTGAAGCGGCAACAGCTCAAATT-TGAAAGCT-GGC--TCT-CGGGCCCGCA

-TTGTAATTTGCAGAGGATGCTTCTGGCGACGCGCCTTCCGAGTTCCCTGG-AACGGGAC

GCCATAGAGGGTGAGAGCCCCGTCCGGTCGTGCGCC-T-AGCCTCTGTGAAGCTCCTTCG

ACGAGTCGAGTAGTTTGGGAATGCTGCTCAAAATGGGAGGTATACGTCTTCTAAAGCTAA

ATACCGGCCAGAGACCGATAGCGCACAAGTAGAGTGATCGAAAGATGAAAAGCACTTTGA

AAAGAGAGTTAAAAAGTACGTGAAATTGTTGAAAGGGAAGCGCTCATGACCAGACTTGGG

CTGGCTCGATCATCCGGCGTTCTCGCCGGTGCACTC-GGGCCGCT-CAGGCCAGCATCAG

CTTTCGCCGGGGGATAAAGGCGGCGGGAATGTGGCTCC-CTCG---GGAGTGTTATAGCC

CGCCGTGTAATACCCTGGTGAGGGCTGAGGTTCGCGCGTATGCACGGATGCTGGCGTAAT

GGTCATCAGCGACCCGTCTTCGGCAATTGCACAATACGCATTGGGGCCTGGTGTGCCCGG

CCGAGACCCCTGAAGGTCAAGCCTGTGGTCTTGTCAAGAATCTATCTCTCATGTGTTACG

TTAGTGTTGGCTCGCCGGCAGACCCTCTGGTGGATTTCATGATCAACAGAGGCATGGAAG

TCATCGAGGAATACGAACCATTGAGGTTTCCGCATGCCACAAAGATCTTTATCAATGGTA

GCTGGGTTGGTGTTCACCAGGATCCTAAGCATCTTGTCAGCCAGGTTCTCGACTTGCGCC

GCAAGTCATATCTCAACTACGAGGTTTCACTTGTTCGAGACATCCGGGATCGAGAGTTCA

AGATCTTCTCTGATGCTGGCCGCGTGATGAGGCCAGTATTCACAGTACGGCAGGAAGATG

GGC-AGATTGGGGATCCTCCCAAGGGAACTCTTRTGTTGACCAAGGAACACGTGAACCAG

CTGGCAAGGGAACAGGCCGGTGAGCCACCCGAGGCCAAT-GATGATGGTGATATCAAGCC

GGGCTGGGAAGGTCTGGTTAGTGCGGGCGCTGTTGAGTATCTTGATGCTGAGGAGGAGGA

GACGGCTATGATATGCATGACACCCGACGACCTCGAAATTTACCGCGCGCAAAAAAC---

---------------------------GGAAGGCTACGAACCG---------CCTGCTTT

CGACCCAGCGGAACCCAACAAACGAGTAAGAACCAGGATCAATGCGTCAACACACATCTA

TACACACTGCGAAATCCA-CCCA------TCCAAGGATGGCCAGACTCGTGAGCACGCTC

TGCTCGCCTACACCCTGGGTGTCAAGCAGCTCATCGTTGCCATCAACAAGATGGACACCA

CC-CAGTGGTCTGAGGCCCGTTACCAGGAGATCATCAAGGAGA--CCTCCAACTTCATCA

AGAAGGTCGGCTACAACCCCAAGACTGTTGCCTTCGTCCCCATCTCCGGTTTCCACGGTG

ACAACATGCTTGCTG----CCTCCTCCAACTGCCCCTGGTACAAGGGTTGGGAGAAGGAG

ACCAAGGCTGG---CAAGTCCACCGGCAAGACCCTCCTCGAGGCTATCGACTCCATCGAG

CCCCCCAAGCGTCCTCTTGAGAAGCCCCTGCGTCTCCCTCTCCAGGATGTCTACAAGATC

GGTGGTATTGGAACAGTCCCTGTCGGCCGTATCGAGACTGGTACCCTCAAGCCCGGTATG

GTCGTCACCTTCGCTCCTTCCAACGTCACCACTGAAGTCAAGTCCGTTGAGATGCACCAC

GAGCAGCTCACTGAGGGCTTCCCCGGTGACAACGTTGGTTTCAACGTGAAGAACGTTTCC

GTCAAGGATATCCGCCGTGGCAACGTCGCCTCTGACTCCAAGAACGACCCTGCCCTCGGT

GCTGCCTCCTTCGACGCCCAGGTCATCGTCCTCAACCACCCTGGTCAGGTCGGTGCTGGT

TACGCTCCCGTCCTGGACTGCCACACTGCCCAC-ATTGCCTGCAAGTTCGCCGAGATCAA

GGAGAAGATCGACCGTCGTACTGGTAAGGCCGTTGAGACTGCCCCCAAGTTCATCAAGTC

TGGTGACT

>Trichothecium_hongkongense_CBS101444T Trichothecium hongkongense culture CBS:101444 small subunit ribosomal RNA gene, partial sequence; internal transcribed spacer 1 and 5.8S ribosomal RNA gene, complete sequence; and internal transcribed spacer 2, partial sequence.....

ACC-GAGTTT---AC--ACAAACTCCC-AACCCTTTGTGAACC-TTACC--TACC-GTTG

CTTCGGCGG-A-CCGCCCC-GGGTGCT---GC-----------GT--GCCCCGGAC-CCA

-GGCGCCCGCCGGGG-ACCATTCAAACCCT--GTTTTTTA-ATCA---GTGTAT-CTTCT

GAGCGAGCCGAAAGGC-----AACAAAA-CAA--TCAAAACTTTCAACAACGGATCTCTT

GGTTCTGGCATCGATGAAGAACGCAGCGAAATGCGATAAGTAATGTGAATTGCAGAATTC

AGTGAATCATCGAATCTTTGAACGCACATTGCGCCCGCCAGTATTCTGGCGGGCATGCCT

GTCCGAGCGTCATTTCAACCCTCGGGCCCCCCC----TCTAAC--CGGGGG-------CG

GGAC-CGGTGTTGGGGCTCAGGC------GTCCTTCTC--------GGGCGC-CTGTCCC

C-TAAATGCAGTGGCGGCCTCGCCGCTGCC-TCCTCCGCGTAGTAGC--ACAAACCTC--

GCGGCTGGAA--GGCGGCGCGGCCACGCCGTGAAACCCCCA--ACT-TTTACCAA-----

--GGCGA-GTGAAGCGGCAACAGCTCAAATT-TGAAAGCT-GGC--TCT-CGGGCCCGCA

-TTGTAATTTGCAGAGGATGCTTCTGGCGACGCGCCTTCCGAGTTCCCTGG-AACGGGAC

GCCATAGAGGGTGAGAGCCCCGTCCGGTCGTGCGCC-T-AGCCTCTGTGAAGCTCCTTCG

ACGAGTCGAGTAGTTTGGGAATGCTGCTCAAAATGGGAGGTATACGTCTTCTAAAGCTAA

ATACCGGCCAGAGACCGATAGCGCACAAGTAGAGTGATCGAAAGATGAAAAGCACTTTGA

AAAGAGAGTTAAAAAGTACGTGAAATTGTTGAAAGGGAAGCGCTCATGACCAGACTTGGG

CTGGCTCGATCATCCGGCGTTCTCGCCGGTGCACTC-GGGCCGCT-CAGGCCAGCATCAG

CTTTCGCCGGGGGATAAAGGCGGCGGGAATGTGGCTCC-CTCG---GGAGTGTTATAGCC

CGCCGTGCAATACCCTGGTGAGGGCTGAGGTTCGCGCGTATGCACGGATGCTGGCGTAAT

GGTCATCAGCGACCCGTCTTCGACAATTGCACAATACGCATTGGGGCTTAGTGTGCCCGG

CCGAGACTCCTGAGGGTCAGGCCTGTGGTCTTGTCAAGAATCTATCTCTCATGTGTTACG

TTAGTGTTGGCTCACCGGCAGACCCTCTGGTGGATTTCATGATCAACAGAGGCATGGAAG

TCATCGAGGAATATGAACCATTGAGATTTCCACATGCCACAAAGATCTTTATCAATGGTA

GCTGGGTTGGTGTTCACCAGGATCCCAAGCATCTTGTCAGCCAGGTTCTCGACTTGCGTC

GCAAGTCTTATCTCAATTATGAGGTTTCCCTTGTTCGAGATATCAGGGATCGAGAATTCA

AGATCTTCTCTGATGCCGGCCGCGTGATGCGGCCAGTATTTACAGTCCGGCAGGAAGATG

GGC-AGATTGGGGACCCTCCCAAGGGAACCCTTGTATTGACCAAGGAACACGTGAACCAG

CTAGCGAGAGAACAGGCCGGTGAGCCACCTGAGGTCAAT-GAAGACGGTGATGTCAAGCC

GGGCTGGGAAGGCCTGGTCAGTGCAGGCGCTGTTGAGTATCTTGATGCTGAGGAGGAGGA

GACAGCTATGATATGCATGACACCCGATGACCTCGAAATATACCGCGCGCAGAAAAC---

---------------------------AGAAGGCTATGAGCCG---------CCGGCCTT

CGACCCAGCGGAACCCAACAAACGAGTCAGAACTAAGATCAATGCGTCAACACACATCTA

TACACACTGC-------------------TCCAAGGATGGCCAGACCCGTGAGCACGCCC

TGCTCGCCTACACTCTGGGTGTCAAGCAGCTCATCGTCGCCATCAACAAGATGGACACCA

CC-CAGTGGTCTGAGGCCCGTTACCAGGAAATCATCAAGGAGA--CTTCCAACTTCATCA

AGAAGGTCGGCTACAACCCCAAGAGTGTTGCCTTCGTCCCCATCTCTGGTTTCCACGGCG

ACAACATGCTTGCTG----CCTCCACCAACTGCCCCTGGTACAAGGGTTGGGAGAAGGAG

ACCAAGGCTGG---CAAGTCCACCGGCAAGACCCTCCTCGAGGCCATCGACTCCATCGAG

CCCCCCAAGCGTCCTCTTGACAAGCCCCTGCGTCTCCCTCTCCAGGATGTCTACAAGATC

GGTGGTATTGGAACAGTCCCTGTCGGCCGTATCGAGACTGGTACCCTCAAGCCCGGTATG

GTCGTCACCTTCGCTCCCTCCAACGTCACCACTGAAGTCAAGTCCGTTGAGATGCACCAC

GAGCAGCTCCCTGAGGGCTTCCCCGGTGACAACGTTGGTTTCAACGTGAAGAACGTTTCC

GTCAAGGATATCCGTCGTGGCAACGTCGCCTCTGACTCCAAGAACGACCCCGCCATGGGT

GCCGCCTCCTTCGACGCCCAGGTCATCGTCCTCAACCACCCCGGTCAGGTCGGTGCTGGT

TACGCCCCCGTCCTGGACTGCCACACTGCCCAC-ATTGCCTGCAAGTTCGCCGAGATCAA

GGAGAAGATCGACCGTCGTACTGGTAAGGCTGTTGAGTCTGCCCCCAAGTTCATCAAGTC

TGGTGACT

>Trichothecium_hongkongense_CBS102186 Trichothecium hongkongense culture CBS:102186 small subunit ribosomal RNA gene, partial sequence; internal transcribed spacer 1 and 5.8S ribosomal RNA gene, complete sequence; and internal transcribed spacer 2, partial sequence.....

ACC-GAGTTT---AC--ACAAACTCCC-AACCCTTTGTGAACC-TTACC--TACC-GTTG

CTTCGGCGG-A-CCGCCCC-GGGTGCT---GC-----------GT--GCCCCGGAC-CCA

-GGCGCCCGCCGGGG-ACCATTCAAACCCT--GTTTTTTA-ATCA---GTGTAT-CTTCT

GAGCGAGCCGAAAGGC-----AACAAAA-CAA--TCAAAACTTTCAACAACGGATCTCTT

GGTTCTGGCATCGATGAAGAACGCAGCGAAATGCGATAAGTAATGTGAATTGCAGAATTC

AGTGAATCATCGAATCTTTGAACGCACATTGCGCCCGCCAGTATTCTGGCGGGCATGCCT

GTCCGAGCGTCATTTCAACCCTCGGGCCCCCCC----TCTAAC--CGGGGG-------CG

GGAC-CGGTGTTGGGGCTCAGGC------GTCCTTCTC--------GGGCGC-CTGTCCC

C-TAAATGCAGTGGCGGCCTCGCCGCTGCC-TCCTCCGCGTAGTAGC--ACAAACCTC--

GCGGCTGGAA--GGCGGCGCGGCCACGCCGTGAAACCCCCA--ACT-TTTACCAA-----

--GGCGA-GTGAAGCGGCAACAGCTCAAATT-TGAAAGCT-GGC--TCT-CGGGCCCGCA

-TTGTAATTTGCAGAGGATGCTTCTGGCGACGCGCCTTCCGAGTTCCCTGG-AACGGGAC

GCCATAGAGGGTGAGAGCCCCGTCCGGTCGTGCGCC-T-AGCCTCTGTGAAGCTCCTTCG

ACGAGTCGAGTAGTTTGGGAATGCTGCTCAAAATGGGAGGTATACGTCTTCTAAAGCTAA

ATACCGGCCAGAGACCGATAGCGCACAAGTAGAGTGATCGAAAGATGAAAAGCACTTTGA

AAAGAGAGTTAAAAAGTACGTGAAATTGTTGAAAGGGAAGCGCTCATGACCAGACTTGGG

CTGGCTCGATCATCCGGCGTTCTCGCCGGTGCACTC-GGGCCGCT-CAGGCCAGCATCAG

CTTTCGCCGGGGGATAAAGGCGGCGGGAATGTGGCTCC-CTCG---GGAGTGTTATAGCC

CGCCGTGCAATACCCTGGTGAGGGCTGAGGTTCGCGCGTATGCACGGATGCTGGCGTAAT

GGTCATCAGCGACCCGTCTTCGACAATTGCACAATACGCATTGGGGCTTAGTGTGCCCGG

CCGAGACTCCTGAGGGTCAGGCCTGTGGTCTTGTCAAGAATCTATCTCTCATGTGTTACG

TTAGTGTTGGCTCACCGGCAGACCCTCTGGTGGATTTCATGATCAACAGAGGCATGGAAG

TCATCGAGGAATATGAACCATTGAGATTTCCACATGCCACAAAGATCTTTATCAATGGTA

GCTGGGTTGGTGTTCACCAGGATCCCAAGCATCTTGTCAGCCAGGTTCTCGACTTGCGTC

GCAAGTCTTATCTCAATTATGAGGTTTCCCTTGTTCGAGATATCAGGGATCGAGAATTCA

AGATCTTCTCTGATGCCGGCCGCGTGATGCGGCCAGTATTTACAGTCCGGCAGGAAGATG

GGC-AGATTGGGGACCCTCCCAAGGGAACCCTTGTATTGACCAAGGAACACGTGAACCAG

CTAGCGAGAGAACAGGCCGGTGAGCCACCTGAGGTCAAT-GAAGACGGTGATGTCAAGCC

GGGCTGGGAAGGCCTGGTCAGTGCAGGCGCTGTTGAGTATCTTGATGCTGAGGAGGAGGA

GACAGCTATGATATGCATGACACCCGATGACCTCGAAATATACCGCGCGCAGAAAAC---

---------------------------AGAAGGCTATGAGCCG---------CCGGCCTT

CGACCCAGCGGAACCCAACAAACGAGTCAGAACTAAGATCAATGCGTCAACACACATCTA

TACACACTGCGAAATCCACCCAAG-----TCCAAGGATGGCCAGACCCGTGAGCACGCCC

TGCTCGCCTACACTCTGGGTGTCAAGCAGCTCATCGTCGCCATCAACAAGATGGACACCA

CC-CAGTGGTCTGAGGCCCGTTACCAGGAAATCATCAAGGAGA--CTTCCAACTTCATCA

AGAAGGTCGGCTACAACCCCAAGAGTGTTGCCTTCGTCCCCATCTCTGGTTTCCACGGCG

ACAACATGCTTGCTG----CCTCCACCAACTGCCCCTGGTACAAGGGTTGGGAGAAGGAG

ACCAAGGCTGG---CAAGTCCACCGGCAAGACCCTCCTCGAGGCCATCGACTCCATCGAG

CCCCCCAAGCGTCCTCTTGACAAGCCCCTGCGTCTCCCTCTCCAGGATGTCTACAAGATC

GGTGGTATTGGAACAGTCCCTGTCGGCCGTATCGAGACTGGTACCCTCAAGCCTGGTATG

GTCGTCACCTTCGCTCCCTCCAACGTCACCACTGAAGTCAAGTCCGTTGAGATGCACCAC

GAGCAGCTCCCTGAGGGCTTCCCCGGTGACAACGTTGGTTTCAACGTGAAGAACGTTTCC

GTCAAGGATATCCGTCGTGGCAACGTCGCCTCTGACTCCAAGAACGACCCCGCCATGGGT

GCCGCCTCCTTCGACGCCCAGGTCATCGTCCTCAACCACCCCGGTCAGGTCGGTGCTGGT

TACGCCCCCGTCCTGGACTGCCACACTGCCCAC-ATTGCCTGCAAGTTCGCCGAGATCAA

GGAGAAGATCGACCGTCGTACTGGTAAGGCTGTTGAGTCTGCCCCCAAGTTCATCAAGTC

TGGTGACT

>Trichothecium_roseum_PGTR-1 small subunit ribosomal RNA gene, partial sequence; internal transcribed spacer 1, 5.8S ribosomal RNA gene, and internal transcribed spacer 2, complete sequence; and large subunit ribosomal RNA gene, partial sequence...

ATA-GAGTTA---ACAAAACAACTCCC-AACCCTTTGTGAACC-TTACC--TACC-GTTG

CTTCGGCGG-A-CCGCCCC-GGGCGCT---GC-----------GT--GCCCCGGAC-CCA

AGGCGCCCGCCGGGG-ACCACACGAACCCT--GTTTAACA-AACA-T-GTGTAT-CCTCT

GAGCGAGCCGAAAGGC-----AACAAAA-CAAA-TCAAAACTTTCAACAACGGATCTCTT

GGTTCTGGCATCGATGAAGAACGCAGCGAAATGCGATAAGTAATGTGAATTGCAGAATTC

AGTGAATCATCGAATCTTTGAACGCACATTGCGCCCGCCAGTATTCTGGCGGGCATGCCT

GTCCGAGCGTCATTTCAACCCTCGGGCCCCCCCCTTTTCCCCTCGCGGGGGAGGG-GGCG

GGCC-CGGCGTTGGGGCCCAGGC------GTCCTCCAA--------GGGCGC-CTGTCCC

C-GAAACCCAGTGGCGGCCTCGCCGCTGCC-TCCTCCGCGTAGTAGC--ACAAACCTC--

GCGGGCGGAA--GGCGGCGCGGCCACGCCGTAAAACCCCAA--ACT-TTTACCAAGAGTA

ACGGCGA-GTGAAGCGGCAACAGCTCAAATT-TGAAAGCT-GGC--TCT-CGGGCCCGCA

-TTGTAATTTGCAGAGGATGCTTCTGGCGACGCGCCTTCCGAGTTCCCTGG-AACGGGAC

GCCATAGAGGGTGAGAGCCCCGTCCGGTCGTGCGCC-T-AGCCTCTGTGAAGCTCCTTCG

ACGAGTCGAGTAGTTTGGGAATGCTGCTCAAAATGGGAGGTATACGTCTTCTAAAGCTAA

ATACCGGCCAGAGACCGATAGCGCACAAGTAGAGTGATCGAAAGATGAAAAGCACTTTGA

AAAGAGAGTTAAAAAGTACGTGAAATTGTTGAAAGGGAAGCGCTCATGACCAGACTTGGG

CTGGCCCGATCATCCGGCGTTCTCGCCGGTGCACTC-GGGCCGCC-CAGGCCAGCATCAG

CTTTCGCCGGGGGATAAAGGCGGCGGGAATGTGGCTCC-CTCG---GGAGTGTTATAGCC

CGCCGTGCAATACCCTGGTGGGGGCTGAGGTTCGCGCGTATGCACGGATGCTGGCGTAAT

GGTTATCAGCGACCCGTCTT----------------------------------------

------------------------------------------------------------

--------------CCAGCTGACCCTCTGGTGGATTTCATGATTAACCGAGGCATGGAGG

TCATCGAGGAATACGAACCGTTGAGGTTTCCGCATGCTACAAAGATTTTTATCAATGGTA

GCTGGGTTGGTGTCCACCAAGACCCCAAACATCTTGTTAGTCAGGTTCTTGACTTGCGTC

GCAAGTCTTACCTCAATTACGAGGTTTCCCTTGTTCGCGATATTAGGGATCGAGAATTCA

AGATCTTTTCGGATGCTGGCCGTGTTATGCGACCTGTATTCACAGTACGACAGGAAGACG

GGC-AAATCGGGGAACCCCCTAAAGGTACGCTTGTATTAACGAAAGAGCACGTGAACCAG

CTGGCGAGAGAGCAGGCTGGCGAGCCCCTTGAAGCCGGC-GAAGACGGCGACACCAAGCC

AGGCTGGGAAGGGTTGGTCAGCGCAGGCGCTGTCGAATATCTTGACGCAGAGGAGGAGGA

GACGGCAATGATATGTATGACACCTGACGACCTTGAGATCTATCGCGCGCAAAAGAC---

---------------------------AGATGGCTATGAACCA---------CCTGCCTT

TGACCCAGCGGAACCCAACAAGCGAGTGAGGACCAGGATTAATGCATCGACACATATCTA

TACTCACTGCGAGATTCATCCGAGCATGATCCAAGGATGGCCAGACCCGTGAGCACGCCC

TGCTCGCCTACACCCTCGGTGTTAAGCAGCTCATTGTTGCCATCAACAAGATGGACACCA

CC-CAGTGGTCCGAGGCTCGTTACCAGGAGATCATCAAGGAGA--CCTCCAACTTCATCA

AGAAGGTCGGCTACAACCCCAAGACTGTCGCCTTCGTCCCCATCTCCGGTTTCCACGGTG

ACAACATGCTTGCTG----CCTCCACCAACTGCCCCTGGTACAAGGGTTGGGAGAAGGAG

ACCAAGGCTGG---CAAGTCTACCGGCAAGACCCTCCTCGAGGCCATTGACGCCATCGAG

CCCCCCAAGCGTCCTCTTGACAAGCCCCTGCGTCTTCCCCTCCAGGATGTCTACAAGATT

GGCGGTATCGGCACAGTCCCCGTCGGCCGTATCGAGACCGGTACTCTTAAGCCCGGTATG

GTCGTTACCTTCGCTCCTGCCAACGTCACCACTGAAGTCAAGTCCGTTGAGATGCACCAC

GAGCAGCTCCCCGAGGGTTTCCCCGGTGACAACGTTGGTTTCAACGTGAAGAACGTTTCC

GTCAAGGATATCCGCCGTGGTAACGTCGCTTCTGACTCCAAGAACGACCCCGCTCTCGGT

GCCGCTTCTTTCGACGCCCAGGTCATTGTCCTGAACCACCCTGGTCAGGTCGGTGCTGGT

TACGCTCCCGTCCTGGACTGCCACACTGCCCAC-ATTGCCTGCAAGTTCGCCGAGATCAA

GGAGAAGATCGACCGTCGTACTGGTAAGGCTGTTGAGGCTGCCCCCAAGTTCATCAAGTC

TGGTGACT

>Stanjemonium_spectabile_CF278320 Aphanocladium VG its_28s......

ACT-GAGTTT---AC--ACAAACTCCC-AACCCTATGTGAACC-TTACCTTTACC-GTTG

CTTCGGCGGG--CCGCCCC-GGGTGCT---GC-----------GT--GCCCCGGAC-CCA

-GGCGCCCGCCGGGG-AC-ACCCAAACTCT--GTATTT---ACCA--AGTGTAT-CTTCT

GAG-CCGCCGAAAGGCG--AAAACAAAACGAA--TCAAAACTTTCAACAACGGATCTCTT

GGTTCTGGCATCGATGAAGAACGCAGCGAAATGCGATAAGTAATGTGAATTGCAGAATTC

AGTGAATCATCGAATCTTTGAACGCACATTGCGCCCGCCAGTATTCTGGCGGGCATGCCT

GTCCGAGCGTCATTTCAACCCTCGGGCCCCCCCTTTTCCCCTTCACGGGGGACGGGGGCG

GGCC-CGGTGTTGGGGCTCAGGC------GCC-GCTAAGCC-----GGCCGC-CTGTCCC

C-TAAATCCAGTGGCGGCCTCGCCGCTGCC-TCCTCCGCGTAGTAGC--ACAAACCTC--

GCGGTTGGAA--GGCGGCGCGGCCACGCCGTAAAACCCCCG--ACTTTTTACCAA-AGTA

ACGGCGA-GTGAAGCGGCAACAGCTCAAATT-TGAAAGCT-GGC--TCT-CGGGCCCGCA

-TTGTAATTTGCAGAGGATGCTTCTGGCGACGCGCCTTCCGAGTTCCCTGG-AACGGGAC

GCCATAGAGGGTGAGAGCCCCGTCCGGTCGTGCGCC-T-AGCCTCTGTGAAGCTCCTTCG

ACGAGTCGAGTAGTTTGGGAATGCTGCTCAAAATGGGAGGTATACGTCTTCTAAAGCTAA

ATATTGGCCAGAGACCGATAGCGCACAAGTAGAGTGATCGAAAGATGAAAAGCACTTTGA

AAAGAGAGTTAAAAAGTACGTGAAATTGTTGAAAGGGAAGCGCTTATGACCAGACTTGGG

CTGGCCCGATCATCCGGCGTTCTCGCCGGTGCACTC-GGGCCGCT-CAGGCCAGCATCAG

CTTGCGCCGGGGGATAAAGGCGGCGGGAATGTGGCTCC-TCCT---GGAGTGTTATAGCC

CGCCGCGCAATACCCTGGTGCGGGCTGAGGT-CGCGCA----------------------

--------------------CGTCAACTACACAACACGCACTGGGGCCTAGTCTGTCCTG

CGGAAACCCCCGAGGGTCAAGCTTGCGGCCTCGTCAAGAATCTCTCTCTTATGTGCTACG

TTAGTGTTGGATCACCGGCCGATCCCCTGGTAGATTTCATGATCAACAGAGGTATGGAAG

TCATCGAAGAATATGAGCCGCTGAGGTTTCCGCATGCCACCAAGATCTTCATCAACGGCA

GCTGGGTAGGGGTTCACCAGGACCCTAAGCATCTTGTCAACCAGGTACTTGACTTGCGTC

GCAAGTCCTATCTCAACTATGAGGTTTCGCTCGTTCGCGACATCAGAGACCGAGAATTTA

AAATCTTTTCTGATGCGGGCCGTGTAATGAGGCCAGTTTTTACAGTGCGACAGGAAGATA

GTCCAGAT-GGGGAATATCCTAGAGGATCTCTTGTCCTCACGAAGGAACATGTCAACCAG

CTTGCAAGGGACCAATCTGGGGAGCCCCCAGAGCCCAAC-GAGGATGGCGATGTTAAGCC

AGGCTGGGAGGGGTTGGTGAGTGCGGGTGCTGTTGAGTATCTCGACGCCGAGGAAGAAGA

AACAGCTATGATTTGCATGACTCCTGATGACCTCGAAGTTTATCGTGCTCAGAAAACCGC

GCAAAAGAACGCTCAAAATAGTGGACAAAGCGGCTCAGAACCATTCCTACAACCCGCTTT

TGATCCAGCTGAACCCAACAAGCGTATCAGAACCAGAATCAATGCATCGACGCATATCTA

TACTCATTGCGAGATTCACCCGAG-----TCCAAGGATGGCCAGACCCGTGAGCACGCTC

TGCTTGCCTTCACCCTTGGTGTGAAGCAGCTCATCGTTGCCATCAACAAGATGGACACTG

CC-AAGTGGGCCGAGGCTCGTTTCAACGAGATTATCAAGGAGA--CCACTTCCTTCATCA

AGAAGGTCGGCTTCAACCCCAAGAACGTTGCCTTCGTCCCCATCTCTGGCTTCAACGGCG

ACAACATGCTTGAGC----CCTCCAGCAACTGCCCCTGGTACAAGGGTTGGGAGAAGGAG

ATCAAGGGTGG---CAAGGCTACCGGCAAGACCCTCCTCGAGGCCATTGACGCCATTGAG

CCCCCCAAGCGTCCTACCGACAAGCCCCTCCGTCTCCCTCTCCAGGATGTGTACAAGATT

GGTGGTATCGGCACAGTTCCTGTCGGCCGTATCGAGACTGGTATCATCAAGCCCGGTATG

GTCGTTACCTTCGCTCCTGCCAACGTCACCACTGAAGTCAAGTCCGTCGAGATGCACCAC

GAGCAGCTCACCGAGGGTCTCCCCGGTGACAACGTTGGTTTCAACGTGAAGAACGTGTCC

GTTAAGGATATTCGCCGTGGCAACGTCGCCTCTGACTCCAAGAACGACCCCGCCATGGGC

GCCGCTTCCTTCGACGCCCAGGTCATCATTCTCAACCACCCCGGTCAGGTCGGTGCCGGT

TACGCTCCCGTCCTCGACTGCCACACTGCCCAC-ATTGCTTGCAAGTTCGCCGAGATCAA

GGAGAAGATTGACCGTCGTACTGGTAAGTCTACCGAGTCGAACCCCAAGTTCGTCAAGTC

CGGTGACG

>Stanjemonium_grisellum_CBS100389_CF241431 ......

ACT-GAGTTA---TC---C-AACTCCCAAACCC-CTGTGAAC--ATACC--TAC--GTTG

CTTCGGCGGG--CCGTCCCGCGGCGC----GC-CCACGTGG-CGT--GACCCGGAC-CCA

-GGCGCCCGCCGGGG-ACC-CCCAAACTCTT-GTTTT-----CCA---GTGTCT-CCTCT

GAGTGG--CATAA-GC------A-AAAA-TAAA--CAAAACTTTCAGCAACGGATCTCTT

GGTTCTGGCATCGATGAAGAACGCAGCGAAATGCGATAAGTAATGTGAATTGCAGAATTC

AGTGAATCATCGAATCTTTGAACGCACATTGCGCCCGCCAGTATTCTGGCGGGCATGCCT

GTCTGAGCGTCATTTCAACCCTCAG-CCCCCG------CT---CGCGGGG--------CG

----CTGGCGTTGGGGAT-CGGCC-----GTCCTC------G----CGGCGG-CCGGCCC

C-GAAACACAGTGGCGGTCTC-TCGCGGAC-TCCCCTGCGTAGTAGC--ACT-ACCTC--

GCAGAAGGGACGAGCGGGCTGACCACGCCGTAAAACCCCCC--ACT-TCT-CCA--AGTA

ACGGCGA-GTGAAGCGGCAACAGCTCAAATT-TGAAATCT-GGCC-TT---GTGCCCG-A

GTTGTAATTTGTAGAGGATGCTTCTGGCGACGCGCCTTCCGAGTTCCCTGG-AACGGGAC

GCCATAGAGGGTGAGAGCCCCGTCCGGTCGTGCGCC-T-AGCCTCTGTGAAGCTCCTTCG

ACGAGTCGAGTAGTTTGGGAATGCTGCTCTAAATGGGAGGTATACGTCTTCTAAAGCTAA

ATACCGGCCAGAGACCGATAGCGCACAAGTAGAGTGATCGAAAGATGAAAAGCACTTTGA

AAAGAGGGTTAAATAGTACGTGAAATTGCTGAAAGGGAAGCGCTTATGACCAGACTTGGG

CGCGGCGGATCATCCGGCGTTCTCGCCGGTGCACTCCA--CCGCCCCAGGCCAGCATCAG

CTCGCGCCGGGGGACAAAGGCTTCGGGAATGTGGCTCC-CTC---GGGAGTGTTATAGCC

CGTTGCGTAATACCCTGGTGCGGGCTGAGGTCCGCGC-TCTGCAAGGATGCTGGCGTAAT

GGTCATCAGTGACCCGTCTTCGACAGCTTCACAATACTCACTGGGGCCTCGTCTGTCCTG

CGGAAACCCCCGAGGGCCAGGCTTGCGGCCTTGTCAAGAACCTGTCACTGATGTGCCATG

TTTCAGTTGGCACTCCTGCTGATCCCCTTTACAATTTCTTCATCAGCAGGGGCATGGAAG

TCCTCGAAGAGTACGAGCCAAAACGCTTTCCCAACTCGACCAAAGTCTTCCTCAACGGCA

GCTGGGTAGGAGTACACGAGAACCCGAGAGAGCTTGTGGACCACCTTGTGGCAATGCGCC

GCAGTGGGGGTATAAGCGAAGAAGTGTCCCTTATCCGCGACATCCGCGATCGTGAATTCA

AGTTCTTCTCGGATGCCGGTCGCGTTATGCGTCCTTTGCTCACCGTACAGCAACATGATG

GCG-AGATTGGCCAACCAGAGAAAGGCTCCCTCTGCCTGACCAAGGAGCACGTGTCCGGA

CTCTCCGAGATGCATG-AGGTGGACAATGGACA-------AGAGCTGATGGAGGTGA--C

TGGCTGGAAGTGGCTCGTGAAGAGTGGCTGCATCGAATACCTCGATGCTGAGGAGGAAGA

AACTGCCATGATATGCATGACCCCTGATGATCTGGAACTCTACCGCCAACAGAAG-----

----------------------------CTTGGCTACAATGTGGAT------GAGG---A

CCCGGCTTTGGAGCCAAATAAGCGCCTGCGTACCAAGACAAACCCTACGACACATATGTA

CACGCACTGTGAGATTCATCCTAGTATGATCCAAGGATGGCCAGACCCGCGAGCACGCCC

TGCTGGCCTACACCCTTGGTGTCCGCCAGATCATTGTCGCCATTAACAAGATGGACACTG

CC-AAGTGGGCCGAGGCCCGTTACCAGGAGATTGTCAAGGAGA--CCTCCACCTTCATCA

AGAAGGTCGGCTACAACCCCAAGACCGTCCCCTTCGTCCCCATCTCCGGCTTCAACGGTG

ACAACATGTTGGCCC----CCTCCACCAACTGCCCCTGGTACAAGGGTTGGGAGAAGGAG

ACCAAGGCTGG---CAAGTCCAGTGGCAAGACCCTGCTCGAGGCCATCGACTCCATCGAG

CCCCCCAAGCGTCCCTCCGACAAGCCCCTCCGTCTTCCCCTCCAGGACGTGTACAAGATC

GGTGGTATTGGCACGGTGCCCGTCGGCCGTATCGAGACCGGTGTCTTGAAGCCCGGCATG

GTCGTCACCTTTGCCCCCGCCGGTGTCACCACTGAAGTCAAGTCCGTCGAGATGCACCAC

GAGCAGCTGACCGAGGGTCTCCCCGGTGACAACGTTGGTTTCAACGTCAAGAACGTGTCC

GTCAAGGACATTCGCCGTGGTAACGTCGCCTCCGACTCCAAGAACGACCCCGCCATGGGT

GCCGCTTCCTTCGATGCCCAGGTCATCGTCCTCAACCACCCCGGTCAGGTCGGTGCCGGC

TACGCCCCCGTCCTCGACTGCCACACCGCCCAC-ATTGCCTGCAAGTTCGCTGAGCTCAA

GGAGAAGATCGACCGCCGTACCGGCAAGTCGACCGAGGAGGCCCCCAAGTTCATCAAGTC

TGGTGACT

>Stanjemonium_grisellum_CBS65579 Stanjemonium grisellum CBS 655.79 ITS region; from TYPE material.....

ACT-GAGTTA---TC---C-AACTCCCAAACCC-CTGTGAAC--ATACC--TAC--GTTG

CTTCGGCGGG--CCGTCCCGCGGCGC----GC-CCACGTGG-CGT--GACCCGGAC-CCA

-GGCGCCCGCCGGGG-ACC-CCCAAACTCTT-GTTTT-----CCA---GTGTCT-CCTCT

GAGTGG--CATAA-GC------A-AAAA-TAAA--CAAAACTTTCAGCAACGGATCTCTT

GGTTCTGGCATCGATGAAGAACGCAGCGAAATGCGATAAGTAATGTGAATTGCAGAATTC

AGTGAATCATCGAATCTTTGAACGCACATTGCGCCCGCCAGTATTCTGGCGGGCATGCCT

GTCTGAGCGTCATTTCAACCCTCAG-CCCCCG------CT---CGCGGGG--------CG

----CTGGCGTTGGGGAT-CGGCC-----GTCCTC------G----CGGCGG-CCGGCCC

C-GAAACACAGTGGCGGTCTC-TCGCGGAC-TCCCCTGCGTAGTAGC--ACT-ACCTC--

GCAGAAGGGACGAGCGGGCTGACCACGCCGTAAAACCCCCC--ACT-TCT-CCA------

--GGCGA-GTGAAGCGGCAACAGCTCAAATT-TGAAATCT-GGCC-TT---GTGCCCG-A

GTTGTAATTTGTAGAGGATGCTTCTGGCGACGCGCCTTCCGAGTTCCCTGG-AACGGGAC

GCCATAGAGGGTGAGAGCCCCGTCCGGTCGTGCGCC-T-AGCCTCTGTGAAGCTCCTTCG

ACGAGTCGAGTAGTTTGGGAATGCTGCTCTAAATGGGAGGTATACGTCTTCTAAAGCTAA

ATACCGGCCAGAGACCGATAGCGCACAAGTAGAGTGATCGAAAGATGAAAAGCACTTTGA

AAAGAGGGTTAAATAGTACGTGAAATTGCTGAAAGGGAAGCGCTTATGACCAGACTTGGG

CGCGGCGGATCATCCGGCGTTCTCGCCGGTGCACTCCA--CCGCCCCAGGCCAGCATCAG

CTCGCGCCGGGGGACAAAGGCTTCGGGAATGTGGCTCC-CTC---GGGAGTGTTATAGCC

CGTTGCGTAATACCCTGGTGCGGGCTGAGGTCCGCGC-TCTGCAAGGATGCTGGCGTAAT

GGTCATCAGTGACCCGTCTTCGACAGCTTCACAATACTCACTGGGGCCTCGTCTGTCCTG

CGGAAACCCCCGAGGGCCAGGCTTGCGGCCTTGTCAAGAACCTGTCACTGATGTGCCATG

TTTCAGTTGGCACTCCTGCTGATCCCCTTTACAATTTCTTCATCAGCAGGGGCATGGAAG

TCCTCGAAGAGTACGAGCCAAAACGCTTTCCCAACTCGACCAAAGTCTTCCTCAACGGCA

GCTGGGTAGGAGTACACGAGAACCCGAGAGAGCTTGTGGACCACCTTGTGGCAATGCGCC

GCAGTGGGGGTATAAGCGAAGAAGTGTCCCTTATCCGCGACATCCGCGATCGTGAATTCA

AGTTCTTCTCGGATGCCGGTCGCGTTATGCGTCCTTTGCTCACCGTACAGCAACATGATG

GCG-AGATTGGCCAACCAGAGAAAGGCTCCCTCTGCCTGACCAAGGAGCACGTGTCCGGA

CTCTCCGAGATGCATG-AGGTGGACAATGGACA-------AGAGCTGATGGAGGTGA--C

TGGCTGGAAGTGGCTCGTGAAGAGTGGCTGCATCGAATACCTCGATGCTGAGGAGGAAGA

AACTGCCATGATATGCATGACCCCTGATGATCTGGAACTCTACCGCCAACAGAAG-----

----------------------------CTTGGCTACAATGTGGAT------GAGG---A

CCCGGCTTTGGAGCCAAATAAGCGCCTGCGTACCAAGACAAACCCTACGACACATATGTA

CACGCACTGTGAGATTCATCCTAGTATGATCCAAGGATGGCCAGACCCGCGAGCACGCCC

TGCTGGCCTACACCCTTGGTGTCCGCCAGATCATTGTCGCCATTAACAAGATGGACACTG

CC-AAGTGGGCCGAGGCCCGTTACCAGGAGATTGTCAAGGAGA--CCTCCACCTTCATCA

AGAAGGTCGGCTACAACCCCAAGACCGTCCCCTTCGTCCCCATCTCCGGCTTCAACGGTG

ACAACATGTTGGCCC----CCTCCACCAACTGCCCCTGGTACAAGGGTTGGGAGAAGGAG

ACCAAGGCTGG---CAAGTCCAGTGGCAAGACCCTGCTCGAGGCCATCGACTCCATCGAG

CCCCCCAAGCGTCCCTCCGACAAGCCCCTCCGTCTTCCCCTCCAGGACGTGTACAAGATC

GGTGGTATTGGCACGGTGCCCGTCGGCCGTATCGAGACCGGTGTCTTGAAGCCCGGCATG

GTCGTCACCTTTGCCCCCGCCGGTGTCACCACTGAAGTCAAGTCCGTCGAGATGCACCAC

GAGCAGCTGACCGAGGGTCTCCCCGGTGACAACGTTGGTTTCAACGTCAAGAACGTGTCC

GTCAAGGACATTCGCCGTGGTAACGTCGCCTCCGACTCCAAGAACGACCCCGCCATGGGT

GCCGCTTCCTTCGATGCCCAGGTCATCGTCCTCAACCACCCCGGTCAGGTCGGTGCCGGC

TACGCCCCCGTCCTCGACTGCCACACCGCCCAC-ATTGCCTGCAAGTTCGCTGAGCTCAA

GGAGAAGATCGACCGCCGTACCGGCAAGTCGACCGAGGAGGCCCCCAAGTTCATCAAGTC

TGGTGACT

>Stanjemonium_ochroroseum_CBS65679T Stanjemonium ochroroseum CBS 656.79 ITS region; from TYPE material.....

ACT-GAGTTA---TC---C-AAATCCCAAACCC-CTGTGAAC--ATACC--TAC--GTTG

CTTCGGCGGG--CCGTCCCGCGGCGC----GC-CCACGTGG-CGT--GACCCGGAC-CCA

-GGCGCCCGCCGGGG-ACC--CCAAACTCTT-GTTTT-----CCA---GTGTCT-CCTCT

GAGTGG--CATAA-GC------A-AAAA-TAAA--CAAAACTTTCAGCAACGGATCTCTT

GGTTCTGGCATCGATGAAGAACGCAGCGAAATGCGATAAGTAATGTGAATTGCAGAATTC

AGTGAATCATCGAATCTTTGAACGCACATTGCGCCCGCCAGTATTCTGGCGGGCATGCCT

GTCTGAGCGTCATTTCAACCCTCAG-CCCCCG------CT---CGCGGGG--------CG

----CTGGCGTTGGGGAT-CGGCC-----GTCCTC------G----CGGCGG-CCGGCCC

C-GAAACACAGTGGCGGTCTC-TCGCGGAC-TCCCCTGCGTAGTAGC--ACT-ACCTC--

GCAGAAGGGACGAGCGGGCTGACCACGCCGTAAAACCCCC---ACT-TCT-CCA------

--GGCGA-GTGAAGCGGCAACAGCTCAAATT-TGAAATCT-GGCC-TC---GTGCCCG-A

GTTGTAATTTGTAGAGGATGCTTCTGGCGACGCGCCTTCCGAGTTCCCTGG-AACGGGAC

GCCATAGAGGGTGAGAGCCCCGTCCGGTCGTGCGCC-T-AGCCTCTGTGAAGCTCCTTCG

ACGAGTCGAGTAGTTTGGGAATGCTGCTCTAAATGGGAGGTATACGTCTTCTAAAGCTAA

ATACCGGCCAGAGACCGATAGCGCACAAGTAGAGTGATCGAAAGATGAAAAGCACTTTGA

AAAGAGGGTTAAATAGTACGTGAAATTGCTGAAAGGGAAGCGCTTATGACCAGACTTGGG

CGCGGCGGATCATCCGGCGTTCTCGCCGGTGCACTCCA--CCGCCCCAGGCCAGCATCAG

CTCGCGCCGGGGGACAAAGGCTTCGGGAATGTGGCTCC-CTC---GGGAGTGTTATAGCC

CGTTGCGTAATACCCTGGTGCGGGCTGAGGTCCGCGC-TCTGCAAGGATGCTGGCGTAAT

GGTCATCAGTGACCCGTCTTCGACAGCTTCACAATACTCACTGGGGCCTCGTCTGTCCTG

CGGAAACCCCTGAGGGCCAGGCTTGCGGCCTCGTCAAGAACCTGTCACTGATGTGCCATG

TTTCAGTTGGCACTCCTGCTGATCCACTTTACAATTTCTTCATCAGCAGGGGCATGGAAG

TCCTCGAAGAGTACGAGCCGAAACGCTTTCCCAACTCGACCAAAGTCTTCCTCAACGGCA

GCTGGGTAGGAGTACACGAGAACCCGAGAGAGCTTGTGGACCACCTTGTGGCAATGCGCC

GCAGTGGAGGTATAAGCGAAGAGGTGTCCCTCATCCGCGACATCCGCGATCGTGAATTCA

AGTTCTTCTCGGATGCCGGTCGCGTTATGCGTCCTTTGCTCACCGTGCAGCAACATGATG

GCG-AGATTGGCCAACCAGAGAAAGGCTCCCTCTGCCTGACCAAGGAGCACGTGTCCGGA

CTCTCCGAGATGCATG-AGGTGGACAATGGACA-------AGAGCTGATGGAGGTGA--C

CGGCTGGAAGTGGCTCGTGAAGAGTGGCTGCATCGAATACCTCGATGCTGAGGAGGAAGA

GACTGCCATGATATGCATGACCCCTGATGATTTGGAACTCTACCGCCAACAGAAG-----

----------------------------CTTGGCTACAATGTGGAC------GAGG---A

CCCAGCTTTGGAGCCAAATAAGCGCCTGCGTACCAAGACAAACCCTACGACACATATGTA

CACGCACTGTGAGATTCATCCTAGTATGATCCAAGGATGGCCAGACCCGCGAGCACGCCC

TGCTGGCCTACACCCTTGGTGTCCGTCAGATCATCGTCGCCATCAACAAGATGGACACCG

CC-AAGTGGGCCGAGGCCCGTTACCAGGAGATCGTCAAGGAGA--CCTCCACCTTCATCA

AGAAGGTCGGCTACAACCCCAAGACCGTCCCCTTCGTCCCCATTTCCGGCTTCAACGGTG

ACAACATGTTGGCCG----CCTCCACCAACTGCCCCTGGTACAAGGGCTGGGAGAAGGAG

ACCAAGGCTGG---CAAGTCCACTGGCAAGACCCTGCTTGAGGCCATCGACTCCATCGAG

CCCCCCAAGCGTCCCTCCGACAAGCCCCTCCGCCTTCCCCTCCAGGATGTGTACAAGATC

GGTGGTATTGGCACGGTGCCCGTCGGCCGTATCGAGACCGGTGTCTTGAAGCCCGGCATG

GTCGTCACCTTTGCCCCCGCTGGTGTCACCACTGAAGTCAAGTCCGTCGAGATGCACCAC

GAGCAGCTGACCGAGGGTCTCCCCGGTGACAACGTTGGTTTCAACGTCAAGAACGTGTCC

GTCAAGGACATTCGCCGTGGTAACGTCGCCTCCGACTCCAAGAACGACCCCGCCATGGGT

GCCGCTTCCTTCGACGCCCAGGTCATCGTCCTCAACCACCCCGGTCAGGTCGGTGCCGGC

TACGCCCCCGTCCTCGACTGCCACACCGCCCAC-ATTGCCTGCAAGTTCGCTGAGCTCAA

GGAGAAGATCGACCGCCGTACCGGCAAGTCGACCGAGGAGGCCCCCAAGTTCATCAAGTC

TGGTGACT

>Stanjemonium_fuscescens_CBS26496T Stanjemonium fuscescens CBS 264.96 ITS region; from TYPE material.....

ACT-GAGTTA---TC---C-AACTCCCAAACCC-CTGTGAAC--ATACC--TAC--GTTG

CTTCGGCGGG--CCGTCCCGCGGCGC----GC-CCACGTGG-CGT--GACCCGGAC-CCC

-GGCGCCCGCCGGGG-ACC--CCAAACTCTT-GTTTT-----CCA---GTGTCT-CCTCT

GAGTGG--CATAA-GC------A-AAAA-TAAA--CAAAACTTTCAGCAACGGATCTCTT

GGTTCTGGCATCGATGAAGAACGCAGCGAAATGCGATAAGTAATGTGAATTGCAGAATTC

AGTGAATCATCGAATCTTTGAACGCACATTGCGCCCGCCAGTATTCTGGCGGGCATGCCT

GTCTGAGCGTCATTTCAACCCTCAG-CCCCCG------CT---CGCGGGG--------CG

----CTGGCGTTGGGGCT-CGGCC-----GTCCTC------G----CGGCGG-CCGGCCC

C-GAAACACAGTGGCGGTCTC-TCGCGGAC-TCCCCTGCGTAGTAAC--ACT-ACCTC--

GCAGAAGGGACGAGCGGGCGGACCACGCCGTAAAACCCCCC--ACT-TCT-CCA------

--GGCGA-GTGAAGCGGCAACAGCTCAAATT-TGAAATCT-GGCC-TT---GTGCCCG-A

GTTGTAATTTGTAGAGGATGCTTCTGGCGACGCGCCTTCCGAGTTCCCTGG-AACGGGAC

GCCATAGAGGGTGAGAGCCCCGTCCGGTCGTGCGCC-T-AGCCTCTGTGAAGCTCCTTCG

ACGAGTCGAGTAGTTTGGGAATGCTGCTCTAAATGGGAGGTATACGTCTTCTAAAGCTAA

ATACCGGCCAGAGACCGATAGCGCACAAGTAGAGTGATCGAAAGATGAAAAGCACTTTGA

AAAGAGGGTTAAATAGTACGTGAAATTGCTGAAAGGGAAGCGCTTATGACCAGACTTGGG

CGCGGCGGATCATCCGGCGTTCTCGCCGGTGCACTCCA--CCGCCCCAGGCCAGCATCAG

CTCGCGCCGGGGGACAAAGGCTTCGGGAATGTGGCTCC-CTC---GGGAGTGTTATAGCC

CGTTGCGTAATACCCTGGTGCGGGCTGAGGTCCGCGC-TCTGCAAGGATGCTGGCGTAAT

GGTCATCAGTGACCCGTCTTCGACAGCTTCACAATACTCACTGGGGCCTCGTCTGTCCTG

CGGAAACCCCCGAGGGCCAGGCTTGTGGCCTTGTCAAGAATCTGTCACTGATGTGCCATG

TTTCGGTTGGCACTCCTGCTGATCCACTTTACAATTTCTTCATCAGCAGGGGCATGGAAG

TCCTCGAAGAGTACGAGCCGAAACGGTTTCCCAACTCAACCAAGGTCTTCCTCAACGGCA

GCTGGGTAGGAGTACACGAGAACCCAAGAGAGCTCGTGGACCACCTTGTGGCAATGCGCC

GTAGTGGGGGTATAAGCGAAGAGGTGTCCCTCATCCGCGACATCCGCGATCGTGAATTCA

AGTTCTTCTCGGATGCCGGTCGCGTCATGCGTCCTTTGCTTACCGTGCAGCAACATGATG

GCG-AGATTGGCCAGCCGGAGAAAGGCTCCCTCTGCCTGACCAAGGAGCACGTGTCCGGA

CTCTCCGAGATGCATG-AGGTGGACAATGGACA-------AGAGCTGATGGAGGTGA--C

TGGCTGGAAGTGGCTCGTGAAGAGTGGCTGCATCGAATACCTCGATGCTGAGGAAGAAGA

AACTGCCATGATATGCATGACCCCTGATGATCTGGAACTCTACCGCCAACAGAAG-----

----------------------------CTTGGCTACAATGTGGAC------GAGG---A

CCCGGCTTTGGAGCCAAATAAGCGCCTGCGTACCAAGACAAACCCTACGACACATATGTA

CACGCACTGTGAGATTCATCCTAGTATGATCCAAGGATGGCCAGACCCGTGAGCACGCCC

TGCTCGCCTACACCCTTGGTGTCCGCCAGATCATTGTCGCCATCAACAAGATGGACACTG

CC-AAGTGGGCCGAGTCCCGCTACCAGGAGATCGTCAAGGAGA--CCTCCACCTTCATCA

AGAAGGTCGGCTACAACCCCAAGACCGTCCCCTTCGTCCCCATCTCCGGCTTCAACGGTG

ACAACATGTTGGCCG----CCTCCACCAACTGCCCCTGGTACAAGGGTTGGGAGAAGGAG

ACCAAGGCTGG---CAAGTCCACCGGCAAGACCCTGCTGGAGGCCATCGACTCCATCGAG

CCCCCCAAGCGTCCCTCCGACAAGCCCCTCCGTCTCCCCCTCCAGGATGTGTACAAGATC

GGTGGTATTGGCACGGTGCCCGTCGGCCGTATCGAGACTGGTGTCTTGAAGCCCGGCATG

GTCGTCACCTTCGCCCCCGCTGGTGTCACCACTGAAGTCAAGTCCGTCGAGATGCACCAC

GAGCAGCTGACCGAGGGTCTCCCCGGTGACAACGTTGGTTTCAACGTCAAGAACGTGTCC

GTCAAGGACATCCGCCGTGGTAACGTCGCCTCCGACTCCAAGAACGACCCCGCCATGGGT

GCCGCTTCTTTCGATGCCCAGGTCATCGTCCTCAACCACCCCGGTCAGGTCGGTGCCGGC

TACGCCCCCGTCCTCGACTGCCACACCGCCCAT-ATTGCCTGCAAGTTCGCTGAGCTCAA

GGAGAAGATCGACCGCCGTACCGGCAAGTCGACTGAGGAGGCCCCCAAGTTCATCAAGTC

TGGTGACT

>Stanjemonium_dichromosporum_CBS63873T Stanjemonium dichromosporum CBS 638.73 ITS region; from TYPE material.....

ACT-GAGTTA---TC---C-AACTCCCAAACCC-CTGTGAAC--ATACC--TAC--GTTG

CTTCGGCGGG--CCGTCCCGCGGCGC----GC-CCACGTGG-CGT--GACCCGGAC-CCA

-GGCGCCCGCCGGGG-ACC--CCAAACTCTT-GTTTT-----CCA---GTGTCT-CCTCT

GAGTGG--CATAA-GC------A-AAAA-TAAA--CAAAACTTTCAGCAACGGATCTCTT

GGTTCTGGCATCGATGAAGAACGCAGCGAAATGCGATAAGTAATGTGAATTGCAGAATTC

AGTGAATCATCGAATCTTTGAACGCACATTGCGCCCGCCAGTATTCTGGCGGGCATGCCT

GTCTGAGCGTCATTTCAACCCTCAG-CCCCCG------CT---CGCGGGG--------CG

----CTGGCGTTGGGGAT-CGGCC-----GTCCTC------G----CGGCGG-CCGGCCC

C-GAAACACAGTGGCGGTCTC-CCGCGGAC-TCCCCTGCGTAGTAGC--ACT-ACCTC--

GCAGAAGGGACGAGCGGGCTGGCCACGCCGTAAAACCCCCC--ACT-TCT-CAA------

--GGCGA-GTGAAGCGGCAACAGCTCAAATT-TGAAATCT-GGCC-TC---GTGCCCG-A

GTTGTAATTTGTAGAGGATGCTTCTGGCGACGCGCCTTCCGAGTTCCCTGG-AACGGGAC

GCCATAGAGGGTGAGAGCCCCGTCCGGTCGTGCGCC-T-AGCCTCTGTGAAGCTCCTTCG

ACGAGTCGAGTAGTTTGGGAATGCTGCTCTAAATGGGAGGTATACGTCTTCTAAAGCTAA

ATACCGGCCAGAGACCGATAGCGCACAAGTAGAGTGATCGAAAGATGAAAAGCACTTTGA

AAAGAGGGTTAAATAGTACGTGAAATTGCTGAAAGGGAAGCGCTTATGACCAGACTTGGG

CGCGGCGGATCATCCGGCGTTCTCGCCGGTGCACTCCA--CCGCCCCAGGCCAGCATCAG

CTCGCGCCGGGGGACAAAGGCTTCGGGAATGTGGCTCC-CTC---GGGAGTGTTATAGCC

CGCTGCGTAATACCCTGGTGCGGGCTGAGGTCCGCGC-TCTGCAAGGATGCTGGCGTAAT

GGTCATCAGTGACCCGTCTTCGACAGCTTCACAATACTCACTGGGGCCTCGTCTGTCCTG

CGGAAACCCCCGAGGGCCAGGCTTGCGGCCTTGTCAAGAACCTGTCACTGATGTGCCATG

TTTCGGTTGGCACTCCTGCTGATCCCCTTTACAATTTCTTCATCAGCAGGGGCATGGAAG

TCCTCGAAGAGTACGAGCCGAAACGGTTTCCCAACTCAACCAAAGTCTTCCTCAACGGCA

GCTGGGTAGGAGTACACGAGAACCCCAGAGAGCTCGTGGACCACCTTGTGGCAATGCGCC

GCAGTGGGGGTATAAGCGAAGAGGTGTCCCTCATCCGCGACATCCGCGATCGTGAATTCA

AGTTCTTCTCGGATGCCGGTCGCGTCATGCGTCCTTTACTCACCGTGCAGCAACATGATG

GCG-AGATTGGCCAGCCAGAGAAAGGCTCCCTCTGCCTGACCAAGGAGCACGTGTCCGGA

CTCTCCGAGATGCATG-AGGTGGACAATGGACA-------AGAGCTGATGGAGGTGA--C

TGGCTGGAAGTGGCTCGTGAAGAGTGGCTGCATCGAATACCTCGATGCTGAGGAAGAAGA

AACTGCCATGATATGCATGACCCCTGATGATCTGGAACTCTACCGCCAACAGAAG-----

----------------------------CTTGGCTACAATGTGGAC------GAGG---A

CCCGGCTTTGGAGCCAAATAAGCGCCTGCGTACCAAGACAAACCCTACGACACATATGTA

CACGCACTGTGAGATTCATCCTAGTATGATCCAAGGATGGCCAGACCCGTGAGCACGCCC

TGCTCGCCTACACCCTTGGTGTCCGCCAGATCATTGTCGCCATCAACAAGATGGACACTG

CC-AAGTGGGCCGAGGCTCGTTACCAGGAGATCGTCAAGGAGA--CCTCGACCTTCATCA

AGAAGGTCGGCTACAACCCCAAGACCGTCCCCTTCGTCCCCATCTCCGGCTTCAACGGTG

ACAACATGTTGGCTC----CCTCCACCAACTGCCCCTGGTACAAGGGTTGGGAGAAGGAG

ACCAAGGCTGG---CAAGTCCTCTGGCAAGACCCTGCTTGAGGCCATCGACTCCATCGAG

CCCCCCAAGCGTCCCTCCGACAAGCCCCTCCGTCTTCCCCTCCAGGATGTGTACAAGATC

GGTGGTATTGGCACGGTGCCCGTCGGCCGTATCGAGACTGGTACCTTGAAGCCCGGCATG

GTCGTCACCTTCGCCCCCGCTGGTGTCACCACTGAAGTCAAGTCCGTCGAGATGCACCAC

GAGCAGCTGACCGAGGGTCTCCCTGGTGACAACGTTGGTTTCAACGTCAAGAACGTGTCC

GTCAAGGACATTCGCCGTGGTAACGTCGCCTCCGACTCCAAGAACGACCCCGCCATGGGT

GCCGCTTCTTTCGATGCCCAGGTCATCGTCCTCAACCACCCCGGCCAGGTCGGTGCCGGC

TACGCCCCCGTCCTCGACTGCCACACCGCCCAC-ATTGCCTGCAAGTTCGCTGAGCTCAA

GGAGAAGATCGACCGCCGTACCGGCAAGTCGACCGAGGAGTCCCCCAAGTTCATCAAGTC

CGGTGACT

>Emericellopsis_glabra_AY632657_CBS11940 Emericellopsis glabra strain CBS 119.40 internal transcribed spacer 1, 5.8S ribosomal RNA gene, and internal transcribed spacer 2, complete sequence; and 28S ribosomal RNA gene, partial sequence.....

ACT-GAGTTAT--CC-----AACTCCCAAACCC-CTGTGAAC--ATACC--TA-T-GTTG

CTTCGGCGGG--CCGTCCCGCGGCGC----GC-CCACGTGG-CGT--GACC-GGAC-CCA

-GGCGCCCGCCGGG--AA---CCAAACTCTT-GTCTT------C-G-AGTGTCT-CCTCT

GAGTGG--CATAA-GC------A-AAAA-TAAA--CAAAACTTTCAGCAACGGATCTCTT

GGTTCTGGCATCGATGAAGAACGCAGCGAAATGCGATAAGTAATGTGAATTGCAGAATTC

AGTGAATCATCGAATCTTTGAACGCACATTGCGCCCGCCAGTATTCTGGCGGGCATGCCT

GTCTGAGCGTCATTTCAACCCTCAG-CCCCCG----TTC-----GCGGGG--------CG

----CTGGCGTTGGGGCC-CGGCC-----GTCCTC------G----CGGCGG-CCGTCCC

C-GAAACACAGTGGCGGTCTC-CCGCAGAC-TCCCCTGCGTAGTAGC--ACT-ACCTC--

GCAGAAGGGACGAGCGGGCTGGCCACGCCGTAAAACACCCC--ACT-TCT-CCA--AGTA

ACGGCGAAGTGAAGCGGCAACAGCTTCAAATTTGAAATCT-GGCC-TC---GTGCCCG-A

GTTGTAATTTGTAGAGGATGCTTTTGGCGACGCGCCTTCCGAGTTCCCTGG-AACGGGAC

GCCATAGAGGGTGAGAGCCCCGTCCGGTCGTGCGCC-T-AGCCTCTGTAAAGCTCCTTCG

ACGAGTCGAGTAGTTTGGGAATGCTGCTCTAAATGGGAGGTATACGTCTTCTAAAGCTAA

ATACCGGCCAGAGACCGATAGCGCACAAGTAGAGTGATCGAAAGATGAAAAGCACTTTGA

AAAGAGGGTTAAATAGTACGTGAAATTGCTGAAAGGGAAGCGCTTATGACCAGACTTGGG

CGCGGCGGATCATCCGGCGTTCTCGCCGGTGCACTCCA--CCGCCCCAGGCCAGCATCAG

TTCGCGCCGGGGGACAAAGGCTTCGGGAATGTGGCTCT-CTC---GGGAGTGTTATAGCC

CGCTGCGTAATACCCTGGCGCGGACTGAGGTCCGCGC-TCTGCAAGGATGCTGGCGTAAT

GGTCATCAGTGACCCGTCTTCGACAGCTTCACAATACTCATTGGGGCCTTGTCTGCCCTG

CGGAAACCCCCGAGGGTCAGGCCTGTGGCCTTGTGAAGAACCTGTCGCTCATGTGCCATG

TTTCAGTTGGCACTCCCGCCGACCCCTTGTACAATTTCTTCATCAGCAGAGGCATGGAAG

TCCTGGAGGAATACGAACCCAAACGATTCCCCAACTCGACCAAGGTCTTTCTCAACGGCA

GCTGGGTAGGAGTTCACGAGAACCCTCGGGAACTAGTGGATCATCTCGTTGCAATGCGGC

GCAGCGGAGGTATCAGCGAAGAGGTATCGCTCATCCGCGACATCCGTGACCGAGAATTCA

AGTTCTTCTCCGACGCCGGTCGTGTGATGCGTCCCTTGCTCACTGTGCAGCAACAAGATG

GCG-AGATCGGCCAGCCAGAGAAAGGATCCTTGTGCCTTACCAAAGAGCACGTATCCGGA

CTCTCTGAGATGCACG-AGGTGGACAACGGGCA-------AGAATTGATGGAGGTTA--C

TGGCTGGAGGTGGCTCGTGAAGAGTGGCTGTATCGAGTATCTCGACGCTGAGGAAGAAGA

GACTGCAATGATTTGCATGACACCTGATGATCTGGAGCTCTACCGACAACAGAAG-----

----------------------------CTTGGCTACAATGTGGAC------GAGG---A

CCTGGCCGCCGAGCCCAACAAGCGTCTTCGCACGAAGACAAACCCTACGACACATATGTA

CACTCATTGCGAGATTCATCCAAGCATGATCCAAGGATGGCCAGACCCGTGAGCACGCCC

TGCTCGCCTACACCCTCGGTGTCCGTCAGATCATTGTTGCCATCAACAAGATGGACACTG

CC-AAGTGGGCTGAGGCTCGTTACCTTGAGATTGTCAAGGAGA--CCTCCAACTTCATCA

AGAAGGTCGGCTACAACCCCAAGACCGTCCCCTTCGTCCCCATCTCCGGCTTCAACGGCG

ACAACATGTTGACGG----CCTCCACCAACTGCCCCTGGTACAAGGGCTGGGAGAAGGAG

ACCAAGGCCGG---CAAGTCCTCTGGCAAGACCCTCCTCGAGGCCATCGACTCCATCGAG

CCCCCCAAGCGTCCCTCCGACAAGCCCCTCCGCCTTCCCCTCCAGGATGTGTACAAGATC

GGTGGTATCGGCACGGTTCCCGTCGGCCGTATCGAGACTGGTGTCCTGAAGCCCGGCATG

GTCGTCACCTTTGCTCCCGCTGGTGTCACCACTGAAGTCAAGTCCGTCGAGATGCACCAC

GAGCAGCTCACCGAGGGTCTCCCCGGTGACAACGTTGGCTTCAACGTCAAGAACGTGTCC

GTCAAGGACATTCGTCGTGGCAACGTCGCCTCCGACTCCAAGAACGACCCCGCCATGGGT

GCCGCCTCCTTCGACGCCCAGGTCATCGTTCTCAACCACCCTGGCCAGGTCGGTGCTGGC

TACGCCCCCGTCCTCGACTGCCACACCGCCCAC-ATTGCCTGCAAGTTCGCTGAGCTCAA

GGAGAAGATCGACCGCCGTACCGGCAAGTCGACCGAGGAGGCCCCCAAGTTCATCAAGTC

TGGTGACT

>Emericellopsis_minima_AY632669_CBS19055 Emericellopsis minima strain CBS 190.55 internal transcribed spacer 1, 5.8S ribosomal RNA gene, and internal transcribed spacer 2, complete sequence.....

ACT-GAGTTT---AC-----AACTCCCAAACCC-CTGTGAAC--ATACC--TA-T-GTTG

CTTCGGCGGG--CCGTCCCGCGGCGC----GC-CCACGTGG-CGT--GACCCGGAA-CCA

-GGCG-CCGCCGGGG-AC---CCAAACTCTT-GCCTTTT-------TCGTGTCT-CCTCT

GAGTGG--CATAA-GC------A-AAAA-TAAA--CAAAACTTTCAGCAACGGATCTCTT

GGTTCTGGCATCGATGAAGAACGCAGCGAAATGCGATAAGTAATGTGAATTGCAGAATTC

AGTGAATCATCGAATCTTTGAACGCACATTGCCCCCGCCAGTATTCTGGCGGGCATGCCT

GTCTGAGCGTCATTTCAACCCTCAG-CCCCCG----TTC-----GCGGGG--------CG

----CTGGCGTTGGGGATCCGGCC-----GTCCTC------G----CGGCGG-CCGGCCC

C-GAAACGCAGTGGCGGTCTC-T-GCGGAC-TCCCCTGCGTAGTAGC--ACT-ACCTC--

GCAGAAGGGACGAGCGGGCTGGCCACGCCGTAAAACCCCCA--ACT-TCT-CC-------

--GGCGA-GTGAAGCGGCAACAGCTCAAATT-TGAAATCT-GGCACTT---GTGCCCG-A

GTTGTAATTTGTAGAGGATGCTTTTGGCGACGCGCCTTCCGAGTTCCCTGG-AACGGGAC

GCCATAGAGGGTGAGAGCCCCGTCCGGTCGTGCGCC-T-AGCCTCTGTAAAGCTCCTTCG

ACGAGTCGAGTAGTTTGGGAATGCTGCTCTAAATGGGAGGTATACGTCTTCTAAAGCTAA

ATACCGGCCAGAGACCGATAGCGCACAAGTAGAGTGATCGAAAGATGAAAAGCACTTTGA

AAAGAGGGTTAAATAGTACGTGAAATTGCTGAAAGGGAAGCGCTTATGACCAGACTTGGG

CGCGGCGGATCATCCGGCGTTCTCGCCGGTGCACTCCA--CCGCCCCAGGCCAGCATCAG

TTCGCGCCGGGGGACAAAGGCTTCGGGAATGTGGCTCC-CTC---GGGAGTGTTATAGCC

CGTTGCGTAATACCCTGGTGCGGACTGAGGTCCGCGC-TCTGCAAGGATGCTGGCGTAAT

GGTCATCAGTGACCCGTCTTCGACAGCTTCACAATACTCATTGGGGCCTCGTCTGCCCTG

CAGAAACCCCGGAGGGTCAGGCTTGTGGCCTTGTCAAGAACCTGTCGCTGATGTGCCACG

TTTCGGTCGGCACTCCCGCCGATCCCCTGTACAATTTCTTCATCAGCAGAGGCATGGAAG

TACTCGAAGAGTACGAGCCCAAACGGTTCCCGAACTCAACCAAGGTGTTCCTCAACGGCA

GCTGGGTAGGAGTACACGAGAATCCTAGAGAGCTCGTGGATCATCTGGTGGCCATGCGCC

GCAGCGGAGGTATAAGCGAGGAGGTTTCGCTTGTTCGCGACATCCGTGATCGAGAGTTCA

AGTTCTTCTCGGATGCCGGCCGCGTCATGCGCCCTTTGCTCACCGTGCAGCAACACGACG

GCG-AGATTGGCCAGCCAGAGAAGGGCTCCCTCTGCCTGACCAAGGAGCATGTGTCCGGA

CTGTCTGAAATGCACG-AGGTGGACAACGGACA-------AGAGTTGATGGAGGTGA--C

TGGTTGGAAGTGGCTCGTGAAAAGTGGCTGCATCGAGTATCTCGACGCCGAGGAAGAAGA

GACTGCCATGATTTGCATGACTCCCGATGATTTGGAACTCTACCGGCAACAGAAG-----

----------------------------CTGGGCTACAACGTGGAC------GAGG---A

CCTGGCTGCGGAGCCCAATAAGCGCCTGCGGACGAAAACGAATCCCACGACACATATGTA

CACGCATTGCGAGATTCATCCTAGTATGATCCAAGGATGGCCAGACCCGTGAGCACGCCC

TGCTCGCCTACACCCTTGGTGTCCGCCAGATCATCGTTGCCATCAACAAGATGGACACTG

CC-AAGTGGGCTGAGGCTCGTTACCTTGAGATTGTCAAGGAGA--CCTCCACCTTCATCA

AGAAGGTCGGCTACAACCCCAAGACCGTCCCCTTCGTCCCCATCTCCGGCTTCAACGGTG

ACAACATGTTGACTG----CCTCCACCAACTGCCCCTGGTACAAGGGCTGGGAGAAGGAG

ACCAAGGCTGG---CAAGTCCACCGGCAAGACCCTCCTCGAGGCCATTGACGCCATCGAG

CCCCCCAAGCGTCCCTCCGACAAGCCCCTCCGCCTTCCCCTCCAGGATGTGTACAAGATC

GGTGGTATTGGCACGGTTCCCGTCGGCCGTATCGAGACTGGTACCCTGAAGCCCGGCATG

GTCGTCACCTTCGCCCCCGCTGGTGTCACCACTGAAGTCAAGTCCGTCGAGATGCACCAC

GAGCAGCTCACCGAGGGTCTCCCCGGTGACAACGTTGGTTTCAACGTCAAGAACGTGTCC

GTCAAGGACATTCGCCGTGGCAACGTCGCCTCCGACTCCAAGAACGATCCCGCCATGGGT

GCCGCCTCTTTCGATGCCCAGGTCATCGTTCTCAACCACCCTGGTCAGGTCGGTGCCGGT

TACGCTCCCGTCCTCGACTGCCACACCGCCCAC-ATTGCCTGCAAGTTCGCTGAGCTCAA

GGAGAAGATCGACCGCCGTACCGGCAAGTCGACCGAGGAGGCCCCCAAGTTTATCAAGTC

TGGTGACT

>Gliomastix_masseei_AB540553_CBS79469 Gliomastix masseei genes for ITS1, 5.8S rRNA, ITS2 and 28S rRNA, partial sequence, strain: CBS 794.69.....

ACT-GAGTT----GC---AAAACTCCCAAACCCACTGTGAACC--TACC---ACT-GTTG

CTTCGGCGG-ATCCGCCCC-GGGCGCACCCT-TC-AGGGGG-TGT--GCCCCGGAA-CCA

-GGCGCCCGCCGGGGG---ACCGAAACCTCT-GTATCTT----CAGTTGAGTTA--CTCT

GAGTGT--GATTT---------CTAAAATCAAAATTAAAACTTTCAACAACGGATCTCTT

GGCTCTAGCATCGATGAAGAACGCAGCGAAATGCGATAAGTAATGCGAATTGCAGAATTC

AGTGAATCATCGAATCTTTGAACGCACATGGCGCCCGCTAGTATTCTGGCGGGCATGCCT

GTCTGAGCGTCGTTTCGACCCTCGCCCCC-GGCG---TCT----GTCGGGGG--------

-----CGGTGTTGGGGAT-CGGCC--ACCACCC-TTCA-CT-----GGGCGG-CCGTCCC

C-TAAATTCAGTGGCGACCACGCTGTAGCC-TCCCCTGCGTAGTACTAAAACCACCTC--

GCAGGCGGAG--AGCGGTGCGGCC-CGCCGTAAAACCCCCCAAACT-TTTA-CAA-AGTA

ACGGCGA-GTGAAGCGGCAAAAGCTCAAATT-TGAAATCT-GGCC-TCT---GGCCCG-A

GTTGTAATTTGCAGAGGATGTTTCTGGCGAGGTGCCTTCCGAGTTCCCTGG-AACGGGAC

GCCATAGAGGGTGAGAGCCCCGTACGGTTGGTCGC--TAAGCCTCTGTGAAACTCCTTCG

ACGAGTCGAGTAGTTTGGGAATGCTGCTCTAAATTGGAGGTGTACGCCTTCTAAAGCTAA

ATACAGGCTAGAGACCGATAGCGCACAAGTAGAGTGATCGAAAGATGAAAAGCACTTTGA

AAAGAGGGTTAAAAAGTACGTGAAATTGTTGAAAGGGAAGCGCTCTTGACCAGACTTGGG

C-CGGTTGATCATCCGCCGTTCTCGGCGGTGCACTCT-G-CCGGCTCAGGCCAGCATCAG

CTCGGTACGGGGGATAAAGGCTCCGGGAATGTGGCTCTCTCC--GGGGAGTGTTATAGCC

CGCTGCGTAATACCCTGCACTGGGCTGAGGTTCGCGCATCTGCATGGATGCTGGCGTAAT

GGTCATCAGTGACCCGTCTTAGACAGCTGCACAACACCCATTGGGGACTGGTGTGCCCAG

CCGAAACCCCAGAGGGCCAGGCATGTGGTCTGGTCAAGAACTTATCGCTGATGTGCTACG

TCAGTGTCGGATCGCCATCCGATCCTCTGATTGACTTCATGATCAACCGTGGTATGGAAG

TTGTGGAAGAGTACGAGCCACTCAGATACCCGCACGCAACAAAGATCTTCATCAACGGAA

GCTGGGTTGGTGTGCACCAGGACCCCAAGCATCTCGTCAGCCATGTTCTCGATCTTCGCC

GAAAGTCGTACCTGCCGTACGAGGTATCTCTCGTGCGCGATATCCGCGACCGAGAATTCA

AGATCTTTTCAGATGCTGGCCGTGTTATGCGTCCCGTTTTCACGGTTCGACAAGAAGACG

GTC-AGGTCGGCGACCCTGCGAAGGGCTCACTCGTCCTGTCAAAGGAGCTCGTCAACCAG

CTTGCCAAGGAGCAG---GCT-GAG-CCGG----------AGA-CGGGCGAGTCGAAGTC

TGGCTGGGATCGCCTTATTGGTGCTGGCGCAGTTGAGTACCTCGATGCTGAGGAGGAGGA

GACAGCCATGATTTGCATGACCCCCGAAGACTTGGAGTCGTATCGTATCCAGAAGCT---

------------------------------GGGTTACGCTGCACCC------GACGAGGG

TGAGGAGCTAGAGCCTAACAAACGATTGAGGACAAAGACAAACCCGACAACCCATATGTA

CACCCATTGCGAGATTCATCCCAGTATGATCCAAGGATGGCCAGACTCGTGAGCACGCTC

TCCTTGCCTACACCCTGGGTGTCAAGCAGCTCATCGTTGCCATCAACAAGATGGACACTG

CC-AACTGGGCTGAGGCTCGTTTCAACGAAATCATCAAGGAGA--CCTCCAACTTCATCA

AGAAGGTCGGCTACAACCCCAAGACTGTTGCCTTCGTCCCCATCTCTGGTTTCCACGGCG

ACAACATGCTTGCCC----CCACCACCAACGCTCCCTGGTACAAGGGTTGGGAGAAGGAG

ACCAAGGGCGG---CAAGTCCTCTGGCAAGACCCTCCTCGAGGCCATTGACTCCATCGAG

CCCCCCAAGCGCCCCGTCGACAAGCCCCTCCGTCTTCCTCTCCAGGATGTCTACAAGATC

GGTGGTATTGGCACAGTCCCTGTCGGCCGTATCGAGACCGGTGTCATCAAGCCCGGTATG

GTCGTTACCTTCGCCCCCTCCAACGTCACCACTGAAGTCAAGTCGGTCGAGATGCACCAC

GAGCAGCTCGCCGAGGGTGTCCCCGGTGACAACGTTGGCTTCAACGTGAAGAACGTCTCC

GTCAAGGACATCCGTCGTGGCAACGTCGCCTCCGACTCCAAGAACGACCCCGCCCAGGGC

GCTGCTTCCTTCAACGCCCAGGTCATCGTTCTCAACCACCCTGGTCAGGTCGGTGCCGGC

TACGCCCCCGTCCTCGACTGCCACACCGCCCAC-ATTGCGTGCAAGTTCTCCGAGCTCCT

CGAGAAGATCGATCGCCGTACCGGTAAGGCTGTTGAGGAGG-------------------

--------

# **References**

Bacon CW (1988) Procedure for isolating the endophyte from tall fescue and screening isolates for ergot alkaloids. Applied and Environmental Microbiology 54: 2615–2618. <https://doi.org/10.1128/aem.54.11.2615-2618.1988>

Cannon PF (2003) A Monograph of Bionectria (Ascomycota, Hypocreales, Bionectriaceae) and its Clonostachys Anamorphs by Hans-Josef Schroers (2001). Studies in Mycology 46. Pp. 214. ISBN 90-70351-44-750 (softback). Centraalbureau voor Schimmelcultures, Utrecht, The Netherlands. Price A50. Mycologist 17: 73–73. <https://doi.org/10.1017/s0269915x03272177>

Chinworrungsee M, Wiyakrutta S, Sriubolmas N, Chuailua P, Suksamrarn A (2008) Cytotoxic activities of trichothecenes isolated from an endophytic fungus belonging to order hypocreales. Archives of Pharmacal Research 31: 611–616. <https://doi.org/10.1007/s12272-001-1201-x>

Duc PM, Hatai K, Kurata O, Tensha K, Yoshitaka U, Yaguchi T, Udagawa S (2009) Fungal infection of mantis shrimp (Oratosquilla oratoria) caused by two anamorphic fungi found in Japan. Mycopathologia 167: 229–247. <https://doi.org/10.1007/s11046-008-9174-4>

Evans L, Hedger JN, Brayford D, Stavri M, Smith E, O'Donnell G, Gray AI, Griffith GW, Gibbons S (2006) An antibacterial hydroxy fusidic acid analogue from Acremonium crotocinigenum. Phytochemistry 67: 2110–2114. <https://doi.org/10.1016/j.phytochem.2006.06.033>

Gazis R, Chaverri P (2015) Wild trees in the Amazon basin harbor a great diversity of beneficial endosymbiotic fungi: is this evidence of protective mutualism? Fungal Ecology 17: 18–29. <https://doi.org/10.1016/j.funeco.2015.04.001>

Georgousaki K, Tsafantakis N, Gumeni S, González-Menéndez V, Pedro N de, Tormo JR, Almeida C, Lambert C, Genilloud O, Trougakos IP, Fokialakis N (2019) Cercospora sp. as a source of anti-aging polyketides targeting 26S proteasome and scale-up production in submerged bioreactor. Journal of Biotechnology 301: 88–96. <https://doi.org/10.1016/j.jbiotec.2019.05.015>

Grum-Grzhimaylo AA, Георгиева МЛ, A.J.M. Debets, Биланенко ЕН (2013) Are alkalitolerant fungi of the Emericellopsis lineage (Bionectriaceae) of marine origin? IMA Fungus 4: 213–228. <https://doi.org/10.5598/imafungus.2013.04.02.07>

Kiyuna T, An K-D, Kigawa R, Sano C, Miura S, Sugiyama J (2011) Molecular assessment of fungi in "black spots" that deface murals in the Takamatsuzuka and Kitora Tumuli in Japan: Acremonium sect. Gliomastix including Acremonium tumulicola sp. nov. and Acremonium felinum comb. nov. Mycoscience 52: 1–17. <https://doi.org/10.1007/s10267-010-0063-6>

Konovalova O, Bubnova E (2011) Fungi on brown seaweeds ascophyllum nodosum and pelvetia canaliculata in the kandalaksha bay of white sea. Микология и фитопатология 45: 240–248. Available from: <https://www.researchgate.net/publication/286580148_Fungi_on_brown_seaweeds_ascophyllum_nodosum_and_pelvetia_canaliculata_in_the_kandalaksha_bay_of_white_sea>

Lo Piccolo S, Alfonzo A, Giambra S, Conigliaro G, Lopez-Llorca LV, Burruano S (2015) Identification of Acremonium isolates from grapevines and evaluation of their antagonism towards Plasmopara viticola. Annals of Microbiology 65: 2393–2403. <https://doi.org/10.1007/s13213-015-1082-5>

Marincowitz S, Crous PW, Groenewald JZ, Wingfield MJ (2008) Microfungi Occurring on Proteaceae in the Fynbos. CBS Biodiversity Series

Mbenoun M, Wingfield MJ, Begoude Boyogueno AD, Wingfield BD, Roux J (2013) Molecular phylogenetic analyses reveal three new Ceratocystis species and provide evidence for geographic differentiation of the genus in Africa. Mycological Progress 13: 219–240. <https://doi.org/10.1007/s11557-013-0907-5>

Mouton M, Postma F, Wilsenach J, Botha A (2012) Diversity and Characterization of Culturable Fungi from Marine Sediment Collected from St. Helena Bay, South Africa. Microbial Ecology 64: 311–319. <https://doi.org/10.1007/s00248-012-0035-9>

Okada G, Hashimoto A, Ohkuma M (2022) JCM Catalogue. Riken.jp. Available from: <https://www.jcm.riken.jp/cgi-bin/jcm/jcm_keyword?AN=Trichothecium&BN=sympodiale&CN=&DN=> (April 14, 2026).

Ondeyka JG, Zink D, Basilio A, Vicente F, Bills G, Diez MT, Motyl M, Gabe Dezeny, Byrne K, Singh SB (2007) Coniothyrione, a Chlorocyclopentandienylbenzopyrone as a Bacterial Protein Synthesis Inhibitor Discovered by Antisense Technology. Journal of Natural Products 70: 668–670. <https://doi.org/10.1021/np060557d>

Peláez F, Cabello A, Platas G, Díez MT, González del Val A, Basilio A, Martán I, Vicente F, Bills GE, Giacobbe RA, Schwartz RE, Onish JC, Meinz MS, Abruzzo GK, Flattery AM, Kong L, Kurtz MB (2000) The discovery of enfumafungin, a novel antifungal compound produced by an endophytic Hormonema species biological activity and taxonomy of the producing organisms. Systematic and Applied Microbiology 23: 333–343. <https://doi.org/10.1016/s0723-2020(00)80062-4>

Perdomo H, Sutton D, García D, Fothergill AW, Cano J, Gené J, Summerbell RC, Rinaldi M, Guarro J (2011) Spectrum of Clinically Relevant Acremonium Species in the United States. Journal of Clinical Microbiology 49: 243–256. <https://doi.org/10.1128/jcm.00793-10>

Poling SM, Wicklow DT, Rogers KD, Gloer JB (2008) Acremonium zeae, a Protective Endophyte of Maize, Produces Dihydroresorcylide and 7-Hydroxydihydroresorcylides. Journal of Agricultural and Food Chemistry 56: 3006–3009. <https://doi.org/10.1021/jf073274f>

Ropars J, Cruaud C, Lacoste S, Dupont J (2012) A taxonomic and ecological overview of cheese fungi. International Journal of Food Microbiology 155: 199–210. <https://doi.org/10.1016/j.ijfoodmicro.2012.02.005>

Schoch CL, Robbertse B, Robert V, Vu D, Cardinali G, Irinyi L, Meyer W, Nilsson RH, Hughes K, Miller AN, Kirk PM, Abarenkov K, Aime MC, Ariyawansa HA, Bidartondo M, Boekhout T, Buyck B, Cai Q, Chen J, Crespo A (2014) Finding needles in haystacks: linking scientific names, reference specimens and molecular data for Fungi. Database 2014: bau061–bau061. <https://doi.org/10.1093/database/bau061>

Seifert KA, Rehner SA, Sugita T, Okada G (2008) Spicellum ovalisporum sp. nov. Fungal Planet 28. Available from: <https://www.researchgate.net/publication/285915226_Spicellum_ovalisporum_Seifert_Rehner_sp_nov>

Suay I, Arenal F, Asensio FJ, Basilio A, Angeles Cabello M, Teresa Díez M, García JB, González del Val A, Gorrochategui J, Hernández P, Peláez F, Francisca Vicente M (2000) Screening of basidiomycetes for antimicrobial activities. Antonie van Leeuwenhoek 78: 129–140. <https://doi.org/10.1023/A:1026552024021>

Tang AMC, Jeewon R, Hyde KD (2006) Phylogenetic utility of protein (RPB2, β-tubulin) and ribosomal (LSU, SSU) gene sequences in the systematics of Sordariomycetes (Ascomycota, Fungi). Antonie van Leeuwenhoek 91: 327–349. <https://doi.org/10.1007/s10482-006-9120-8>

Vu D, Groenewald M, de Vries M, Gehrmann T, Stielow B, Eberhardt U, Al-Hatmi A, Groenewald JZ, Cardinali G, Houbraken J, Boekhout T, Crous PW, Robert V, Verkley GJM (2019) Large-scale generation and analysis of filamentous fungal DNA barcodes boosts coverage for kingdom fungi and reveals thresholds for fungal species and higher taxon delimitation. Studies in Mycology 92: 135–154. <https://doi.org/10.1016/j.simyco.2018.05.001>

Wicht B, Petrini O, Jermini M, Gessler C, Antonio G (2012) Molecular, proteomic and morphological characterization of the ascomycete Guignardia bidwellii, agent of grape black rot: a polyphasic approach to fungal identification. Mycologia 104: 1036–1045. <https://doi.org/10.2307/23488812>

Wicklow DT, Poling SM (2009) Antimicrobial Activity of Pyrrocidines from Acremonium zeae Against Endophytes and Pathogens of Maize. Phytopathology 99: 109–115. <https://doi.org/10.1094/phyto-99-1-0109>

Witthuhn RC, Wingfield BD, Wingfield MJ, Harrington TC (1999) PCR-based identification and phylogeny of species of Ceratocystis sensu stricto. Mycological Research 103: 743–749. <https://doi.org/10.1017/s0953756298007679>

Xing X, Guo S (2010) Fungal endophyte communities in four Rhizophoraceae mangrove species on the south coast of China. Ecological Research 26: 403–409. <https://doi.org/10.1007/s11284-010-0795-y>

Zhang G, Tang L, Liu H, Liu D, Wang M, Cai J, Liu W, Nie W, Zhang Y, Yu X (2021) Psidium guajava Flavonoids Prevent NLRP3 Inflammasome Activation and Alleviate the Pancreatic Fibrosis in a Chronic Pancreatitis Mouse Model. The American Journal of Chinese Medicine 49: 2001–2015. <https://doi.org/10.1142/s0192415x21500944>

Zhang Q, Zhang J, Yang L, Zhang L, Jiang D, Chen W, Li G (2014) Diversity and biocontrol potential of endophytic fungi in Brassica napus. Biological Control 72: 98–108. <https://doi.org/10.1016/j.biocontrol.2014.02.018>

Zhang R, Li P, Zhao J, Wang J, Peng Y, Zhou L (2008) Diosgenin production of Dioscorea zingiberensis cultures stimulated by its endophytic fungi. Journal of Biotechnology 136: S151. <https://doi.org/10.1016/j.jbiotec.2008.07.323>

Zhao L, Groenewald JZ, Hou LW, Summerbell RC, Crous PW (2025) Bionectriaceae : a poorly known family of hypocrealean fungi with major commercial potential. Studies in Mycology 111: 115–392. <https://doi.org/10.3114/sim.2025.111.04>

Zhao L, Groenewald JZ, Hou LW, Starink-Willemse M, Beek B, Grum-Grzhimaylo OA, Summerbell RC, Crous PW (2026) New insights into acremonium-like fungi in Hypocreales : A taxonomic and phylogenetic perspective. Studies in Mycology 113: 1–71. <https://doi.org/10.3114/sim.2026.113.01>

Zuccaro A, Summerbell R, Mitchell J (2004) A new Acremonium species associated with Fucus spp., and its affinity with a phylogenetically distinct marine Emericellopsis clade. Studies in Mycology 50: 283–297
